# Polyimide‐Linked Hexaazatriphenylene‐Based Porous Organic Polymer with Multiple Redox‐Active Sites as a High‐Capacity Organic Cathode for Lithium‐Ion Batteries

**DOI:** 10.1002/adma.202512950

**Published:** 2025-10-16

**Authors:** Arindam Mal, Jonathan Caroni, Asia Patriarchi, Olivera Luzanin, Rafael Ramos, Jan Bitenc, Manuel Melle‐Franco, Manuel Souto

**Affiliations:** ^1^ CiQUS Centro Singular de Investigación en Química Bioloxica e Materiais Moleculares Departamento de Química‐Física Universidade de Santiago de Compostela Santiago de Compostela 15782 Spain; ^2^ Chemistry Division School of Science and Technology University of Camerino Via Madonna delle Carceri‐ChIP Camerino MC 62032 Italy; ^3^ National Institute of Chemistry Hajdrihova 19 Ljubljana 1000 Slovenia; ^4^ Department of Chemistry CICECO‐Aveiro Institute of Materials University of Aveiro Aveiro 3810‐393 Portugal; ^5^ Oportunius Galician Innovation Agency (GAIN) Santiago de Compostela 15702 Spain

**Keywords:** covalent organic frameworks, high‐capacity organic cathode, lithium‐ion batteries, organic batteries, organic electrode materials, porous organic polymers

## Abstract

The development of high‐capacity, sustainable cathode materials remains a critical challenge in advancing lithium‐ion battery technologies for next‐generation energy storage. Organic electrode materials (OEMs) represent a promising alternative to conventional inorganic cathodes, owing to their composition from earth‐abundant elements and chemically tunable structures that enable high theoretical capacities. Herein, a polyimide‐linked porous organic polymer (HAT‐PTO) is reported to be synthesized via a straightforward hydrothermal reaction from redox‐active hexaazatriphenylene (HAT) and pyrene‐4,5,9,10‐tetraone (PTO) building blocks. The resulting HAT‐PTO framework incorporates multiple redox‐active C═O and C═N centers, delivering a high theoretical capacity of 484 mAh g^−1^. To overcome limitations in electronic conductivity, hybrid materials are synthesized by in situ growth of HAT‐PTO on multiwalled pristine (CNT) and carboxyl‐functionalized carbon nanotubes (cCNT). Notably, the HAT‐PTO‐cCNT hybrid delivers a high capacity of 397 mAh g^−1^ at C/10, outstanding rate capability of 225 mAh g^−1^ at 20 C, and long‐term cycling stability, retaining 171 mAh g^−1^ after 6000 cycles at 2 C. Ex situ FT‐IR, supported by density functional theory (DFT) calculations, confirms the involvement of both HAT and PTO units in the charge storage mechanism. This work presents a molecular design strategy and scalable synthesis approach toward high‐performance organic cathodes, paving the way for durable, high‐rate lithium‐organic batteries.

## Graphical abstract

A high‐capacity polyimide‐linked porous organic polymer (HAT‐PTO) incorporating numerous redox‐active centers is synthesized via a hydrothermal reaction, delivering a high theoretical capacity of 484 mAh g^−1^. In situ hybridization with carboxyl‐functionalized multiwalled carbon nanotubes enhances conductivity and stability, achieving 397 mAh g^−1^ at C/10, outstanding rate capability (225 mAh g^−1^ at 20 C), and robust cycling over 6000 cycles.

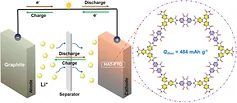

## 1 Introduction

Lithium‐ion batteries (LIBs) are among the most promising technologies for sustainable energy storage, owing to their high energy density, low self‐discharge rate, and long cycle life.^[^ ^1^, ^2^ ^]^ LIBs are widely used in portable electronic devices and are being adopted in the rapidly growing electric vehicle industry.^[^ ^3^, ^4^ ^]^ The demand for enhanced performance in high‐power applications necessitates the development of new materials that can increase practical energy densities, support high current rates, and enable rapid charging and discharging without compromising cycling stability. The performance of LIBs is largely dependent on the properties of the cathode materials.^[^ ^5^ ^]^ However, conventional cathode materials are typically derived from inorganic transition metal oxides or phosphates (LiCoO_2_, LiMn_2_O_4_, LiFePO_4_, etc.), which present limitations in terms of theoretical specific capacity, resource availability, and reliance on the mining of critical raw materials.^[^ ^6^ ^]^ Organic electrode materials (OEMs) have long been proposed as promising alternatives to inorganic cathodes for the development of sustainable batteries, as they are composed of naturally abundant elements (C, H, N, O, etc.) and offer tunable electrochemical performance through chemical design, enabling high specific capacities by introducing multiple redox‐active sites.^[^ ^7^, ^8^, ^9^, ^10^, ^11^, ^12^, ^13^ ^]^

Lithium‐organic batteries are therefore regarded as a promising class of next‐generation energy storage devices due to their tunable electrochemical properties, environmental friendliness, cost‐effectiveness, and flexibility of organic electrodes. However, OEMs face significant challenges, including low intrinsic electronic conductivity and gradual dissolution in electrolytes, particularly for small redox‐active organic molecules.^[^ ^8^, ^10^ ^]^ Low electronic conductivity is commonly addressed by adding conductive additives, although their content must be carefully optimized to avoid reducing the effective capacity.^[^ ^12^, ^14^ ^]^ To address dissolution, polymerizing small electroactive molecules has proven to be an effective strategy for enhancing cycling stability and minimizing solubility in electrolytes.^[^ ^15^, ^16^ ^]^

Porous organic polymers (POPs) and covalent organic frameworks (COFs) have emerged as promising OEMs for lithium‐organic batteries due to their intrinsic insolubility in electrolytes, extensive structural and chemical versatility, tunable porosity, and the ability to incorporate numerous redox‐active centers.^[^ ^17^, ^18^, ^19^ ^]^ Both POPs and COFs are purely organic porous materials constructed by combining organic building blocks linked through strong covalent bonds,^[^ ^20^, ^21^, ^22^ ^]^ with the redox activity and electrochemical stability of the resulting materials depending on the nature of the electroactive organic moieties and linkages.^[^ ^23^ ^]^ Extensive research has been devoted to combining multiple redox centers into the same POPs to maximize their practical capacity. For example, π‐conjugated hexaazatriphenylene (HAT)‐based POPs have attracted significant interest as OEMs, mainly due to their high theoretical capacity arising from multiple redox‐active pyrazine moieties.^[^ ^24^, ^25^, ^26^, ^27^, ^28^, ^29^, ^30^, ^31^, ^32^ ^]^ Pyrene‐4,5,9,10‐tetraone (PTO) has also been investigated as an n‐type building block for the construction of high‐capacity OEMs, as all four carbonyl groups can participate in the redox process (4 e^−^/Li^+^).^[^ ^33^, ^34^, ^35^, ^36^, ^37^, ^38^ ^]^ Recently, polyimide‐linked POPs and COFs have also attracted considerable interest as robust OEMs for battery applications, featuring additional carbonyl redox‐active sites along with excellent electrochemical stability and reversibility.^[^ ^28^, ^39^, ^40^, ^41^, ^42^ ^]^ In some cases, hybridization of porous polymers with carbon nanotubes (CNTs) can enhance electrochemical performance by improving both electronic conductivity and cycling stability,^[^ ^27^, ^29^, ^35^ ^]^ although the content and nature of CNTs must be carefully optimized. Very recently, a series of arylamine‐linked HAT‐based POPs have been reported with theoretical capacities of up to 440 mAh g^−1^.^[^ ^30^ ^]^ However, achieving a higher theoretical capacity, current density, and long‐term cycling stability in POP‐based OEMs remains a key challenge in the development of lithium‐organic batteries.

Herein, a new polyimide‐linked POP (**HAT‐PTO**) based on redox‐active HAT and PTO building blocks has been designed and synthesized via a straightforward hydrothermal reaction. **HAT‐PTO** contains abundant redox‐active groups (C═O and C═N) from HAT, PTO, and imide moieties, resulting in one of the highest reported theoretical capacities (484 mAh g^−1^) for POPs and COFs.^[^ ^18^ ^]^ In addition, different hybrids of **HAT‐PTO** with multiwalled carbon nanotubes (CNT) or carboxyl‐functionalized carbon nanotubes (cCNT) were synthesized via in situ growth of the POP on the surface of the CNTs to enhance the electrochemical performance. The **HAT‐PTO‐cCNT** hybrid demonstrated a high specific capacity of 397 mAh g^−1^ at C/10, excellent rate performance (225 mAh g^−1^ at 20 C), and long‐cycling stability (171 mAh g^−1^ capacity retained after 6000 cycles at 2 C). This work presents a new molecular design approach and synthetic strategy for developing OEMs with high theoretical capacity, offering an efficient route toward high‐performance organic cathodes for lithium batteries.

## 2 Results and Discussion

### 2.1 Synthesis and Characterization

**Figure 1:**
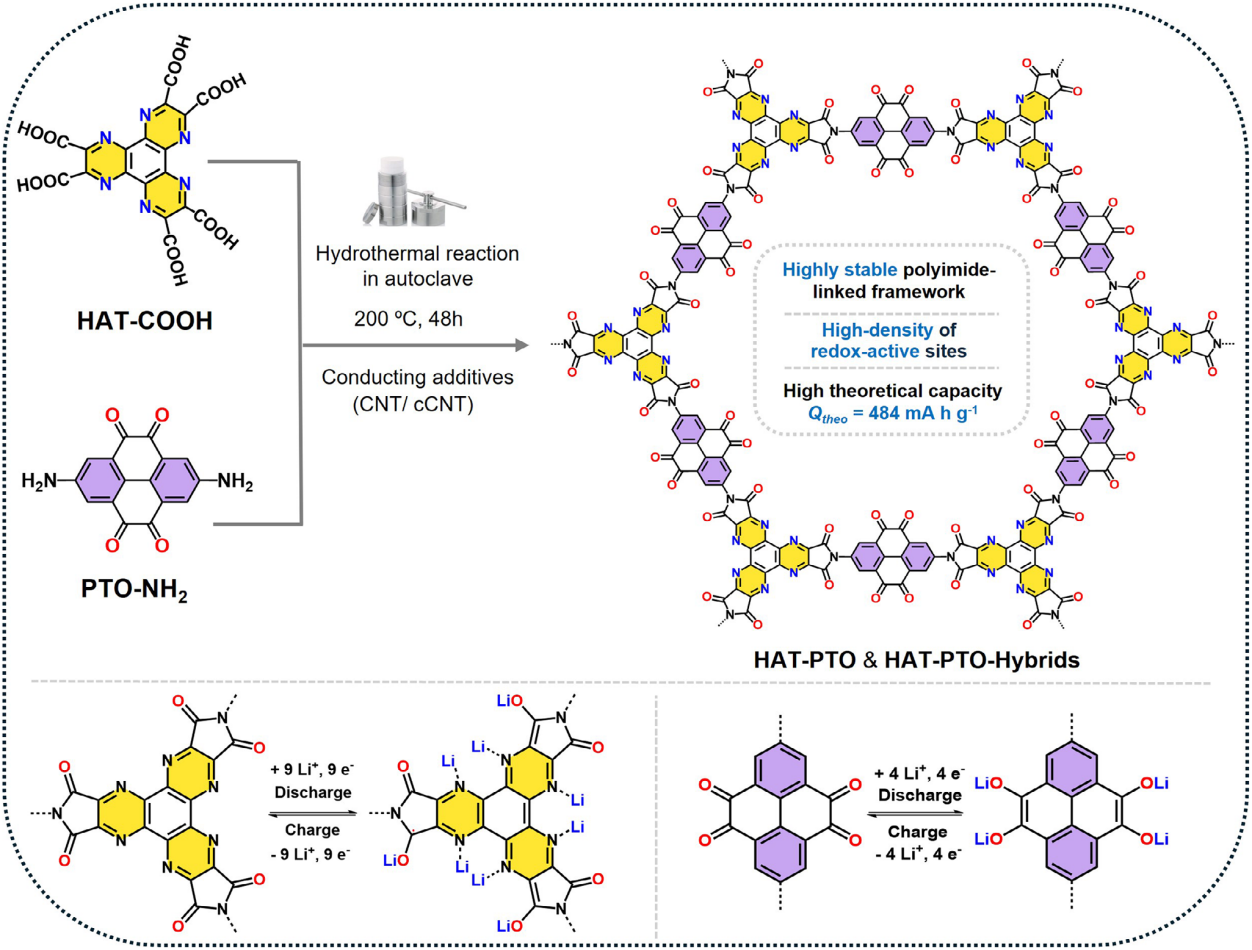
Schematic representation of the synthesis of **HAT‐PTO** and redox reaction mechanism of the HAT and PTO moieties.

Imide‐linked HAT‐PTO was synthesized via a condensation reaction between hexaazatriphenylene hexacarboxylic acid (HAT‐6COOH) and 2,7‐diaminopyrene‐4,5,9,10‐ tetraone (PTO‐2NH_2_) under hydrothermal conditions (200 °C for 48 h) (**Figure** **1**). According to the design of the imide‐linked POP, HAT‐PTO offers a high theoretical capacity of 484 mAh g^−1^, attributed to the presence of both redox‐active moieties (HAT and PTO) and polyimide linkages containing a high density of carbonyl and imine groups (Figure 1).

**Figure 2:**
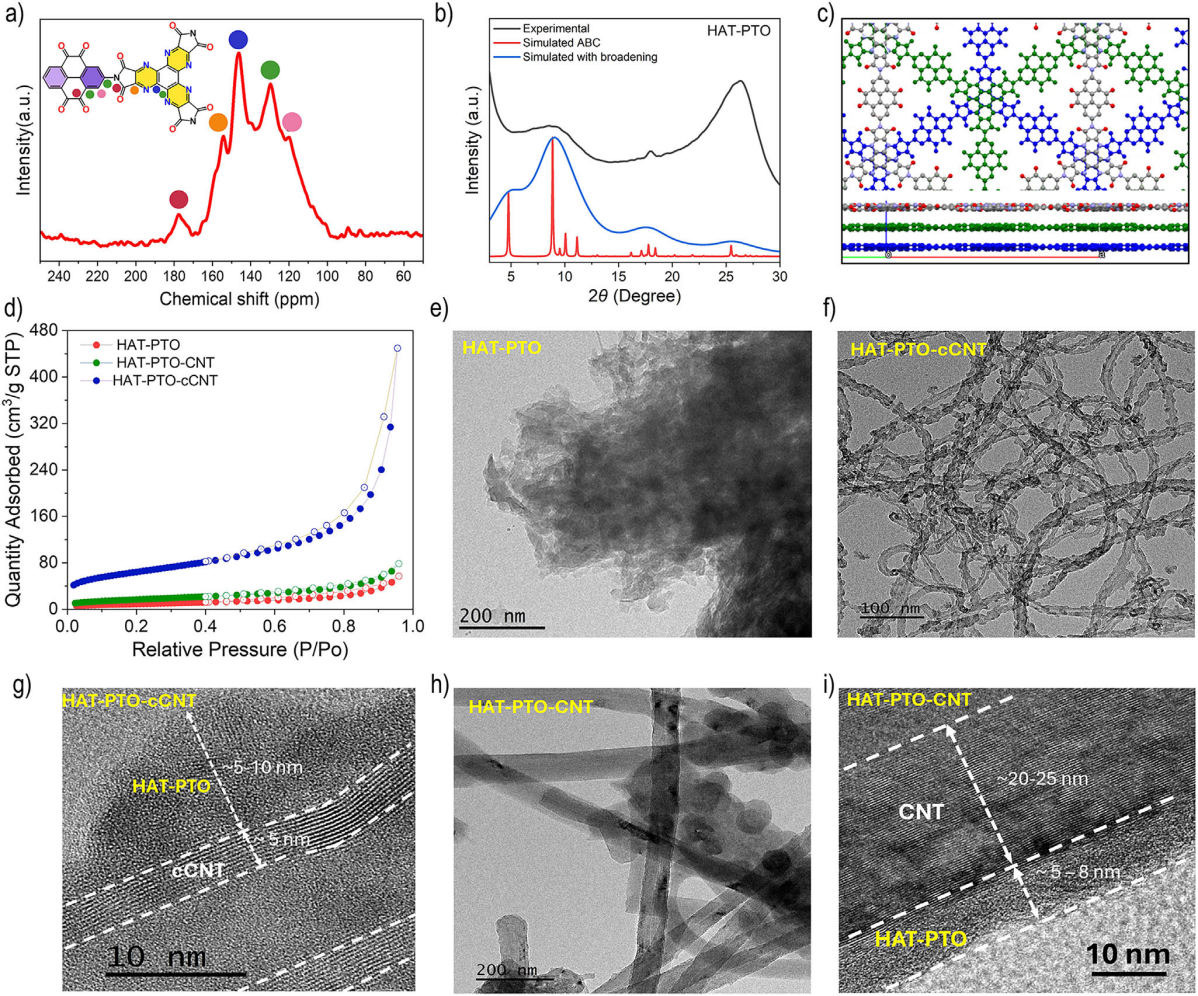
a) Solid‐state ^13^C CP‐MAS NMR spectrum, b) experimental and simulated (with and without broadening effect) PXRD patterns, and c) top and side views of the computational model of HAT‐PTO with ABC packing. d) N_2_ adsorption (filled symbols) and desorption (empty symbols) isotherms of HAT‐PTO, HAT‐PTO‐CNT, and HAT‐PTO‐cCNT at 77 K. e) HRTEM image of HAT‐PTO. f,g) HRTEM images of HAT‐PTO‐cCNT. h,i) HRTEM images of HAT‐PTO‐CNT. Different highlighted areas represent various domains of cCNT or CNT and HAT‐PTO within the hybrids. Scale bars are included in the insets.

The formation of imide linkages in HAT‐PTO was confirmed by ^13^C solid‐state cross‐polarization magic‐angle spinning (CP‐MAS) NMR and Fourier transform infrared (FT‐IR) spectroscopies. The ^13^C solid‐state CP‐MAS NMR analysis reveals broad signals at ca. 177.5 and 172.4 ppm, corresponding to the carbonyl (C═O) carbons of the imide and PTO units, respectively (**Figure** **2**a).^[^ ^29^, ^35^, ^40^ ^]^ Additional ^13^C NMR peaks observed at 154.1, 146.3, 129.6, and 119.9 ppm are attributed to the aromatic carbon atoms of the HAT‐PTO framework, as annotated in Figure 2a.^[^ ^40^ ^]^ The FT‐IR spectrum of HAT‐PTO shows a characteristic band at 1664 cm^−1^, attributed to the C═O stretching vibrations from both the PTO and imide units.^[^ ^28^, ^43^ ^]^ The formation of imide linkages is further supported by the presence of a band at 1362 cm^−1^, assigned to the C─N stretching vibrations of the imide moieties (Figure S1, Supporting Information).^[^ ^28^, ^43^ ^]^ Comparative FT‐IR analysis confirms the complete consumption of the precursor building blocks, as the characteristic bands related to ─NH_2_ and ─COOH groups are absent in the HAT‐PTO IR spectrum. The experimental powder X‐ray diffraction (PXRD) pattern of the synthesized HAT‐PTO (Figure 2b) exhibits broad diffraction peaks centered at 2ϑ = 8.9°, 17.9°, and 26.7°. The intense peak at 26.7° is characteristic of strong *π*–*π* stacking within the 2D layer structure, corresponding to an interlayer distance of *d* ≈ 3.3 Å, consistent with values reported for related systems.^[^ ^29^, ^30^ ^]^ Although the overall broadness of the PXRD pattern indicates low crystallinity, two additional broad peaks are discernible at 2ϑ = 8.9° and 17.9°, suggesting some degree of structural order. To gain further insight into the stacking arrangement, computational models representing different stacking modes (AA, AB, and ABC) were evaluated (Figure S2, Supporting Information). Among these, the ABC stacking model (Figure 2c) showed the best agreement with experimental PXRD data. Specifically, the simulated PXRD pattern, which includes peak broadening effects (Figure 2b; Figure S3, Supporting Information), reproduces the three key features observed in the experimental pattern. This assignment is further supported by the low porosity of the material (see below), which is consistent with the ABC stacking mode. Nonetheless, given the amorphous nature of the material, alternative stacking arrangements cannot be definitively excluded.

The porosity of HAT‐PTO was investigated by N_2_ adsorption isotherms at 77 K. HAT‐PTO exhibits a typical type III isotherm, with a Brunauer–Emmett–Teller (BET) surface area of 33 m^2^ g^−1^ (Figure 2d). The corresponding pore size distribution (Figure S4, Supporting Information) is consistent with those of structurally related materials.^[^ ^29^, ^30^ ^]^ Scanning electron microscopy (SEM) images of HAT‐PTO reveal a nanowire‐type morphology, likely arising from strong interlayer *π*–*π* stacking interactions (Figure S5, Supporting Information). Scanning transmission electron microscopy (STEM) and energy‐dispersive X‐ray spectroscopy (EDX) mapping confirm the homogeneous distribution of the constituent elements (Figures S6 and S7, Supporting Information). High‐resolution transmission electron microscopy (HRTEM) images further demonstrate the presence of typical 2D layered structures (Figure 2e). Thermogravimetric analysis (TGA) indicates that HAT‐PTO is thermally stable up to 300 °C, with a minor weight loss (ca. 5%) below 110 °C, attributed to the release of adsorbed water molecules (Figure S8, Supporting Information).

Following the same synthetic procedure, nonfunctionalized carbon nanotubes (CNTs) and carboxyl‐functionalized carbon nanotubes (cCNTs) were incorporated into the reaction mixture to prepare HAT‐PTO‐based hybrids containing 43% CNT or cCNT (Figure 1 and Supporting Information). The resulting hybrids were characterized by various techniques. Notably, the physical nature of the hybrids differs depending on the type of nanotube: HAT‐PTO‐CNT exhibits a soft, flake‐like morphology, whereas HAT‐PTO‐cCNT appears more granular. PXRD patterns of both hybrids display similar features to HAT‐PTO, with a dominant peak at 26° (Figure S9, Supporting Information), indicating preserved *π*–*π* stacking. Compared to HAT‐PTO, the hybrids exhibited enhanced porosity, with calculated BET surface areas of 61 and 219 m^2^ g^−1^ for HAT‐PTO‐CNT and HAT‐PTO‐cCNT, respectively (Figure 2d), consistent with the higher intrinsic porosity of cCNT (361 m^2^ g^−1^) compared to CNT (48 m^2^ g^−1^) (Figure S10, Supporting Information). Both hybrids exhibit pore size distributions comparable to that of HAT‐PTO (Figure S11, Supporting Information).

The morphologies of the HAT‐PTO‐CNT hybrids were further investigated using SEM and HRTEM microscopies. HAT‐PTO‐cCNT displays a uniform tubular core‐shell morphology, with HAT‐PTO uniformly grown and well distributed along the surface of the cCNTs (Figure 2f,g; Figures S12 and S13, Supporting Information). In contrast, HAT‐PTO‐CNT exhibits a heterogeneous morphology, featuring a combination of tubular core‐shell structures and irregularly stacked layered domains of HAT‐PTO (Figure 2h,i; Figures S14 and S15, Supporting Information). However, the surface‐assisted growth of HAT‐PTO on CNT appears thinner and less uniform than on cCNTs. The morphological differences are attributed to the higher dispersibility of cCNTs in water, which facilitates more effective heterogeneous nucleation and stronger *π*–*π* interactions, ultimately promoting uniform growth of HAT‐PTO and the formation of well‐defined HAT‐PTO‐cCNT hybrids. In contrast, the simultaneous occurrence of both homogeneous and heterogeneous nucleation in the presence of CNTs led to random structural growth of HAT‐PTO. Well‐defined lattice fringes with an interlayer spacing of 3.3 Å were observed, consistent with *π*–*π* stacking and indicative of the presence of carbon nanotubes in the hybrids. Furthermore, the uniform distribution of carbon, nitrogen, and oxygen across the CNT surfaces was confirmed by STEM and corresponding EDX mapping images (Figures S16 and S17, Supporting Information), supporting surface‐assisted growth of HAT‐PTO on carbon nanotubes, as pristine CNTs do not contain nitrogen. TGA analysis demonstrated that the hybrids exhibit high thermal stability, retaining structural integrity up to 400 °C (Figure S8, Supporting Information). Room‐temperature electrical conductivities of HAT‐PTO, HAT‐PTO‐CNT, and HAT‐PTO‐cCNT as pressed pellets were measured using a four‐probe van der Pauw configuration (see Supporting Information for experimental details). The obtained conductivity values were 1.95 × 10^−7^, 3.09 × 10^1^ and 3.40 S cm^−1^ for HAT‐PTO, HAT‐PTO‐CNT, and HAT‐PTO‐cCNT (Tables S2 and S3 and Figures S18 and S19, Supporting Information), respectively, demonstrating a significant enhancement in electronic conductivity for the hybrids incorporating CNTs or cCNTs.

### 2.2 Electrochemical Performance

The electrochemical performance of HAT‐PTO, HAT‐PTO‐CNT, and HAT‐PTO‐cCNT was evaluated as cathode materials for Li‐ion storage, motivated by the high theoretical capacity of HAT‐PTO (484 mAh g^−1^) (Scheme S2, Supporting Information). The working electrodes were prepared by mixing the active material (HAT‐PTO), conductive carbon black, and polyvinylidene fluoride (PVDF) binder in a mass ratio of 60:30:10. For HAT‐PTO‐CNT and HAT‐PTO‐cCNT hybrids, a mass ratio of 80:10:10 was used. The mass loading of active material in the electrodes was in the range of 0.5–0.8 mg cm^−2^. As is standard practice for organic batteries, specific capacities were calculated based on the mass of the active material (HAT‐PTO). Electrochemical measurements were conducted in coin‐type half‐cells, with Li metal used as both the counter and reference electrodes, and a potential window of 1.2–4.0 V. The charging voltage window was limited to 4.0 V to prevent electrolyte degradation. The electrode's electrochemical stability within this range was assessed by linear sweep voltammetry (LSV) and chronoamperometry (Figure S20, Supporting Information). All the measurements were performed using 1 M lithium bis(trifluoromethanesulfonyl)imide (LiTFSI) dissolved in a 1:1 (v/v) mixture of dioxolane (DOL) and dimethoxyethane (DME) as the electrolyte. The prepared electrodes were confirmed to be insoluble in the used electrolyte, as demonstrated by UV–vis spectroscopy and gas chromatography (Figures S21–S23, Supporting Information).

**Figure 3:**
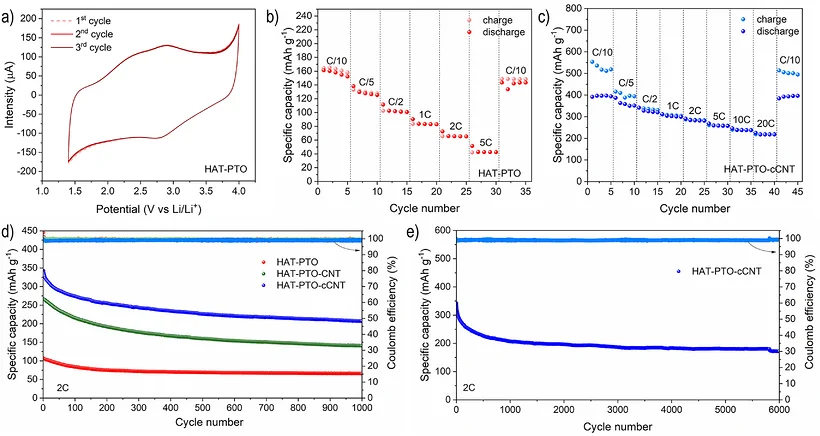
a) CV curves of the first three cycles of HAT‐PTO in the range of 1.4–4.0 V (scan rate: 1.0 mV s^−1^). b) Rate capability performance of HAT‐PTO as a function of cycle number using a potential window of 1.2–4.0 V, with current rates (C‐rates) from C/10 up to 5C. c) Rate capability performance of HAT‐PTO‐cCNT as a function of cycle number using a potential window of 1.2–4.0 V, with current rates (C‐rates) from C/10 up to 20C. d) Cycling performances of HAT‐PTO, HAT‐PTO‐CNT, and HAT‐PTO‐cCNT at 2C. e) Long‐term cycling stability of HAT‐PTO‐cCNT at 2C.

Cyclic voltammetry (CV) of HAT‐PTO was measured at 1.0 mV s^−1^ over the potential range of 1.4–4.0 V (**Figure** **3**a). The CV revealed two main pairs of redox peaks at 2.1/2.4 V and 2.8/2.9 V. The first pair is attributed to the insertion/extraction of Li^+^ ions at the carbonyl groups of the n‐type PTO units and imide linkages.^[^ ^37^, ^44^, ^45^ ^]^ The second redox couple can be assigned to the lithiation/delithiation process occurring in the HAT building blocks.^[^ ^27^, ^30^, ^37^ ^]^ The high reversibility of these redox processes is confirmed by the consistent peak positions and intensities across successive cycles, as demonstrated by the near‐complete overlap of the CV curves.

The electrochemical performance of the HAT‐PTO electrode was initially assessed through a rate capability test within the potential window of 1.2–4.0 V (Figure 3b). The highest discharge capacity was recorded during the first cycle at a C/10 rate (0.05 A g^−1^), reaching 161.5 mAh g^−1^. Upon increasing the current density, the electrode maintained stable performance, retaining a specific capacity of 43 mAh g^−1^ in the second cycle at a high rate of 5C. Notably, the electrode demonstrated excellent rate capability and reversibility; upon returning to the initial C/10 rate, the capacity recovered to 144 mAh g^−1^, closely approaching its original value, thereby confirming robust structural integrity and cycling stability. Despite the overall stable electrochemical performance, the material utilization of HAT‐PTO remains relatively low. Considering the high theoretical capacity of 484 mAh g^−1^, the measured capacity at C/10, and the contribution from the 30 wt.% conductive carbon black (Figure S24, Supporting Information), the actual capacity utilization at C/10 is 32% of the theoretical value. This indicates significant room for improvement in the electrochemical performance of HAT‐PTO.

**Figure 4:**
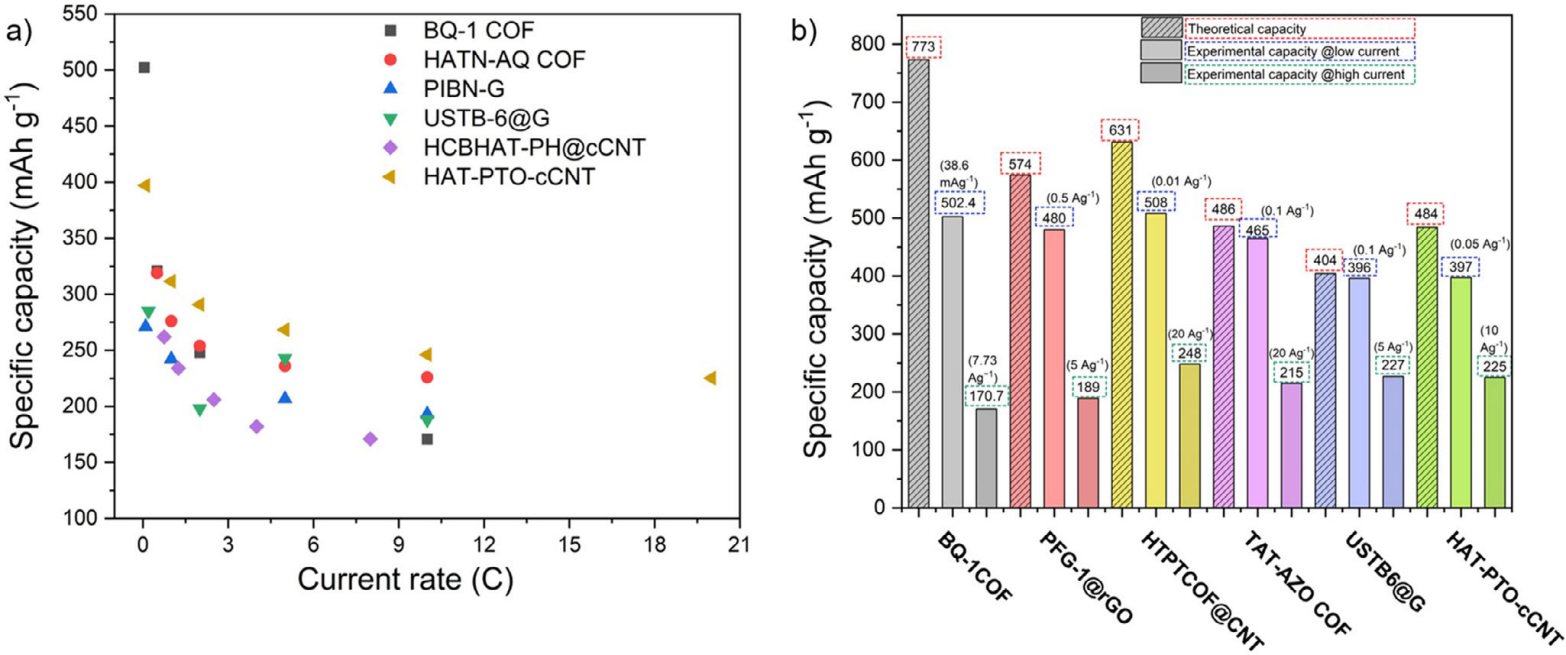
Comparison of the a) experimental capacities at different rates, and b) theoretical and experimental capacities (at low and high rates) with those of other reported high‐performance POP‐ and COF‐based organic cathodes for Li‐ion storage.

To enhance the electrochemical performance, HAT‐PTO was hybridized with CNTs or c‐CNT (with a loading of 43% CNT or cCNT) yielding HAT‐PTO‐CNT and HAT‐PTO‐cCNT composites, respectively. The rate capability test of HAT‐PTO–cCNT revealed the highest discharge capacity of 397 mAh g^−1^ at C/10 (Figure 3c). After subtracting the contribution from pure cCNTs and carbon black (Figure S25, Supporting Information) from the total measured capacity (Equation S1, Supporting Information), the intrinsic capacity of the HAT‐PTO component was determined to be 352 mAh g^−1^ at C/10. This corresponds to a utilization efficiency of 73% relative to the theoretical capacity of HAT‐PTO (Equation S2, Supporting Information), representing a substantial improvement over the pristine material. The hybrid also demonstrated excellent high‐rate performance, operating at rates up to 20C (10 A g^−1^) while maintaining a specific capacity of 225 mAh g^−1^. When the current density was returned to C/10, the capacity recovered to 395 mAh g^−1^, confirming the outstanding rate capability and reversibility of HAT‐PTO‐cCNT. To the best of our knowledge, this represents one of the highest electrochemical performances reported for POP/COF‐based organic cathodes for Li‐ion storage, particularly at high rates (Table S4, Supporting Information and **Figure** **4**).

The long‐term cycling performance of the three materials was evaluated at a current density of 2C (Figure 3d; Figure S26, Supporting Information). The HAT‐PTO electrode exhibited an initial discharge capacity of 107 mAh g^−1^, retaining 61% of its capacity after 1500 cycles, with a high Coulombic efficiency of 99.7%. Both hybrids showed improved electrochemical behavior, with initial capacities of 267 mAh g^−1^ for HAT‐PTO‐CNT and 343 mAh g^−1^ for HAT‐PTO‐cCNT, and capacity retentions of 49 and 60% after 1500 cycles, respectively. Both hybrids also maintained high Coulombic efficiencies (∼99%). The average discharge voltage was determined to be 2.1 and 2.3 V for HAT‐PTO and HAT‐PTO‐cCNT, respectively. To compare the electrochemical performance of the hybrid materials with that of the pure HAT‐PTO electrode using the same amount of active material in the electrodes, hybrids with a lower content of carbon nanotubes (HAT‐PTO‐CNT‐lc and HAT‐PTO‐cCNT‐lc) were prepared, containing 60% active material (see Figure S27, Supporting Information). HAT‐PTO‐cCNT‐lc also exhibited superior electrochemical performance, with a higher initial capacity (207 mAh g^−1^) and improved capacity retention (87%) after 500 cycles compared to HAT‐PTO‐CNT‐lc (initial capacity of 146 mAh g^−1^ and 65% capacity retention after 500 cycles). These results highlight the superior cycling stability of the cCNT‐based hybrids. One possible explanation for the higher performance in the case of HAT‐PTO–cCNT may be its higher porosity compared to HAT‐PTO–CNT, which may enhance ion diffusion, especially considering that both hybrids exhibit similar electronic conductivity. Another important distinction between the two hybrids lies in their morphology: while HAT‐PTO–cCNT exhibits a homogeneous deposition of HAT‐PTO across the cCNT surface, HAT‐PTO–CNT displays randomly stacked structures with non‐uniform HAT‐PTO deposition on the CNT surface. In addition, Raman spectroscopy confirmed the electronic coupling between the polymer and the cCNTs, as a shift in the G band from 1604 cm^−1^ in pristine cCNT to 1590 cm^−1^ in the hybrid was observed (Figure S28, Supporting Information). Then, HAT‐PTO‐cCNT was further tested over an extended cycling period at 2C (Figure 3e). HAT‐PTO‐cCNT retained a capacity of 171 mAh g^−1^ after 6000 cycles, while maintaining an excellent Coulombic efficiency of 99.2%. To further explore the HAT‐PTO‐cCNT electrolyte compatibility, we also evaluated the polymer in three additional salt/solvent combinations (Figure S29, Supporting Information), which showed a similar reversible performance to that observed using 1 M LiTFSI in DOL/DME.

### 2.3 Charge Storage Mechanism

**Figure 5:**
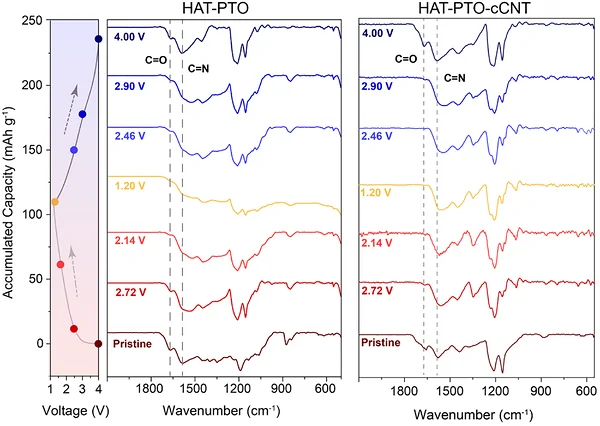
Voltage profile showing the ex situ FT‐IR sampling points and measured FT‐IR spectra of pristine (uncycled) HAT‐PTO (left) and HAT‐PTO‐cCNT (right) electrodes (bottom spectra), discharged down to 2.72, 2.14, and 1.20 V, and charged to 2.46, 2.90, and 4.00 V.

To gain further insight into the charge/discharge mechanism of the HAT‐PTO and HAT‐PTO‐cCNT cathodes, ex situ FT‐IR spectroscopy was performed at different discharge and charge states (**Figure** **5**), following the procedure detailed in the Experimental Section. The FT‐IR spectra of the pristine HAT‐PTO and HAT‐PTO‐cCNT electrodes show characteristic peaks at 1674 and 1593 cm^−1^, corresponding to C═O stretching vibrations of PTO and imide units, and C═ N bond vibrations, respectively.^[^ ^29^, ^46^ ^]^ Upon discharge from 4.0 to 1.2 V, the intensity of the 1674 cm^−1^ peak decreases, indicating the involvement of carbonyl groups in Li^+^ coordination. Concurrently, the broad peak associated with the C═N bonds also broadens and loses intensity, suggesting its participation in the redox process. It is also worth noting that the 1600–1400 cm^−1^ region includes C═C ring vibrations, which are sensitive to aromaticity changes upon reduction.^[^ ^47^ ^]^ Upon recharging to 4.0 V, the peaks at 1674 and 1593 cm^−1^ largely return to their original positions and intensities, demonstrating the high reversibility of the electrochemical process.

**Figure 6:**
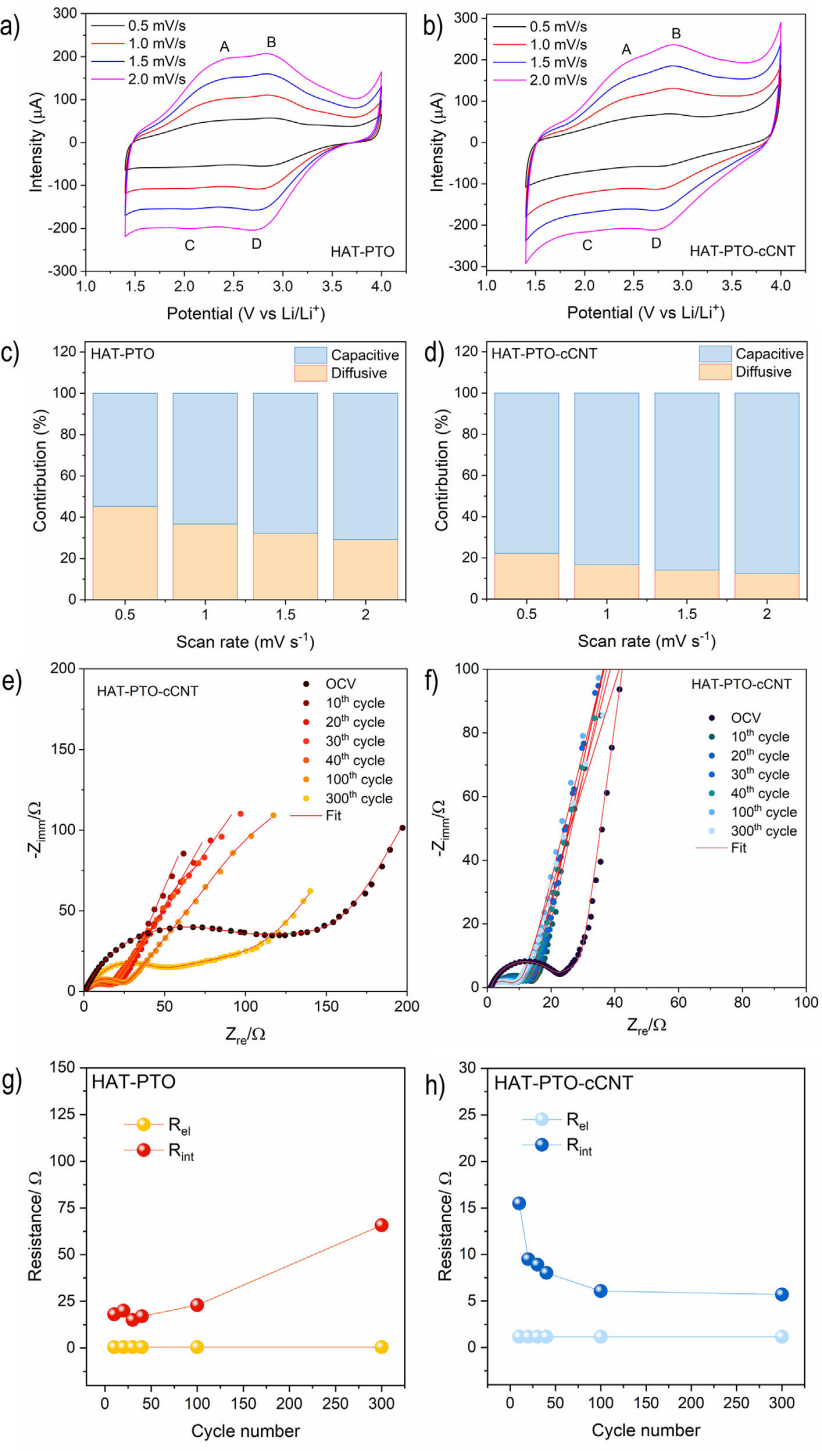
a) CVs of HAT‐PTO at different scan rates. b) CVs of HAT‐PTO‐cCNT at different scan rates. Capacitive‐diffusive contribution (%) calculated for peak C of c) HAT‐PTO, and d) HAT‐PTO‐CNT. Electrochemical impedance spectroscopy was performed at OCV, cycle 10, 20, 30, 40, 100, and 300. Nyquist dispersion of e) HAT‐PTO and f) HAT‐PTO‐cCNT together with the resistance trend obtained by the fitting procedure of g) HAT‐PTO and f) HAT‐PTO‐cCNT.

The lithiation mechanism was further investigated by CV at various scan rates for both HAT‐PTO and HAT‐PTO‐cCNT (**Figure** **6**a,b). The power‐law relationship (*i* = *aν^b^*) provides mechanistic insights, where the exponent *b* indicates the dominant charge storage process: *b* values close to 0.5 correspond to diffusion‐controlled processes, while *b* values close to 1.0 suggest a surface‐controlled, pseudocapacitive behavior.^[^ ^48^ ^]^ The peak currents of the two redox processes were plotted as a function of scan rate, yielding linear correlations with R^2^ values close to 1 (Figure S31a,b, Supporting Information), confirming the validity of the model. The logarithmic plot of the current peaks vs scan rate (Figure S31c,d, Supporting Information) revealed that HAT‐PTO exhibits lower *b* values (0.856, 0.886, 0.815, 0.942 for peaks A, B.C and D, respectively), indicating a greater diffusion‐limited contribution. In contrast, HAT‐PTO‐cCNT displays *b* values near unity (0.95, 0.93, 0.92, 0.95 for peaks A, B, C, and D), highlighting a dominant capacitive contribution facilitated by the improved conductivity of the hybrid.

A quantitative analysis using Dunn's method was conducted to distinguish the capacitive and diffusion‐controlled contributions at each redox peak (A, B, C, D) for both HAT‐PTO and HAT‐PTO‐cCNT across various scan rates. This method assesses the current response at fixed potentials, with linear fitting applied to extract the parameters *k_1_* and *k_2_* (Figure S31, Supporting Information). These values were subsequently used to quantify the capacitive and diffusion‐controlled contributions (Table S5, Supporting Information). As a representative example, the analysis of peak C is shown for both materials in Figure 6c,d, while the results for the remaining peaks are provided in Figures S32 and S33 (Supporting Information). As expected, the capacitive contribution increases with the scan rate for both materials, due to higher current densities and shorter timescales that restrict lithium‐ion diffusion. Specifically, for peak A of HAT‐PTO, the capacitive contribution increases from 63% at 0.5 mV s^−1^ to 77% at 2.0 mV s^−1^ (Figure S32, Supporting Information). Similarly, in HAT‐PTO‐cCNT, it rises from 79% to 89% (Figure S33, Supporting Information). Notably, HAT‐PTO‐cCNT consistently shows a higher capacitive contribution across all redox peaks, in agreement with the enhanced charge transport enabled by carbon nanotubes incorporation.^[^ ^44^, ^49^ ^]^

The diffusion coefficient (D_Li_^+^) of HAT‐PTO was calculated using the Randles‐Ševčík equation (Equation S7, Supporting Information) for each redox peak across the range of scan rates. The average values obtained for each peak were consistent (Figure S34, Supporting Information), resulting in a diffusion coefficient of 4.5 × 10^−9^ cm^2^ s^−1^. This value aligns well with diffusion coefficients previously reported for similar porous organic polymers.^[^ ^30^, ^50^, ^51^ ^]^

To gain deeper insight into the electrochemical behavior and long‐term stability of the materials, electrochemical impedance spectroscopy (EIS) was conducted at selected cycling stages for both pristine HAT‐PTO (Figure 6e) and the HAT‐PTO‐cCNT hybrid (Figure 6f) electrodes. A three‐electrode Swagelok‐type cell was assembled using lithium metal as both counter and reference electrodes, with HAT‐PTO or HAT‐PTO‐cCNT serving as the working electrode. The resulting Nyquist plots exhibit three characteristic regions: i) a high‐frequency intercept along the real axis, ii) a semicircular arc at intermediate frequencies, and iii) a low‐frequency tail, which is typically associated with ion diffusion in the solid phase. The data were interpreted using an equivalent circuit approach (shown in the inset), with fitting carried out using a non‐linear least squares (NLLS) method. The high‐frequency intercept corresponds to the electrolyte's bulk resistance (R_e_). The intermediate‐frequency semicircle reflects interfacial processes, including charge‐transfer resistance (CT) and double‐layer capacitance at the electrode–electrolyte boundary, modeled as a resistor (R_i_) in parallel with a capacitive component (C_i_). The low‐frequency tail is attributed to finite‐length diffusion processes within the electrode, represented using a finite‐space Warburg element (W) with reflective boundary conditions. Initially, the equivalent circuit followed Boukamp notation as R_e_(R_i_C_i_)W. To more accurately describe the non‐ideal capacitive response —linked to surface irregularities and electrode heterogeneity— the ideal capacitor (C) was replaced by a constant phase element (Q) in the final fitting scheme. The corresponding fitted parameters are summarized in Table S6 (Supporting Information).

For both systems, the high‐frequency intercept on the real axis, corresponding to the bulk resistance of the electrolyte (Figure 6g,h), remained low and stable throughout cycling. However, a marked difference was observed in the evolution of the interfacial resistance (R_i_) between the two materials. For the pristine HAT‐PTO electrode, R_i_ remained relatively stable during the first 40 cycles but gradually increased from cycle 40 to cycle 100. Between cycles 100 and 300, R_i_ increased significantly, reaching nearly three times its initial value. This trend suggests the formation of degradation products leading to the growth of a thicker, more passivating cathode electrolyte interphase (CEI) layer, which may impede charge transfer and adversely affect electrode conductivity.

In contrast, the hybrid HAT‐PTO‐cCNT electrode exhibited a markedly different impedance evolution. Between cycles 10 and 20, a noticeable decrease in interfacial resistance was observed, indicating a short activation phase during which the electrode–electrolyte interface becomes progressively optimized for ion and charge transfer. After this initial period, R_i_ remained stable and low up to cycle 300, reflecting a highly conductive and durable interface. This behavior can be attributed to the incorporation of carboxyl‐functionalized carbon nanotubes, which enhance the electrical connectivity and likely contribute to improved stability and reduced resistance.

Overall, these results underline the beneficial role of carbon nanotube integration in enhancing interfacial stability and maintaining a low impedance over prolonged cycling. This improvement accounts for the superior electrochemical performance and cycling stability of the HAT‐PTO‐cCNT hybrid, which exhibited outstanding performance up to 6000 cycles. In contrast, the pristine HAT‐PTO electrode showed lower performance after only 1500 cycles, highlighting the critical importance of conductive additives in the electrode formulation.

**Figure 7:**
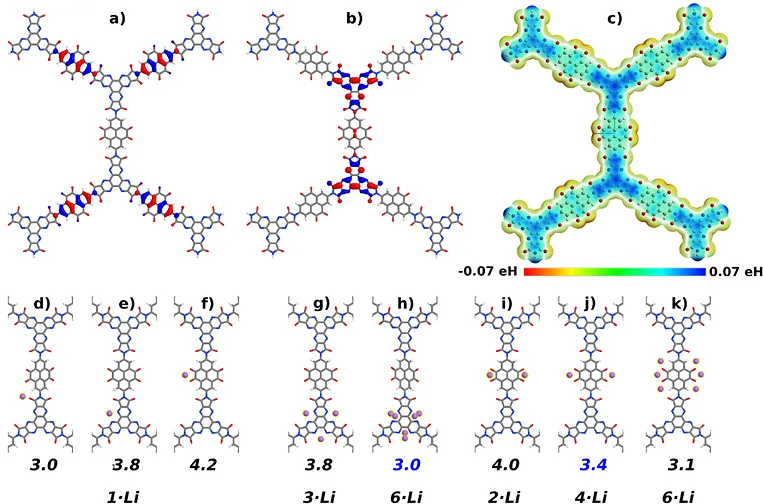
Properties of the HAT‐PTO monolayer molecular analogue and Li adsorption sites computed with DFT. a) HOMO and b) LUMO energy levels of simplified HAT‐PTO model. c) Molecular electrostatic potential. Adsorption energies per atom for 1·Li (d–f), 3·Li (g), and 6·Li (h) at the HAT moiety and 2·Li (i), 4·Li (j), and 6·Li(k) at the PTO moieties. All reported values are in eV unless otherwise stated. In all cases except two (highlighted in blue) the adsorption sites are located primarily on the HAT‐PTO plane.

To gain deeper insight into the lithium storage mechanism of the HAT‐PTO cathode, density functional theory (DFT) calculations at the B3LYP‐6‐31G(d,p) level were performed on a finite molecule representing a HAT‐PTO monolayer (see Experimental Section, Figure S35, Supporting Information). DFT calculations yielded a HOMO–LUMO gap of 2.77 eV. The HOMO (−7.03 eV) and LUMO (−4.26 eV) are predominantly localized on the PTO and HAT moieties, respectively (**Figure** **7**a,b). Notably, the relatively low‐lying LUMO suggests a strong electron‐accepting character. A comprehensive exploration using the M06‐2X/6‐31G(d,p) level of theory was subsequently conducted to predict the most thermodynamically favorable Li adsorption sites. The calculations revealed that the preferred adsorption occurs near the C═O groups of the PTO and imide units, as well as the C═N groups within the pyrazine rings of HAT, consistent with ex situ FT‐IR spectroscopic observations (see above). This finding also aligns with the molecular electrostatic potential map, which shows pronounced negative (Li⁺‐attracting) potential regions near these moieties on the HAT‐PTO plane, in contrast to the positive (Li⁺‐repelling) potential above and below the plane (Figure 7c). The Li insertion energies were found to range from 4.2 eV for a single Li atom adsorbed at the PTO unit to 3.0 eV per atom for six Li atoms inserted above and below the HAT moiety (Figure 7d–k). The energetics of single Li insertion follow the trend PTO > HAT, a tendency that also persists at higher loadings and correlates well with the electrostatic potential distribution. Notably, in HAT‐PTO, the PTO moiety can accommodate not only four Li atoms as previously reported,^[^ ^30^ ^]^ but, due to the presence of the adjacent imide groups, may host up to six Li atoms on the HAT‐PTO plane. This increased capacity is potentially favored by the predicted ABC‐like stacking arrangement and may contribute to the high specific capacity observed for this material.

## 3 Conclusion

In summary, we have synthesized a new polyimide‐linked POP incorporating constructed from redox‐active HAT and PTO building blocks via a straightforward hydrothermal method. The resulting framework (HAT‐PTO) integrates multiple redox‐active C═O and C═N centers, achieving a remarkable theoretical capacity of 484 mAh g^−1^, among the highest reported for redox‐active POPs/COFs. To enhance electronic conductivity and cycling stability, hybrids via in situ growth of **HAT‐PTO** on pristine and carboxyl‐functionalized carbon nanotubes (CNT and cCNT) were prepared. Remarkably, the **HAT‐PTO‐cCNT** hybrid exhibits outstanding electrochemical performance, including a high specific capacity of 397 mAh g^−1^ at C/10, a high‐rate capability of 225 mAh g^−1^ at 20 C, and long‐term cycling stability over 6000 cycles at 2 C with 171 mAh g^−1^ retained. Ex situ FT‐IR and DFT calculations further support the involvement of both HAT and PTO moieties in the redox process, which are responsible for the high capacity of the material. This work demonstrates an effective molecular design strategy and scalable synthesis approach for the development of high‐performance organic cathodes with multiple redox‐active sites, offering a promising pathway toward durable, high‐rate lithium–organic batteries. Current research is focused on optimizing the mass loading of the active material and the cCNT content to maximize the electrochemical performance, as well as exploring this promising organic electrode for other types of energy storage systems, including multivalent batteries.

## 4 Experimental Section

### General Methods and Materials

All the materials including HATCN (hexaazatriphenylenehexacarbonitrile, FluoroChem Germany), concentrated H_2_SO_4_ (Fisher Chemical), sodium hydroxide (Fisher Chemical), sodium hydrogen carbonate (Fisher Chemical), glacial acetic acid (Fisher Chemical), sodium nitrite (Fisher Scientific), trifluroacteic acid (TCI), sulfuric acid (95%, Fisher Chemical), hydrochloric acid (37%, Fisher Chemical), dimethyl sulfoxide (99.9%, anhydrous, Fisher Scientific), 2,7‐Diaminopyrene‐4,5,9,10‐tetraone, 98% (ChemExtension and BLD pharma), carbon nanotube, multi‐walled carboxylic acid functionalized (>90% carbon basis, D × L 9.5 nm × 1.5 µm, Sigma Aldrich), carbon nanotube, multi‐walled (50–90 nm diameter, >95% carbon basis, Sigma Aldrich) were used as purchased without any purification. Ultrapure water (≤15.0 MΩ) produced by a Millipore Milli‐Q gradient system from Merck was used as the solvent for hydrothermal reactions.

### ^1^H and ^13^C Liquid‐State NMR

NMR spectra were recorded using a Varian Mercury 300 MHz NMR spectrometer. Tetramethylsilane (TMS) was used as an internal reference. Chemical shifts (*δ)* were quoted in ppm from TMS and the coupling constants (*J*) in Hz.

### ^13^C Solid‐State CP‐MAS NMR

The spectra were recorded on a 9.4 T Bruker Avance III 400 spectrometer using a 4 mm Bruker magic‐angle spinning (MAS) probe. Chemical shifts were quoted in ppm from TMS using solid adamantane as secondary reference.

### Powder X‐Ray Diffraction (PXRD)

Data were collected at room temperature with a Bragg–Brentano geometry on a Rigaku MiniFlex X‐ray diffractometer (40 kV, 40 mA, θ/θ configuration). The diffractometer was equipped with a sealed Cu X‐ray tube (λ_CuKα1_ = 1.5406 Å). Diffractograms of the powder were obtained in the angular range of 2°–40° θ with a step size of 0.02° (2θ) at 1 s per step. The samples were settled on a Si (511) oriented crystal base to avoid background noise caused by a traditional glass support.

### Fourier Transform Infrared

FTIR spectra were recorded using powdered samples in a PerkinElmer's Spectrum spectrometer in the 4000–400 cm^−1^ range.

### Thermogravimetric Analysis

TGA was carried out with a Thermal Analysis System (TGA/DSC 3+) of Mettler Toledo with a N_2_ flow rate of 19 mL min^−1^ and a heating rate of 5 °C min^−1^.

### Gas Sorption Measurements

N_2_ adsorption‐desorption isotherms were measured ex‐situ on a Micromeritics 3Flex apparatus at 77 K. Before measurements, the samples were outgassed at 393 K and 10^−6^ Torr overnight using Smart VacPrep (Micrometrics) equipment. Pore size distributions were obtained using the non‐local density functional theory (NLDFT) method.

### Mass Spectrometry

High‐resolution mass spectrometry was conducted on a Bruker microTOF spectrometer using APCI‐FIA.

### Elemental Analysis

Experiment**s** were performed on a Flash Smart equipment (Thermoscientific) where the samples were burned at a temperature of 1020 °C with a stream of extra pure He and an injection of O_2_. The analysis time was 720 s, and the software program was EAGER SMART.

### Electron Microscopy

Scanning electron microscopy images were recorded using a FESEM equipment (ultra plus, Zeiss, Germany). High‐resolution transmission electron microscopy analysis was performed on JEOL JEM F200CF‐HR Microscope (200 KeV) with a cold field emission electron gun and HR objective lens. Energy dispersive spectroscopy (EDS) mapping images were acquired using JEOL JED‐2300T Microanalysis System with a 100mm^2^ active area Centurio SDD Detector, One View Camera with CMOS sensor, and 4K × 4K resolution.

### UV–Vis Spectroscopy

UV–vis spectra ranging from 200 to 850 nm were recorded using an Agilent Cary 3500 Multicell UV–vis Spectrophotometer. The measurements were performed using a 1 cm quartz cell.

### Gas Chromatography–Mass Spectrometry (GC–MS)

GC–MS analyses were carried out using an Agilent Technologies 6890N Network GC system coupled with an Agilent Technologies 5973 Inert Mass Selective Detector (MSD). Samples were diluted in dichloromethane prior to injection.

### Raman Spectroscopy

Raman spectra were recorded using a InVia Reflex Renishaw Raman equipment (785 nm) at room temperature.

### Electrode Preparation

The working electrodes were prepared by mixing **HAT‐PTO** with conductive carbon black (Super C65) and polyvinylidene fluoride (PVDF) binder in a mass ratio of 60:30:10. N‐Methyl‐2‐pyrrolidone was added during the mixing process to form a homogeneous slurry. The slurry was then coated onto an aluminum foil substrate and dried at 60 °C in a vacuum for 12 h. For **HAT‐PTO‐CNT** or **HAT‐PTO‐cCNT**, conductive carbon black, and PVDF binder mixed in a mass ratio of 80:10:10. The as‐obtained films were punched into disks with a diameter of 12 mm.

### Electrochemical Characterization

All electrochemical performances were measured using CR2032‐type coin cells and three‐electrode Swagelok‐type cell, assembled in an argon‐filled glove box (MBraun; H_2_O and O_2_ levels <1 ppm). Lithium metal was used as both counter and reference electrodes and **HAT‐PTO, HAT‐PTO‐CNT** or **HAT‐PTO‐cCNT** hybrids as working electrodes, respectively. 1 M LiTFSI dissolved in a 1:1 (v/v) mixture of DOL and DME was used as electrolyte. A Li disk was used as the anode with Celgard 2325 membrane as the separator. Galvanostatic charge/discharge test was conducted in the voltage range of 1.2–4.0 V (vs Li/Li^+^) on a LAND CT2001A instrument (Wuhan, China). Cyclic voltammetry (CV) at different scan rates (0.5, 1.0, 1.5, and 2.0 mV s^−1^ with a window voltage of 1.4–4.0 V vs Li/Li^+^) and Electrochemical Impedance Spectroscopy (EIS) were performed on VIONIC potentiostat and Intello software (Metrohm). Impedance spectra were recorded at open circuit voltage (OCV) and after the 10th, 20th, 30th, 40th, 100th, and 300th cycles to monitor variations in resistance and assess the long‐term stability of the electrode–electrolyte interphase. The resulting Nyquist plots were modelled using equivalent circuit method and fitted through a nonlinear least squares (NLLS) approach via the RelaxIS 3 software (RHD Instruments, Darmstadt, Germany). The equivalent circuit employed, denoted in Boukamp notation as Re(RiCi)W, was modified by replacing the ideal capacitor (C) with a constant phase element (Q) to more accurately capture the non‐ideal electrochemical behavior attributed to surface roughness and morphological heterogeneity at the interface.^[^ ^52^, ^53^ ^]^ All the electrochemical tests were conducted at 25 °C. Linear sweep voltammetry (LSV) measurements were carried out on an Autolab Metrohm VIONIC potentiostat, starting from the open‐circuit voltage (OCV) up to 5.0 V, with a scan rate of 0.1 mV s^−1^. Chronoamperometric measurements were carried out on a BioLogic MVP‐300 potentiostat for a duration of 3600 s using the HAT‐PTO‐cCNT electrode. The experiments were performed at four different potentials: 3.0, 3.5, and 3.9 V, and slightly above the cycling potential, at 4.05 V.

### Ex Situ Infrared Spectroscopy

Experiments were performed on HAT‐PTO electrodes prepared following the previously described procedure, followed by the described cycling protocol. Electrodes were harvested from half‐cells in 15th charge/discharge cycle, washed three times with 2 mL of DME, and dried overnight. Infrared measurements were performed on Bruker Alpha II spectrometer on KBr pellets containing ≈1% of active material. Spectra were collected over the 4000–500 cm^−1^ range, with 48 scans at a resolution of 4 cm^−1^. All electrode handling and measurements were carried out inside an Ar‐filled glovebox (H_2_O and O_2_ < 1 ppm) to prevent material degradation.

### Synthesis of HAT‐PTO

HAT‐6COOH (49.8 mg, 0.10 mmol), PTO‐2NH_2_ (43.8 mg, 0.15 mmol), and 10 mL of deionized water were added to a 25 mL PTFE‐lined stainless‐steel autoclave. The reaction mixture was sonicated for 20 min to ensure homogenization, sealed, and heated at 200 °C for 48 h. The resulting solid was collected by filtration and washed sequentially with DMSO, water, and acetone. The product was dried under vacuum at 80 °C to yield **HAT‐PTO** as a black powder (60 mg, 68% yield).

### Synthesis of HAT‐PTO‐CNT and HAT‐PTO‐cCNT

CNT hybrids of **HAT‐PTO** were synthesized following a similar procedure. HAT‐6COOH (49.8 mg, 0.10 mmol), PTO‐4NH_2_ (43.8 mg, 0.15 mmol), carbon nanotubes (cCNT or CNT) (45 mg or 22 mg corresponding to 43% and 27% w/w in the composite, respectively) and 10 mL of deionized water were added to a 25 mL PTFE‐lined stainless‐steel autoclave. The mixture was sonicated for 20 min to ensure homogenization, sealed, and heated at 200 °C for 48 h. After cooling, the solids were collected by filtration and washed successively with DMSO, deionized water, and acetone. The products were dried under vacuum at 80 °C to yield **HAT‐PTO‐cCNT** and **HAT‐PTO‐CNT** as black powders with yields of 90% and 89%, respectively. The mass content of **HAT‐PTO** in the hybrids was controlled as 46% (or 59% for **HAT‐PTO‐cCNT‐lc** and **HAT‐PTO‐CNT‐lc**) based on the amount of CNT or cCNT used and the yield of **HAT‐PTO**.

### Computational Details

3D periodic DFT calculations were performed with the QuantumATK simulation package^[^ ^54^, ^55^ ^]^ with a unit cell containing one (eclipsed AA stacking), two (PD AA and AB stackings) and three (ABC stacking) molecular layers, to accommodate the different interlayer registries. Periodic calculations were initially performed at the LDA level and subsequently refined at the PBE level augmented with Grimme's D2 dispersion empirical correction.^[^ ^56^ ^]^ The medium numerical basis set was used in all cases. Molecular calculations were performed with the B3LYP and M06‐2X Hamiltonians using the 6–31G(d,p) basis set with Gaussian16 C.^[^ ^57^ ^]^ For this, a molecular fragment, was cut out from a extended DFT flat model, and saturated with hydrogens yielding a C_188_H_28_N_54_O_5_ molecule with 326 atoms. This molecule was optimized by enforcing planar symmetry with the hybrid B3LYP Hamiltonian. Then, several representative lithium adsorption sites adding up to more than 70 different separate computations were explored. Initially, the Li atom positions were optimized with an eXtended Tight Binding (xTB) Hamiltonian and later refined with DFT (M06‐2X) on a frozen substrate. The so‐obtained lithium positions were then combined and optimized together with the central region of the COF model with the same Hamiltonian, while the four outer regions, representing the embedding on the extended 2D HAT‐PTO monolayer, were constrained, Figure S35 (Supporting Information). The adsorption energies were then computed and energy‐ranked to quantify the thermodynamical probability of different Li insertions for HAT‐PTO.

## Conflict of Interest

The authors declare no conflict of interest.

## Supporting information



Supporting Information

## Data Availability

The data that support the findings of this study are available in the supplementary material of this article.

## References

[1] J. B. Goodenough , Nat. Electron. 2018, 1, 204.

[2] M. Li , J. Lu , Z. Chen , K. Amine , Adv. Mater. 2018, 30, 1800561. 10.1002/adma.201800561 29904941

[3] J. W. Choi , D. Aurbach , Nat. Rev. Mater. 2016, 1, 16013.

[4] A. Manthiram , ACS Cent. Sci. 2017, 3, 1063. 29104922 10.1021/acscentsci.7b00288 PMC5658750

[5] A. Manthiram , Nat. Commun. 2020, 11, 1550. 32214093 10.1038/s41467-020-15355-0 PMC7096394

[6] M. Armand , J.‐M. Tarascon , Nature 2008, 451, 652. 18256660 10.1038/451652a

[7] T. B. Schon , B. T. McAllister , P. F. Li , D. S. Seferos , Chem. Soc. Rev. 2016, 45, 6345. 27273252 10.1039/c6cs00173d

[8] Y. Lu , Q. Zhang , L. Li , Z. Niu , J. Chen , Chem 2018, 4, 2786.

[9] P. Poizot , J. Gaubicher , S. Renault , L. Dubois , Y. Liang , Y. Yao , Chem. Rev. 2020, 120, 6490. 32207919 10.1021/acs.chemrev.9b00482

[10] Y. Lu , J. Chen , Nat. Rev. Chem. 2020, 4, 127. 37128020 10.1038/s41570-020-0160-9

[11] B. Esser , F. Dolhem , M. Becuwe , P. Poizot , A. Vlad , D. Brandell , J. Power. Sources 2021, 482, 228814.

[12] J. Bitenc , K. Pirnat , O. Lužanin , R. Dominko , Chem. Mater. 2024, 36, 1025. 38370280 10.1021/acs.chemmater.3c02408 PMC10870817

[13] R. Wessling , P. Penert , B. Esser , Adv. Energy Mater. 2025, 15, 2500150.

[14] J. Kim , Y. Kim , J. Yoo , G. Kwon , Y. Ko , K. Kang , Nat. Rev. Mater. 2022, 8, 54.

[15] S. Muench , A. Wild , C. Friebe , B. Häupler , T. Janoschka , U. S. Schubert , Chem. Rev. 2016, 116, 9438. 27479607 10.1021/acs.chemrev.6b00070

[16] T. Janoschka , M. D. Hager , U. S. Schubert , Adv. Mater. 2012, 24, 6397. 23238940 10.1002/adma.201203119

[17] D. Luo , M. Li , Q. Ma , G. Wen , H. Dou , B. Ren , Y. Liu , X. Wang , L. Shui , Z. Chen , Chem. Soc. Rev. 2022, 51, 2917. 35285470 10.1039/d1cs01014j

[18] R. Dantas , C. Ribeiro , M. Souto , Chem. Commun. 2024, 60, 138. 10.1039/d3cc04322c 38051115

[19] S. Haldar , A. Schneemann , S. Kaskel , J. Am. Chem. Soc. 2023, 145, 13494. 37307595 10.1021/jacs.3c01131 PMC10311462

[20] A. M. Evans , M. J. Strauss , A. R. Corcos , Z. Hirani , W. Ji , L. S. Hamachi , X. Aguilar‐Enriquez , A. D. Chavez , B. J. Smith , W. R. Dichtel , Chem. Rev. 2022, 122, 442. 34852192 10.1021/acs.chemrev.0c01184

[21] M. G. Mohamed , A. F. M. EL‐Mahdy , M. G. Kotp , S.‐W. Kuo , Mater. Adv 2022, 3, 707.

[22] K. T. Tan , S. Ghosh , Z. Wang , F. Wen , D. Rodríguez‐San‐Miguel , J. Feng , N. Huang , W. Wang , F. Zamora , X. Feng , A. Thomas , D. Jiang , Nat. Rev. Methods Primers 2023, 3, 1.

[23] M. Souto , K. Strutyński , M. Melle‐Franco , J. Rocha , Chem. ‐ Eur. J. 2020, 26, 10912. 32293769 10.1002/chem.202001211

[24] C. Peng , G.‐H. Ning , J. Su , G. Zhong , W. Tang , B. Tian , C. Su , D. Yu , L. Zu , J. Yang , M.‐F. Ng , Y.‐S. Hu , Y. Yang , M. Armand , K. P. Loh , Nat. Energy 2017, 2, 17074.

[25] M. Wu , Y. Zhao , H. Zhang , J. Zhu , Y. Ma , C. Li , Y. Zhang , Y. Chen , Nano Res. 2022, 15, 9779.

[26] R. R. Kapaev , I. S. Zhidkov , E. Z. Kurmaev , K. J. Stevenson , P. A. Troshin , J. Mater. Chem. A 2019, 7, 22596.

[27] S. Xu , G. Wang , B. P. Biswal , M. Addicoat , S. Paasch , W. Sheng , X. Zhuang , E. Brunner , T. Heine , R. Berger , X. Feng , Angew. Chem., Int. Ed. 2019, 58, 849. 10.1002/anie.201812685 30461145

[28] X. Yang , L. Gong , X. Liu , P. Zhang , B. Li , D. Qi , K. Wang , F. He , J. Jiang , Angew. Chem., Int. Ed. 2022, 61, 202207043. 10.1002/anie.202207043 35638157

[29] P. Xu , F. Gao , D. Liu , Adv. Mater. Interfaces 2023, 10, 2300464.

[30] Q. Bai , J. Huang , K. Tang , Y. Zhu , D. Wu , Adv. Mater. 2025, 37, 2416661. 10.1002/adma.202416661 39981813

[31] X. Wu , S. Zhang , X. Xu , F. Wen , H. Wang , H. Chen , X. Fan , N. Huang , Angew. Chem., Int. Ed. 2024, 63, 202319355. 10.1002/anie.202319355 38227349

[32] Z. Sun , Z. Li , J. Peng , X. Yan , H. Shang , Y. Jin , Q. Zhao , C. Li , S. Lyu , C. Chen , J.‐B. Baek , Energy Environ. Sci. 2025, 18, 5159.

[33] T. Nokami , T. Matsuo , Y. Inatomi , N. Hojo , T. Tsukagoshi , H. Yoshizawa , A. Shimizu , H. Kuramoto , K. Komae , H. Tsuyama , J. Yoshida , J. Am. Chem. Soc. 2012, 134, 19694. 23130634 10.1021/ja306663g

[34] X. Luo , W. Li , H. Liang , H. Zhang , K. Du , X. Wang , X. Liu , J. Zhang , X. Wu , Angew. Chem., Int. Ed. 2022, 61, 202117661. 10.1002/anie.202117661 35034424

[35] H. Gao , A. R. Neale , Q. Zhu , M. Bahri , X. Wang , H. Yang , Y. Xu , R. Clowes , N. D. Browning , M. A. Little , L. J. Hardwick , A. I. Cooper , J. Am. Chem. Soc. 2022, 144, 9434. 35588159 10.1021/jacs.2c02196 PMC9164232

[36] C. Yao , Z. Wu , J. Xie , F. Yu , W. Guo , Z. J. Xu , D. Li , S. Zhang , Q. Zhang , ChemSusChem 2020, 13, 2457. 31782976 10.1002/cssc.201903007

[37] X. Liu , Y. Jin , H. Wang , X. Yang , P. Zhang , K. Wang , J. Jiang , Adv. Mater. 2022, 34, 2203605. 10.1002/adma.202203605 35905464

[38] C. Li , A. Yu , W. Zhao , G. Long , Q. Zhang , S. Mei , C. Yao , Angew. Chem., Int. Ed. 2024, 63, 202409421. 10.1002/anie.202409421 39136328

[39] H. Duan , K. Li , M. Xie , J.‐M. Chen , H.‐G. Zhou , X. Wu , G.‐H. Ning , A. I. Cooper , D. Li , J. Am. Chem. Soc. 2021, 143, 19446. 34731564 10.1021/jacs.1c08675

[40] J. Li , J. Zhang , Y. Hou , J. Suo , J. Liu , H. Li , S. Qiu , V. Valtchev , Q. Fang , X. Liu , Angew. Chem., Int. Ed. 2024, 63, 202412452. 10.1002/anie.202412452 39343741

[41] Y. Yang , S. Wang , Y. Duan , T. Wang , F. Wang , H. Zhu , Z. Wang , K. Zhang , P. Cheng , Z. Zhang , Angew. Chem., Int. Ed. 2025, 64, 202418394. 10.1002/anie.202418394 39585117

[42] W. Zhao , X. Wang , M. Bahri , Q. Zhu , B. Li , K. Wu , Z. Wang , H. Li , X. Shi , D. Shi , C. Ji , N. D. Browning , J. Sun , J. Wang , D. Zhao , J. Am. Chem. Soc. 2025, 147, 16319. 40304088 10.1021/jacs.5c01990

[43] T. Kim , S. H. Joo , J. Gong , S. Choi , J. H. Min , Y. Kim , G. Lee , E. Lee , S. Park , S. K. Kwak , H. Lee , B. Kim , Angew. Chem., Int. Ed. 2022, 134, 202113780. 10.1002/anie.202113780 34708501

[44] H. Gao , Q. Zhu , A. R. Neale , M. Bahri , X. Wang , H. Yang , L. Liu , R. Clowes , N. D. Browning , R. S. Sprick , M. A. Little , L. J. Hardwick , A. I. Cooper , Adv. Energy Mater. 2021, 11, 2101880.

[45] S. Zheng , L. Miao , T. Sun , L. Li , T. Ma , J. Bao , Z. Tao , J. Chen , J. Mater. Chem. A 2021, 9, 2700.

[46] Q. Fang , Z. Zhuang , S. Gu , R. B. Kaspar , J. Zheng , J. Wang , S. Qiu , Y. Yan , Nat. Commun. 2014, 5, 4503. 25054211 10.1038/ncomms5503

[47] S. Menart , O. Lužanin , K. Pirnat , D. Pahovnik , J. Moškon , R. Dominko , ACS Appl. Mater. Interfaces 2024, 16, 16029. 38511931 10.1021/acsami.3c16038 PMC10995900

[48] B. E. Conway , Electrochemical Supercapacitors, Springer USA, Boston, MA 1999.

[49] D. Yan , L. Song , F. Kang , X. Mo , Y. Lv , J. Sun , H. Tang , X. Zhou , Q. Zhang , Angew. Chem., Int. Ed. 2025, 64, 202422851. 10.1002/anie.202422851 39731475

[50] E. Vitaku , C. N. Gannett , K. L. Carpenter , L. Shen , H. D. Abruña , W. R. Dichtel , J. Am. Chem. Soc. 2020, 142, 16. 31820958 10.1021/jacs.9b08147

[51] K. Huang , J. Li , Q. Bai , X. Wu , Y. Zhu , CCS Chem. 2025.

[52] B. Boukamp , Solid State Ion 1986, 18 , 136.

[53] B. Boukamp , Solid State Ion 1986, 20, 31.

[54] S. Smidstrup , T. Markussen , P. Vancraeyveld , J. Wellendorff , J. Schneider , T. Gunst , B. Verstichel , D. Stradi , P. A. Khomyakov , U. G. Vej‐Hansen , J. Phys.: Condens. Matter 2020, 32, 015901. 31470430 10.1088/1361-648X/ab4007

[55] Synopsys , QuantumATK: Atomistic Simulation Software, https://www.synopsys.com/silicon/quantumatk.html.

[56] J. A. S. Grimme , S. Ehrlich , H. Krieg , J. Chem. Phys. 2010, 132, 154104. 20423165 10.1063/1.3382344

[57] M. J. Frisch , G. W. Trucks , H. B. Schlegel , G. E. Scuseria , M. A. Robb , J. R. Cheeseman , G. Scalmani , V. Barone , G. A. Petersson , H. Nakatsuji , X. Li , M. Caricato , A. V. Marenich , J. Bloino , B. G. Janesko , R. Gomperts , B. Mennucci , H. P. Hratchian , J. V. Ortiz , A. F. Izmaylov , J. L. Sonnenberg , D. Williams‐Young , F. Ding , F. Lipparini , F. Egidi , J. Goings , B. Peng , A. Petrone , T. Henderson , D. Ranasinghe , et al., Gaussian 16, Revision C.01, Gaussian, Inc., Wallingford CT, 2019.

