# Supplementary material for: Polyimide‐Linked Hexaazatriphenylene‐Based Porous Organic Polymer with Multiple Redox‐Active Sites as a High‐Capacity Organic Cathode for Lithium‐Ion Batteries
Source: Adv Mater. 2025 Oct 16;38(51):e12950. doi: 10.1002/adma.202512950 (PMC13568856; doi:10.1002/adma.202512950)
Supplement: Supplementary file 1 [file ADMA-38-e12950-s001.pdf]

Supporting Information

Polyimide-linked Hexaazatriphenylene-based Porous Organic Polymer with Multiple Redox-Active Sites as a High-Capacity Organic Cathode for Lithium-Ion Batteries

Arindam Mal, Jonathan Caroni, Asia Patriarchi, Olivera Luzanin, Rafael Ramos, Jan Bitenc, Manuel Melle-Franco* and Manuel Souto**

A. Mal, J. Caroni, R. Ramos, M. Souto
CiQUS, Centro Singular de Investigación en Química Bioloxica e Materiais Moleculares, Departamento de Química-Física, Universidade de Santiago de Compostela, 15782 Santiago de Compostela, Spain
E-mail: manuel.souto.salom@usc.es

A. Patriarchi
Chemistry Division, School of Science and Technology, University of Camerino, Via Madonna delle Carceri-ChIP, 62032 Camerino, MC, Italy

O. Luzanin, J. Bitenc
National Institute of Chemistry, Hajdrihova 19, 1000, Ljubljana, Slovenia

M. Melle-Franco, M. Souto
Department of Chemistry, CICECO-Aveiro Institute of Materials, University of Aveiro, 3810-393 Aveiro, Portugal

M. Souto
Oportunius, Galician Innovation Agency (GAIN), 15702 Santiago de Compostela, Spain

Contents

1. **General methods and materials** S3
2. **Synthesis and characterization of HAT-PTO and hybrids** S4
3. **Electrical conductivity measurements** S14
4. **Calculation of theoretical capacity of HAT-PTO** S16
5. **Electrochemical measurements** S17
6. ***Ex situ* infrared spectroscopy** S26
7. **Mechanistic studies** S27
8. **Computational details** S34
9. **References** S35

1. General methods and materials

All the **materials** including HATCN (hexaazatriphenylenehexacarbonitrile, FluoroChem Germany), concentrated H_2SO_4 (Fisher Chemical), sodium hydroxide (Fisher Chemical), sodium hydrogen carbonate (Fisher Chemical), glacial acetic acid (Fisher Chemical), sodium nitrite (Fisher Scientific), trifluroacteic acid (TCI), sulfuric acid (95%, Fisher Chemical), hydrochloric acid (37%, Fisher Chemical), dimethyl sulfoxide (99.9%, anhydrous, Fisher Scientific), 2,7-Diaminopyrene-4,5,9,10-tetraone, 98% (ChemExtension and BLD pharma), carbon nanotube, multi-walled carboxylic acid functionalized (>90% carbon basis, D × L 9.5 nm × 1.5 µm, Sigma Aldrich), carbon nanotube, multi-walled (50-90 nm diameter, >95% carbon basis, Sigma Aldrich) were used as purchased without any purification. Ultrapure water (≤15.0 MΩ) produced by a Millipore Milli-Q gradient system from Merck was used as the solvent for hydrothermal reactions.

^{1}H and ^{13}C liquid-state NMR spectra were recorded using a Varian Mercury 300 MHz NMR spectrometer. Tetramethylsilane (TMS) was used as an internal reference. Chemical shifts (δ) are quoted in ppm from TMS and the coupling constants (J) in Hz.

^{13}C solid-state CP-MAS NMR: The spectra were recorded on a 9.4 T Bruker Avance III 400 spectrometer using a 4 mm Bruker magic-angle spinning (MAS) probe. Chemical shifts are quoted in ppm from TMS using solid adamantane as secondary references.

Powder X-ray diffraction (PXRD) data were collected at room temperature with a Bragg-Brentano geometry on a Rigaku MiniFlex X-ray diffractometer (40 kV, 40 mA, θ/θ configuration). The diffractometer was equipped with a sealed Cu X-ray tube ($\lambda_{CuK\alpha1}$ = 1.5406 Å). Diffractograms of the powder were obtained in the angular range of 2.0º to 40.0º θ with a step size of 0.02° (2θ) at 1 s per step. The samples were settled on a Si (511) oriented crystal base to avoid background noise caused by a traditional glass support.

Fourier transform infrared (FTIR) spectra were recorded using powdered samples in a PerkinElmer's Spectrum spectrometer in the 4000 – 400 cm^{-1} range.

Thermogravimetric analysis (TGA) was carried out with a Thermal Analysis System (TGA/DSC 3+) of Mettler Toledo with a N_2 flow rate of 19 mL/min and a heating rate of 5 ◦C/min.

Gas sorption measurements: N_2 adsorption-desorption isotherms were measured ex-situ on a Micromeritics 3Flex apparatus at 77 K. Before measurements, the samples were outgassed at 393 K and 10^{-6} Torr overnight using Smart VacPrep (Micrometrics) equipment. Pore size distributions were obtained using the non-local density functional theory (NLDFT) method.

Mass spectrometry: High-resolution mass spectrometry was conducted on a Bruker microTOF spectrometer using APCI-FIA.

Elemental analysis: Experiments were performed on a Flash Smart equipment (Thermoscientific) where the samples were burned at a temperature of 1020 ºC with a stream of extra pure He and an injection of O_2. The analysis time was 720 seconds, and the software program is EAGER SMART.

Electron microscopy: Scanning electron microscopy images were recorded using a FESEM equipment (ultra plus, Zeiss, Germany). High-resolution transmission electron microscopy analysis was performed on JEOL JEM F200CF-HR Microscope (200KeV) with a cold field emission electron gun and HR objective lens. Energy dispersive spectroscopy (EDS) mapping images were acquired using JEOL JED-2300T Microanalysis System with a 100mm² active area Centurio SDD Detector, One View Camera with CMOS sensor and 4K × 4K resolution.

2. Synthesis and characterization of HAT-PTO and hybrids

Synthesis of hexaazatriphenylene hexacarboxylic acid (HAT-6COOH). HAT-6COOH was synthesized by adapting a reported procedure (Scheme S1).[S1]

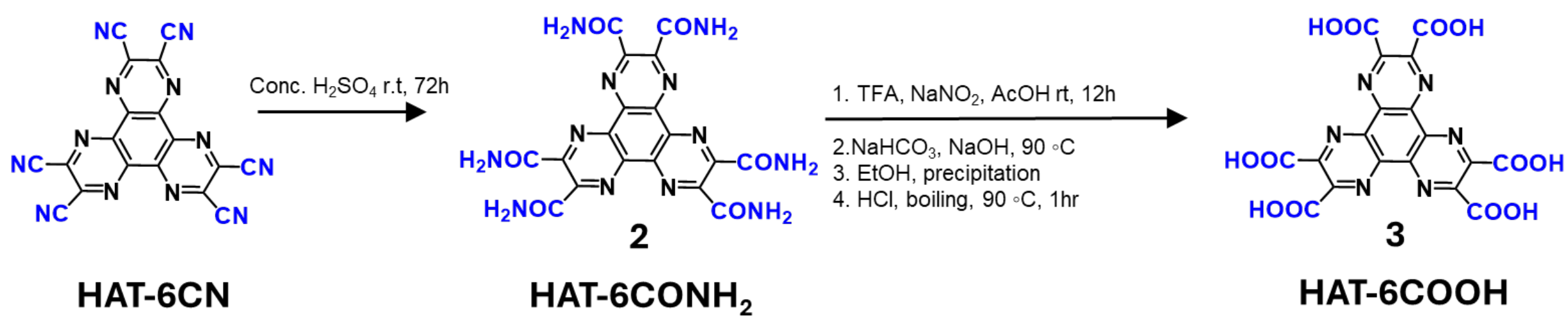

Scheme S1. Synthesis of **HAT-6COOH**.

Synthesis of hexaazatriphenylenehexacarboxamide (2, HAT-6CONH$_2$). HAT-CN (0.5 g, 1.3 mmol) and concentrated H_2SO_4 (10.5 mL) were added to a 100 mL round-bottomed flask and stirred at room temperature for 72h. The resulting red-orange solution was slowly poured into ice-cooled deionized H_2O (50 mL) under stirring, yielding a yellow suspension. The solid was collected by filtration, washed sequentially with deionized H_2O (3 × 10 mL) and acetone (3 × 10 mL), and dried under vacuum to afford **HAT-6CONH$_2$** as a yellow solid (0.612 g, yield: 95%). ^{1}H NMR (300 MHz, DMSO-d$_6$): δ (ppm) = 8.44 (s, 1H), 8.00 (s, 1H). ^{13}C NMR (300 MHz, DMSO-d$_6$) δ (ppm) = 166.61 (s, carboxyl carbons), 148.79 (s, peripheral Ar carbons), 140.89 (s, internal Ar carbons).

Synthesis of hexaazatriphenylenehexacarboxylic acid (3, HAT-6COOH). The as-synthesized compound **2** (**HAT-6CONH$_2$**) (0.5 g) was placed in a 250 mL round-bottom flask and trifluoroacetic acid (15 mL, 0.2 mmol) was added. The mixture was stirred for 15 minutes while maintaining the temperature below 25 ºC using an ice bath. Sodium nitrate ($NaNO_2$, 0.7 g, 10.1 mmol) was then added over 15 minutes. Acetic acid (15 mL) was added subsequently, and the reaction mixture was stirred at room temperature for 12 h before being poured into water and filtered. The crude product was dissolved in sodium bicarbonate solution (4.20 g, 50 mL H_2O) and treated with sodium hydroxide solution (2 g, 50 mL H_2O). This mixture was heated to 90 ºC and maintained at reflux. After cooling to room temperature, ethanol (60 mL) was added, causing immediate precipitation. The precipitate was filtered, washed with 50 % aqueous ethanol (40 mL) and dried under vacuum. The resulting solid was suspended in water (100 mL), heated to 50 ◦C, and acidified with concentrated HCl (50 mL). The mixture was heated at 90 ºC for 1 h, then filtered and washed with 10% HCl (5 × 10 mL), and deionized water (5 × 10 mL), followed by vacuum drying to yield **3** (**HAT-6COOH**) as a bright yellow solid (0.268g, yield: 53.6%). ^{13}C NMR (300 MHz, DMSO-d$_6$) δ (ppm) = 166.19 (s, carboxyl carbons), 147.06 (s, peripheral Ar carbons), 142.18 (s, internal Ar carbons). APCI-MS (m/z): [M$^+$] calc. for $C_{18}H_6N_6O_{12}$, 498.000; found, 500.327.

Synthesis of HAT-PTO

HAT-6COOH (49.8 mg, 0.10 mmol), PTO-2NH$_2$ (43.8 mg, 0.15 mmol), and 10 mL of deionized water were added to a 25 mL PTFE-lined stainless-steel autoclave. The reaction mixture was sonicated for 20 minutes to ensure homogenization, sealed, and heated at 200 ºC for 48 hours. The resulting solid was collected by filtration and washed sequentially with DMSO, water, and acetone. The product was dried under vacuum at 80 ºC to yield **HAT-PTO** as a black powder (60 mg, 68 % yield).

Synthesis of HAT-PTO-CNT and **HAT-PTO-cCNT hybrids**

CNT hybrids of **HAT-PTO** were synthesized following a similar procedure. HAT-6COOH (49.8 mg, 0.10 mmol), PTO-4NH$_2$ (43.8 mg, 0.15 mmol), carbon nanotubes (cCNT or CNT) (45 mg or 22 mg corresponding to 43% and 27% w/w in the composite, respectively) and 10 mL of deionized water were added to a 25 mL PTFE-lined stainless-steel autoclave. The mixture was sonicated for 20 minutes to ensure homogenization, sealed, and heated at 200 ºC for 48 hours. After cooling, the solids were collected by filtration and washed successively with DMSO, deionized water, and acetone. The products were dried under vacuum at 80 °C to yield **HAT-PTO-cCNT** and **HAT-PTO-CNT** as black powders with yields of 90% and 89%, respectively. The mass content of **HAT-PTO** in the hybrids was controlled as 46% (or 59% for **HAT-PTO-cCNT-lc** and **HAT-PTO-CNT-lc**) based on the amount of CNT or cCNT used and the yield of **HAT-PTO**.

Table S1. Elemental analysis data of **HAT-PTO**.

	C	H	N	O
Theoretical (wt. %) $C_{42}H_6N_4O_{12}$	60.88	0.73	15.21	23.17
Experimental CHN*	57.35	2.38	13.41	24.16
SEM-EDX	63.9	-	13.84	17.72

*The sample´s oxygen (O) content was calculated by deducting the contents of C, H and N from 100%.

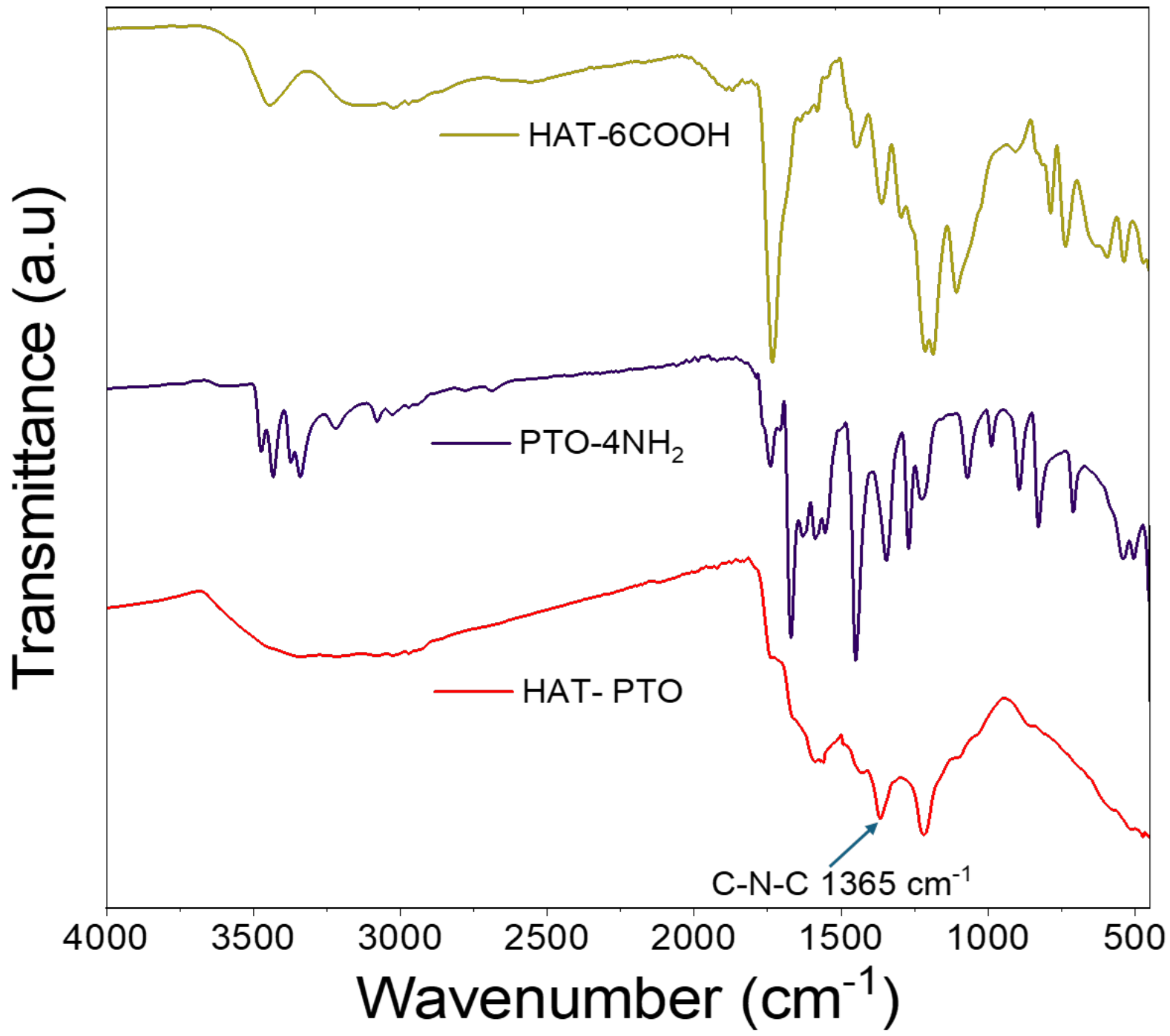

Figure S1. FTIR spectra of HAT-6COOH, PTO-4NH_2, and **HAT-PTO**.

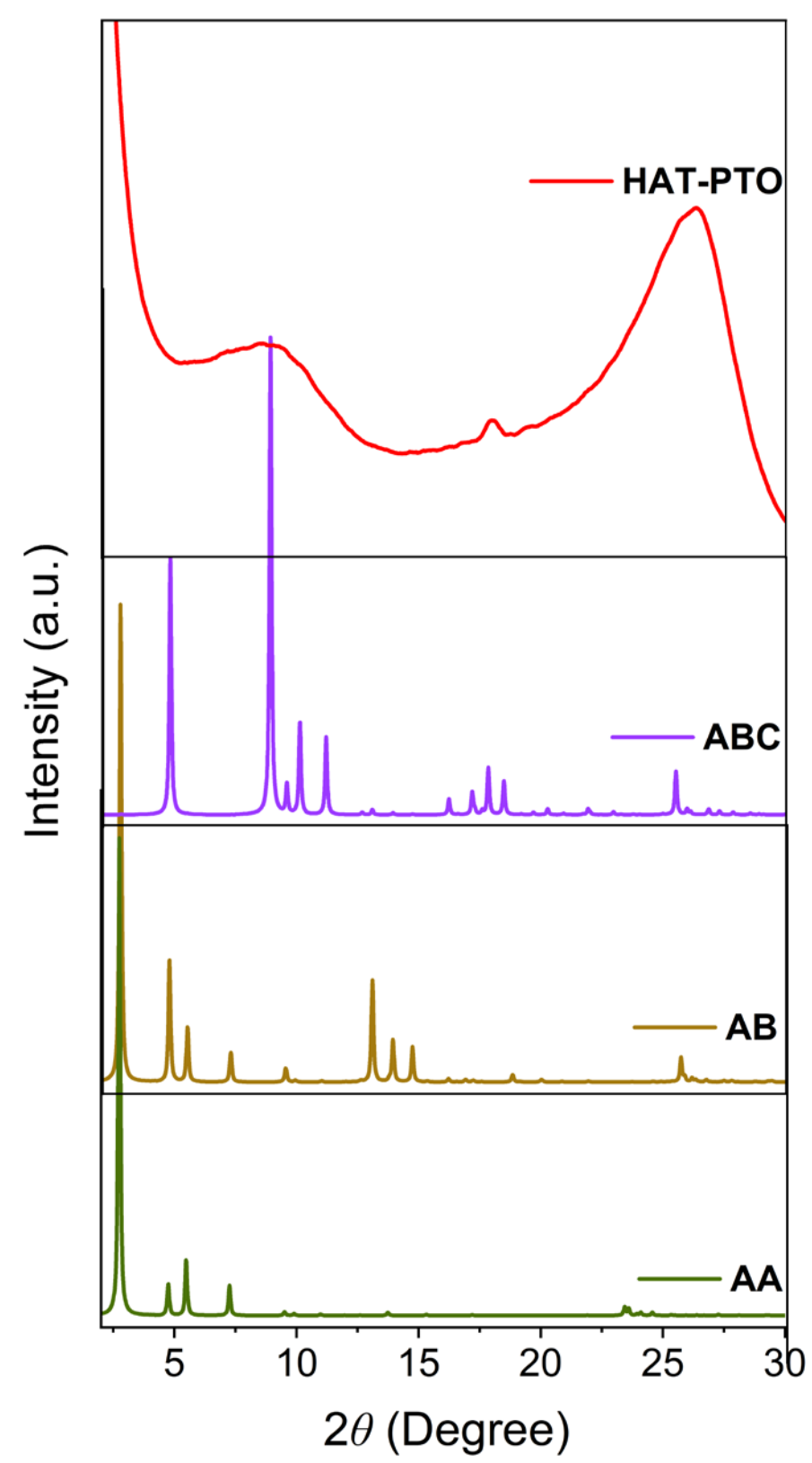

Figure S2. Simulated (AA, AB and ABC models) versus experimental PXRD for **HAT-PTO**.

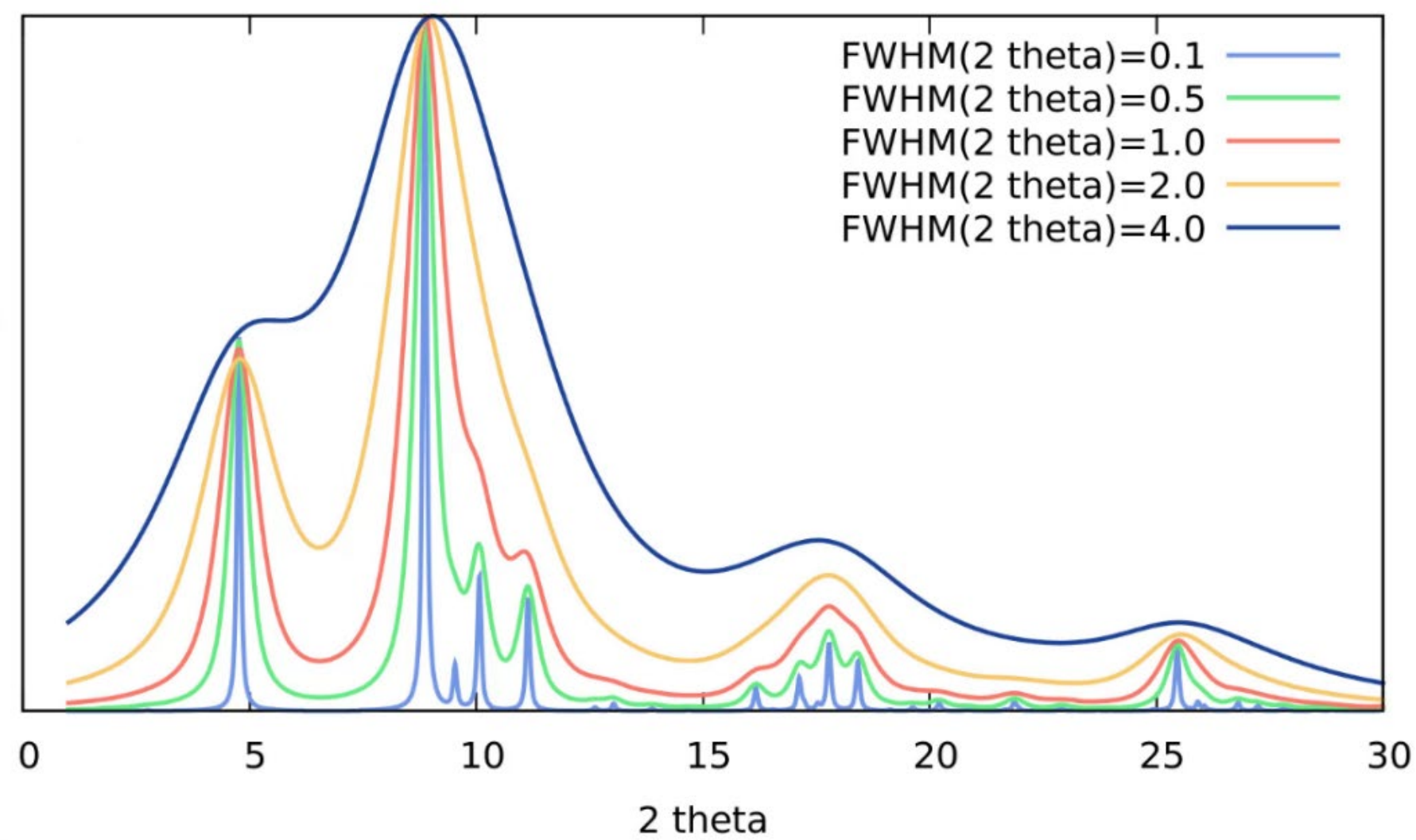

Figure S3. Simulated PXRD for the ABC bulk stacking computed with different FWHM and peak broadening parameters.

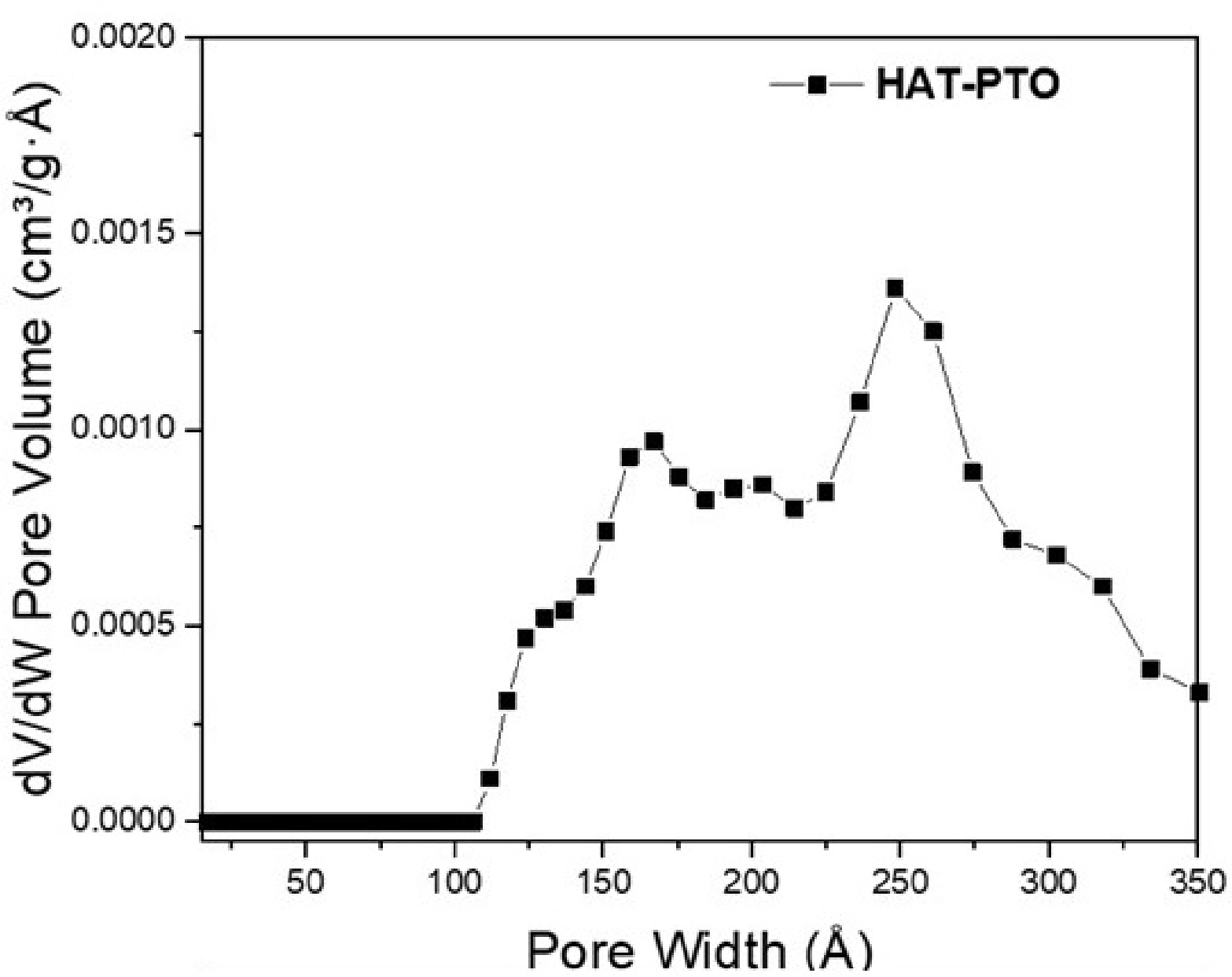

Figure S4. Pore size distribution of **HAT-PTO**.

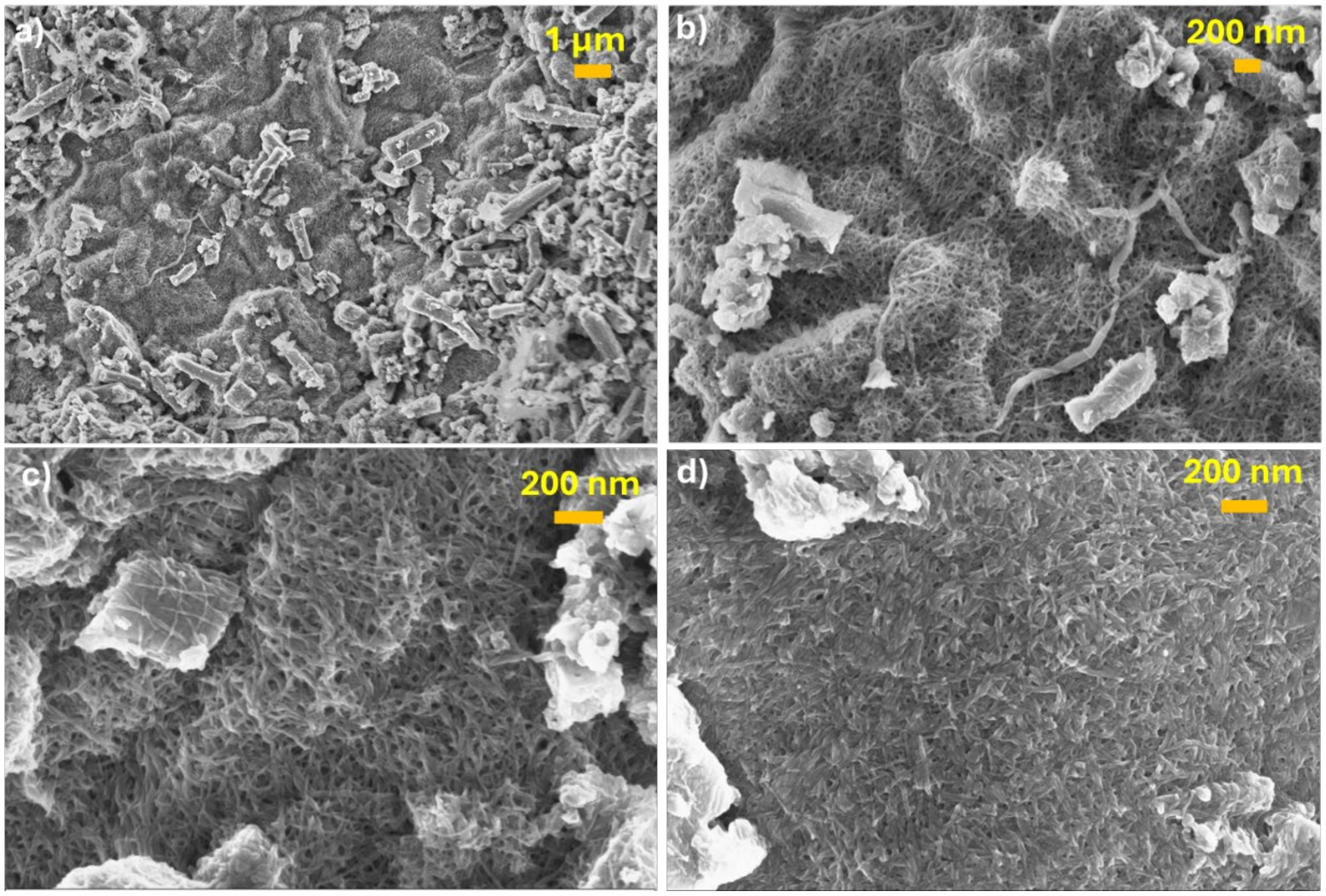

Figure S5. (a-d) SEM images of **HAT-PTO**.

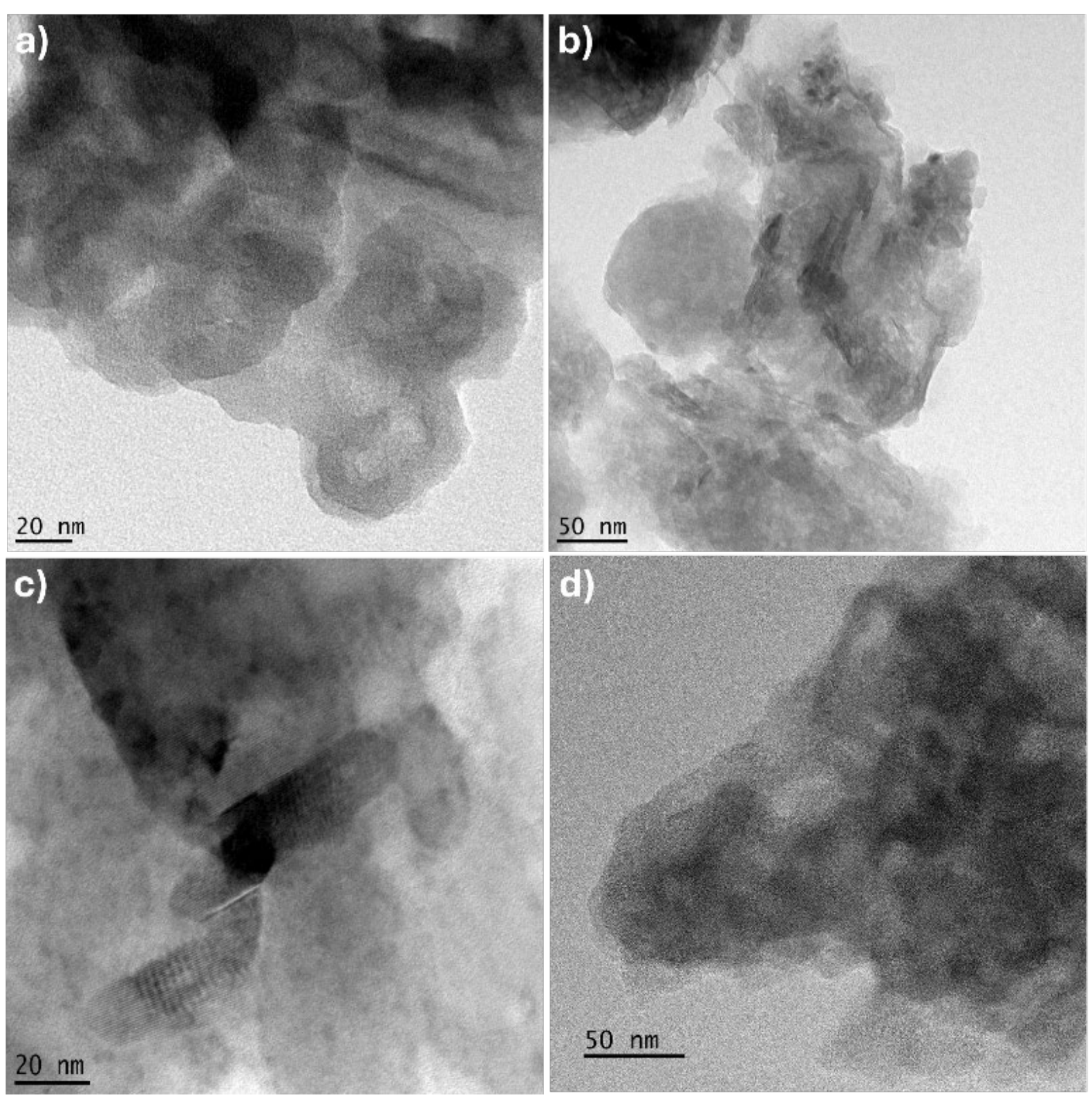

Figure S6. **(**a-d**)** TEM images of **HAT-PTO**.

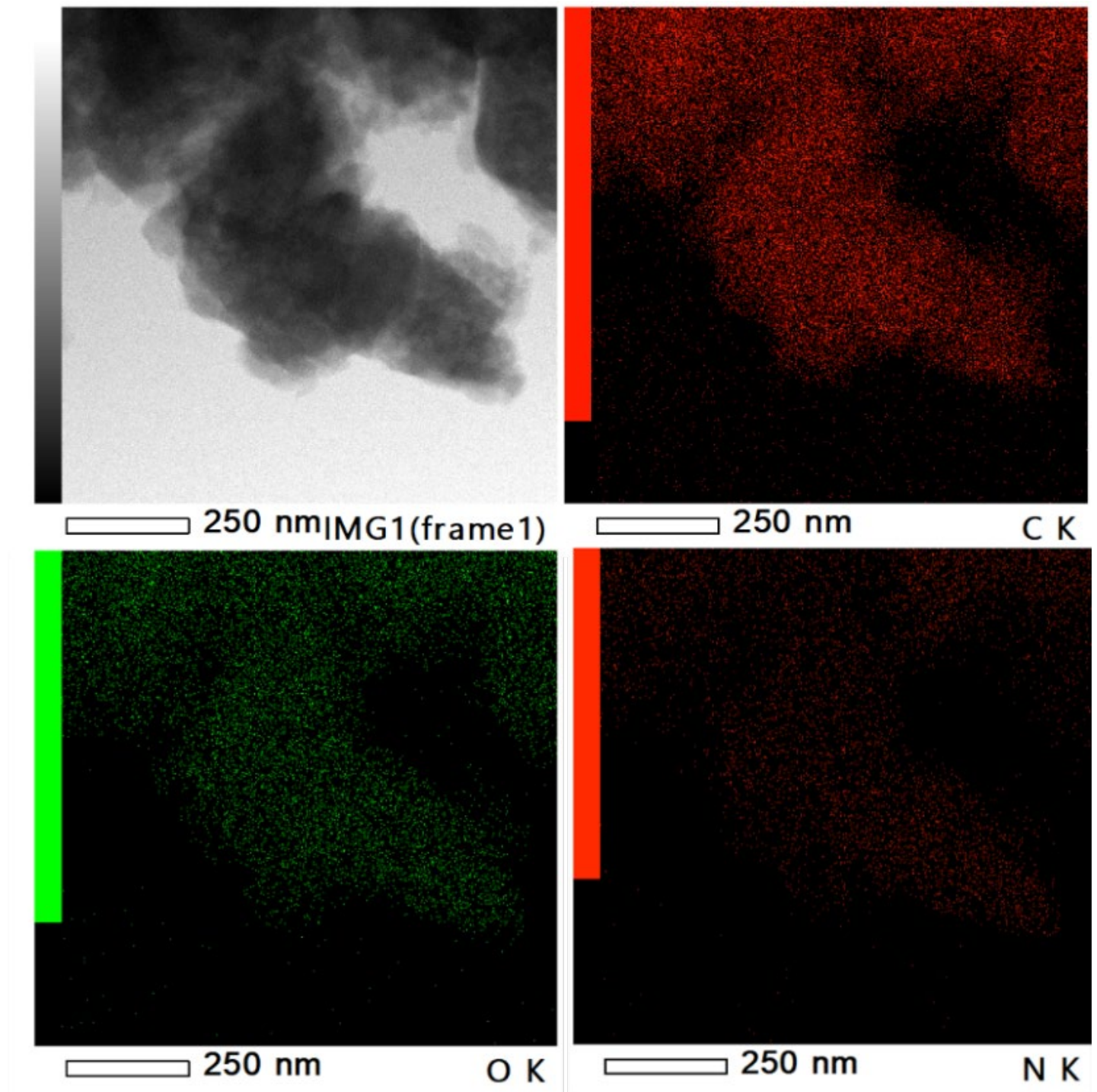

Figure S7. STEM-EDX elements mapping images of **HAT-PTO**.

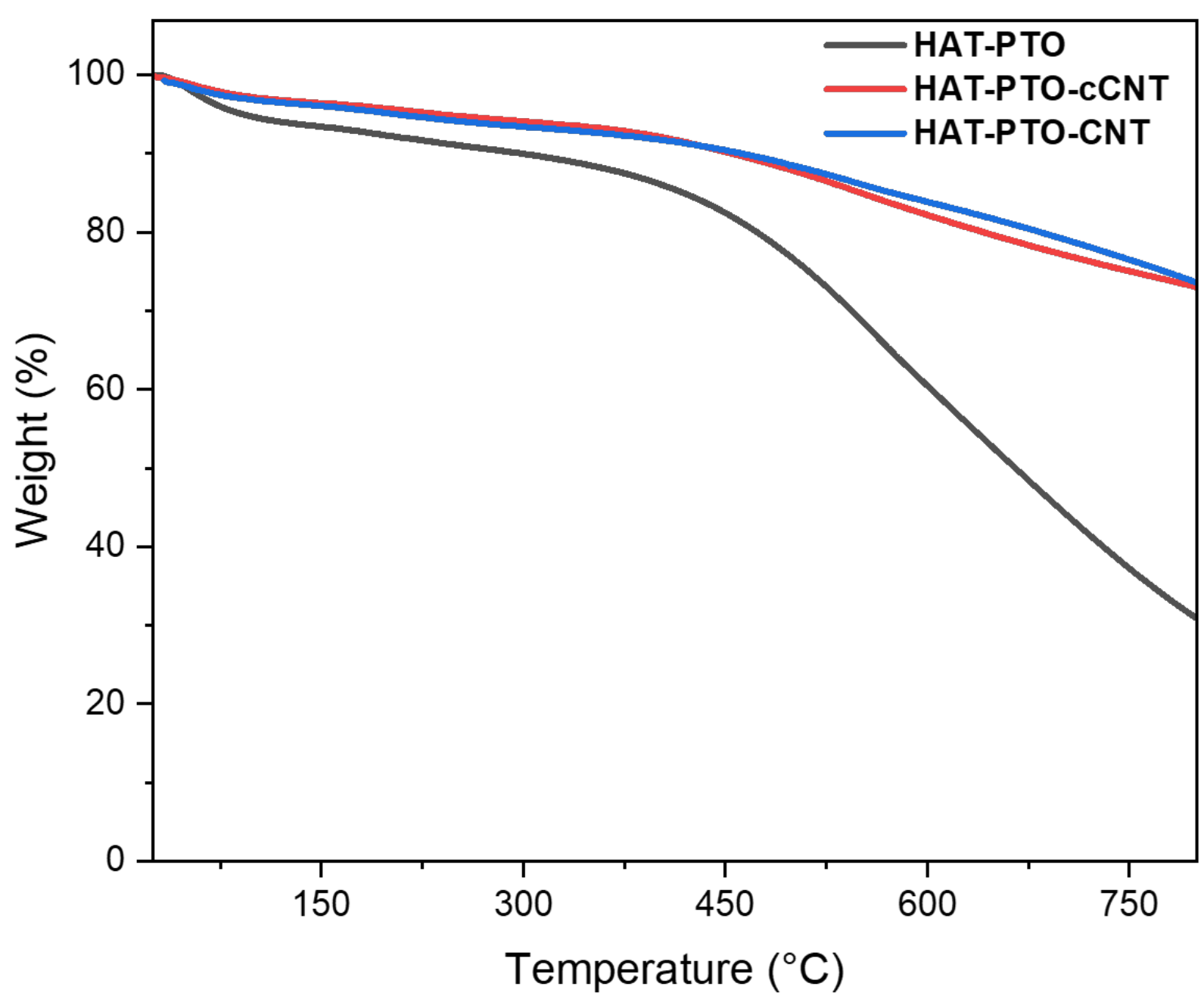

Figure S8. TGA of **HAT-PTO**, **HAT-PTO-cCNT** and **HAT-PTO-CNT**.

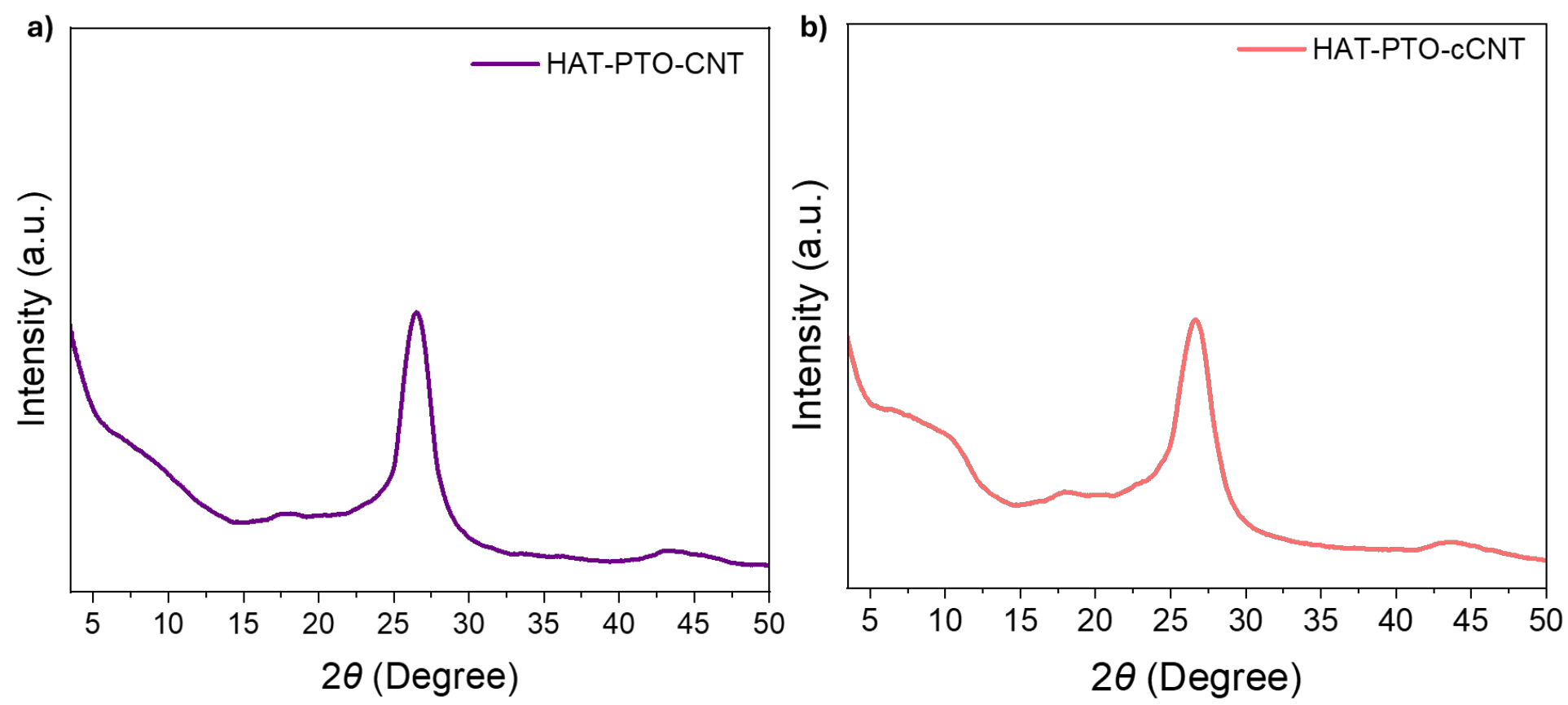

Figure S9. PXRD spectra of (a) **HAT-PTO-CNT** and (b) **HAT-PTO-cCNT**.

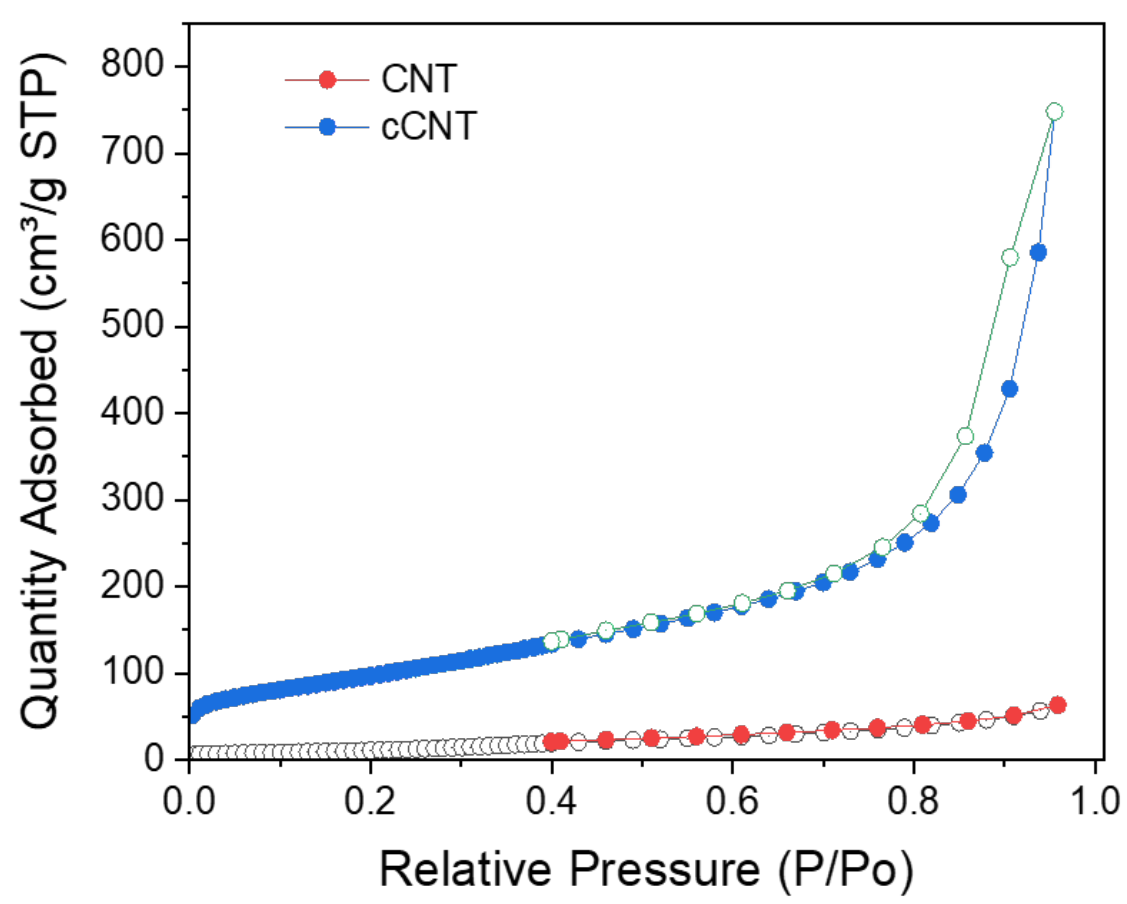

Figure S10. N_2 adsorption (filled symbols) and desorption (empty symbols) isotherms of CNT and cCNT at 77 K.

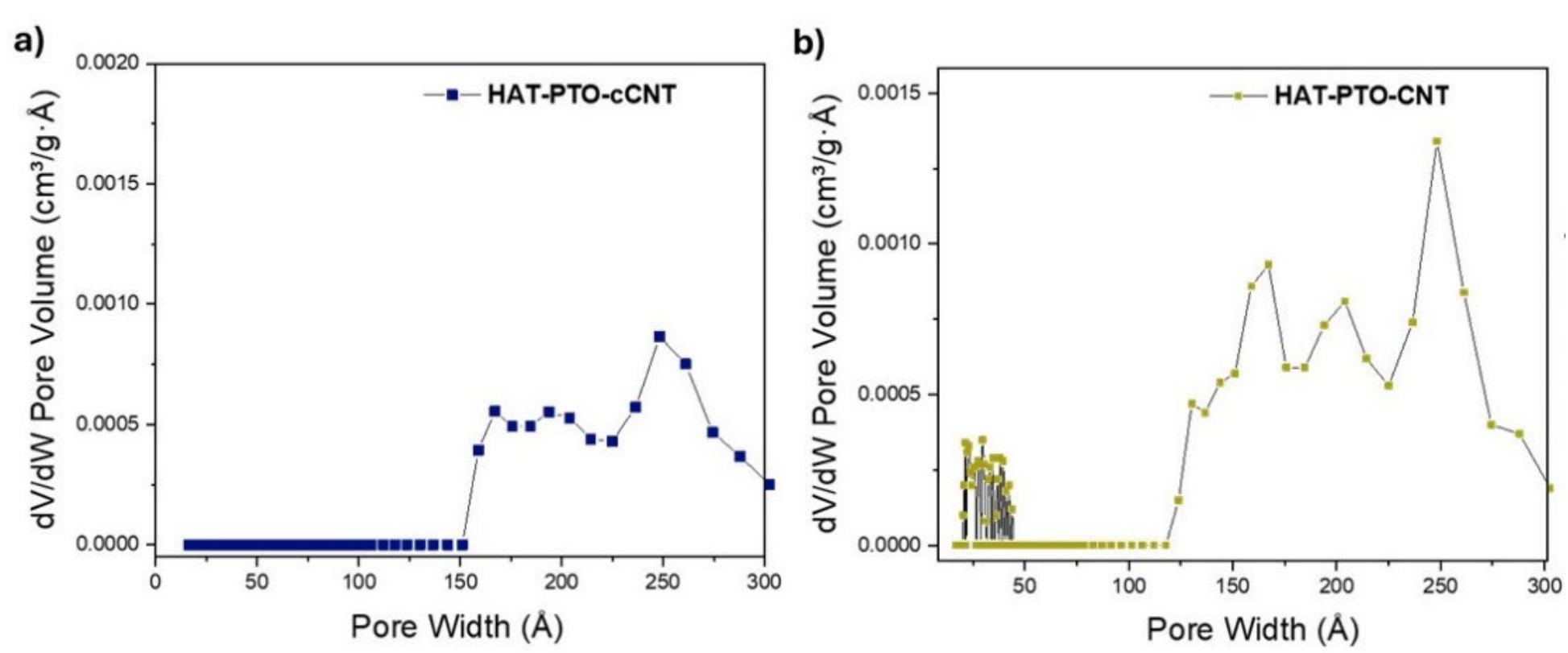

Figure S11. Pore size distribution of (a) **HAT-PTO-cCNT** and (b) **HAT-PTO-CNT**.

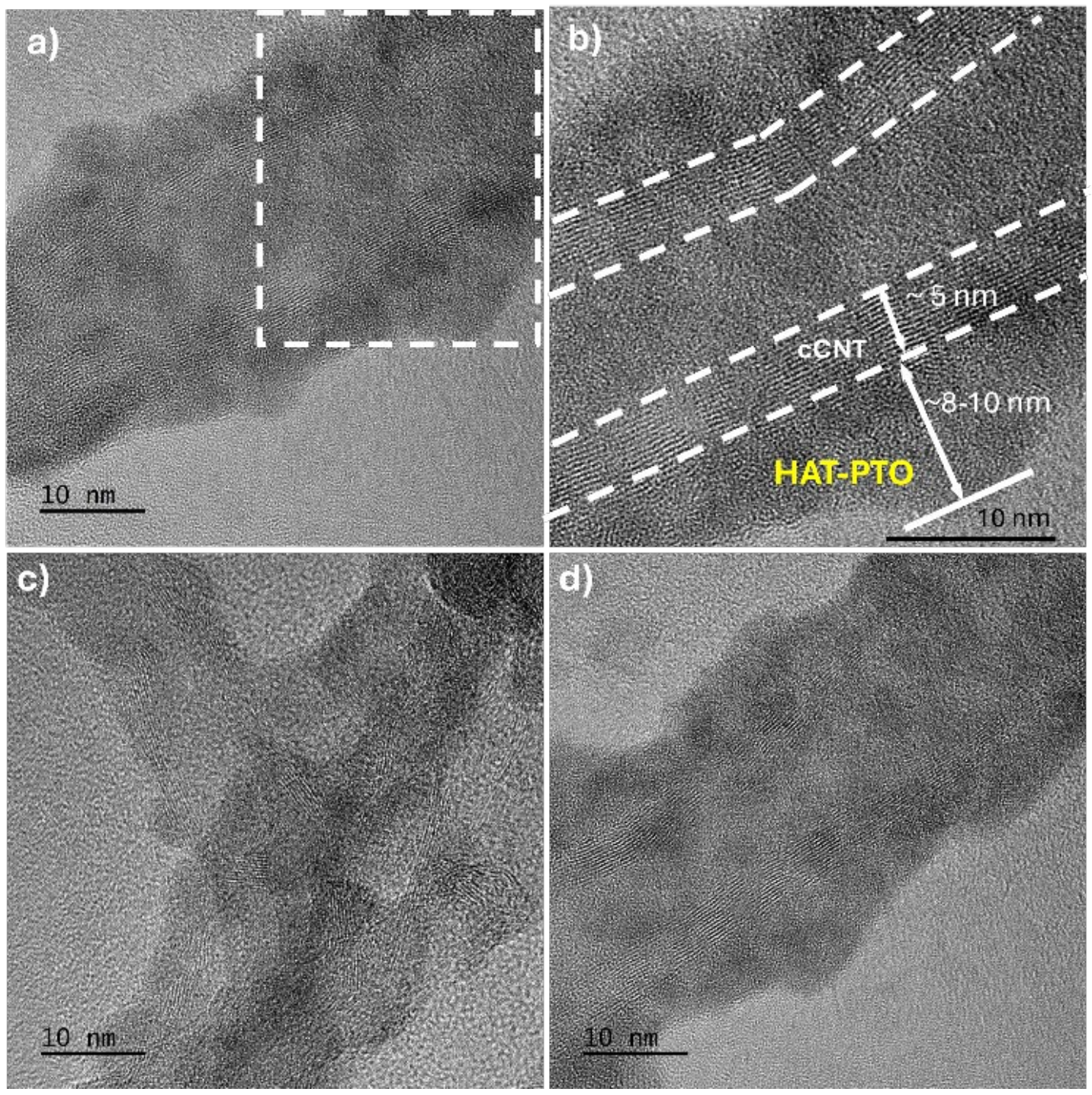

Figure S12. HRTEM images of **HAT-PTO-cCNT**.

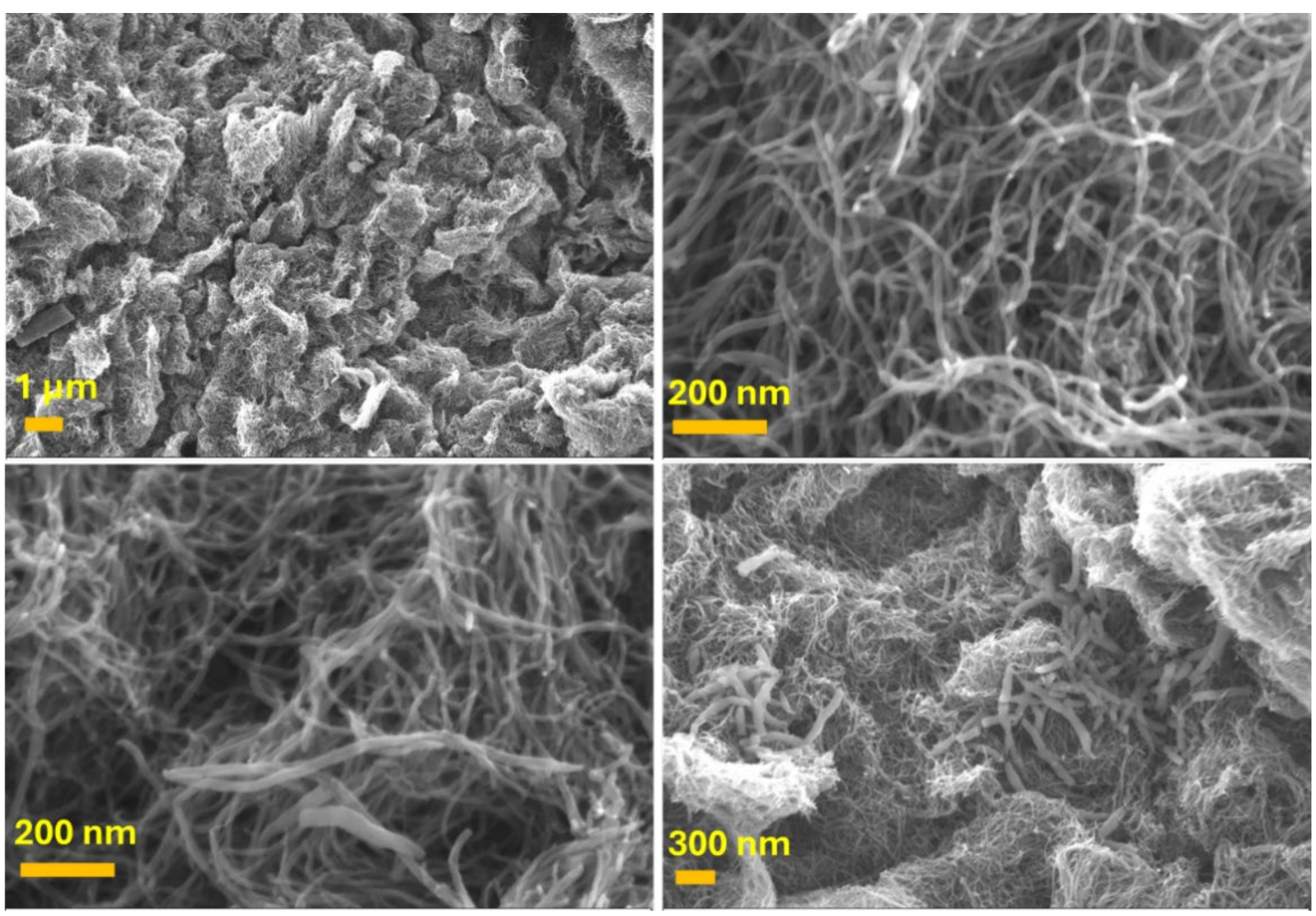

Figure S13. (a-d) SEM images of **HAT-PTO-cCNT**.

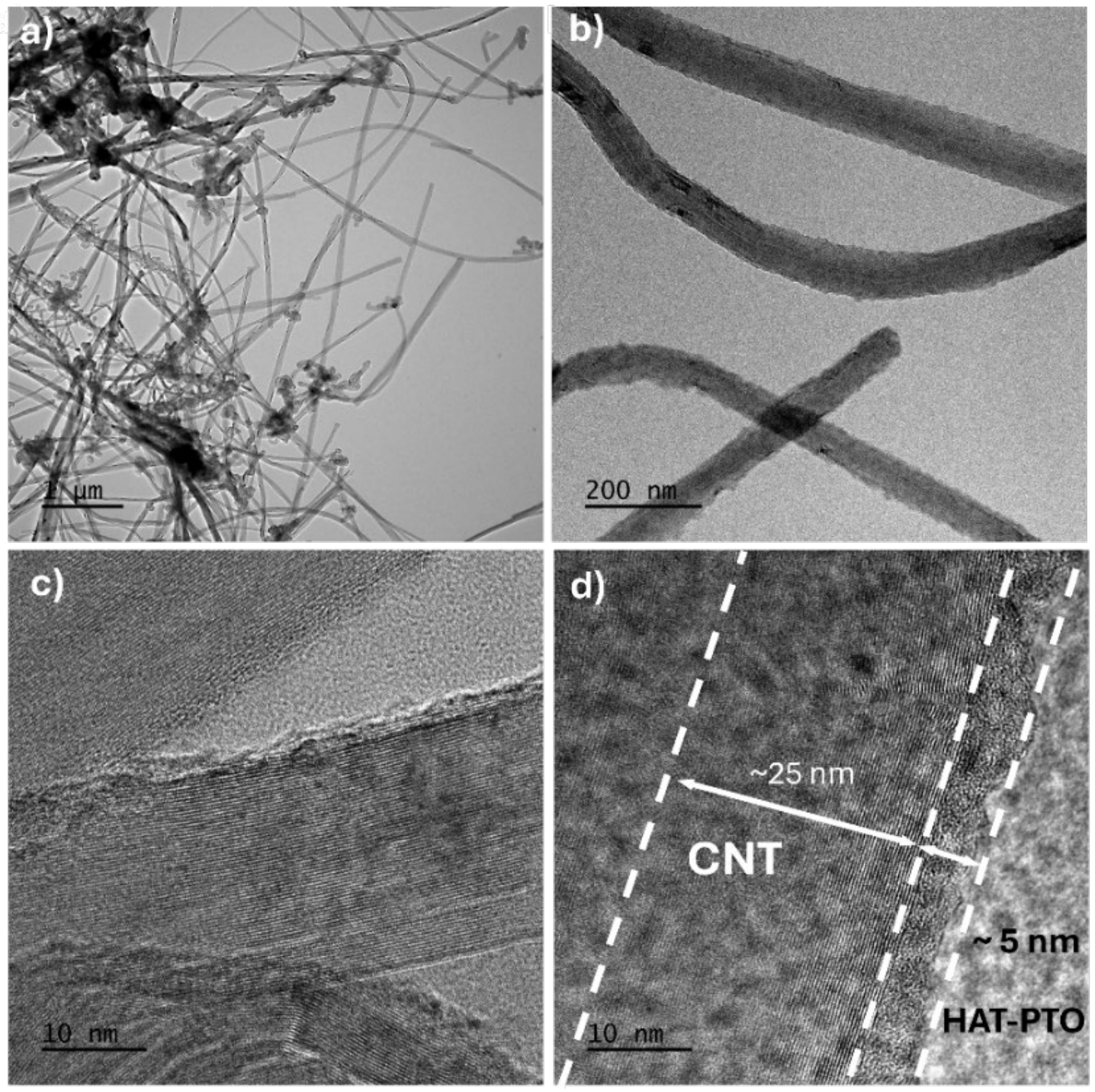

Figure S14. HRTEM images of **HAT-PTO-CNT**.

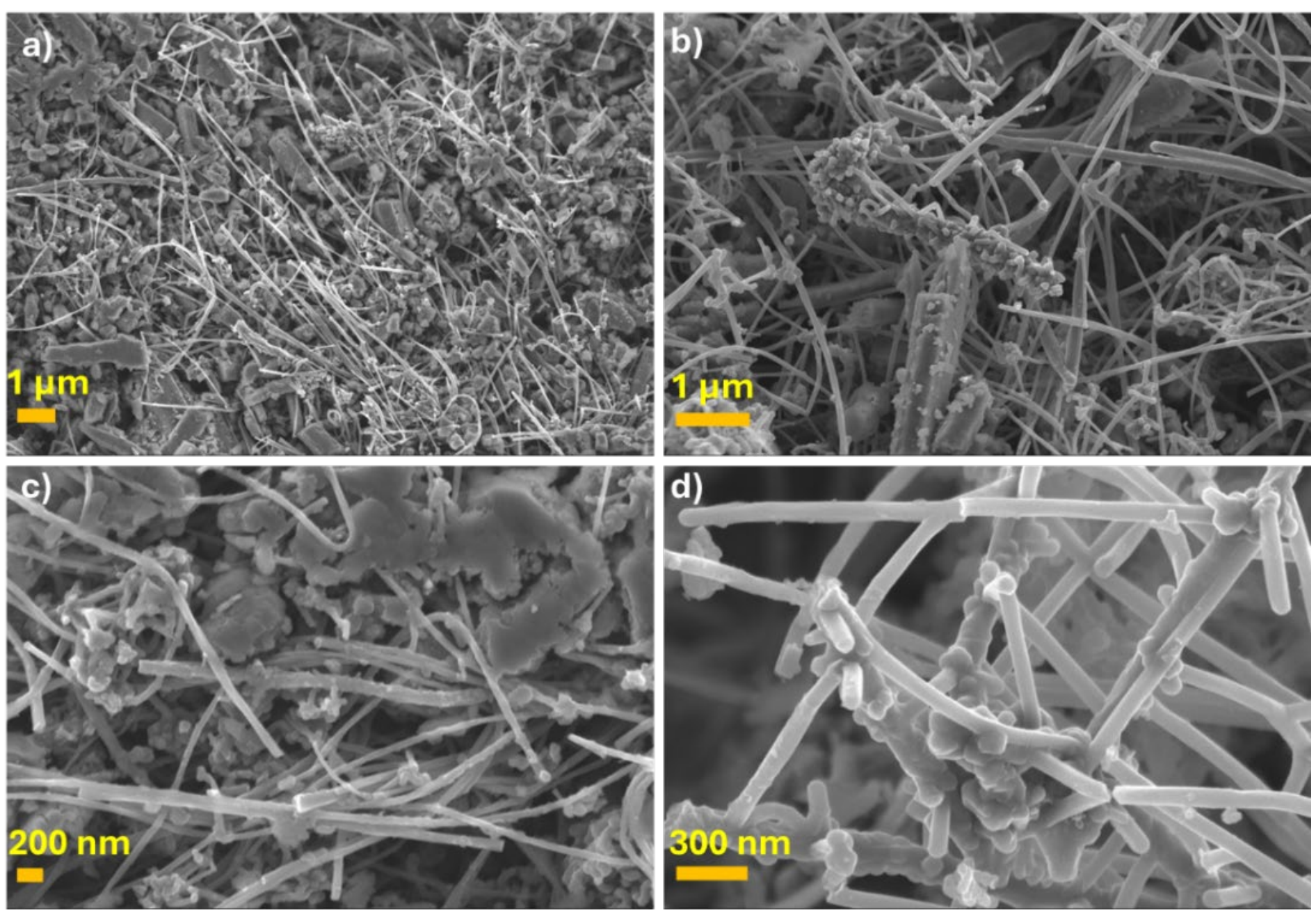

Figure S15. (a-d) SEM images of **HAT-PTO-CNT**.

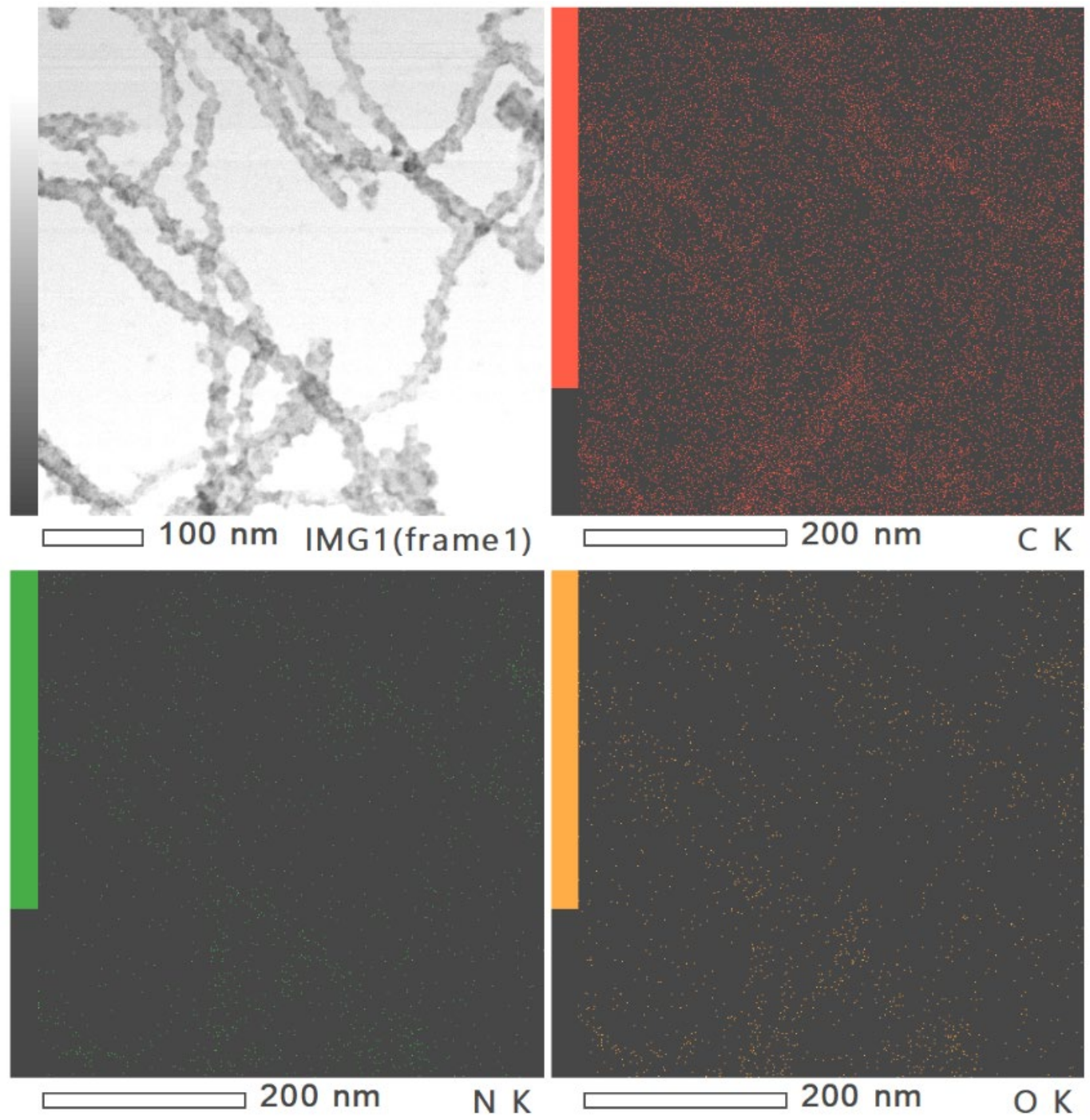

Figure S16. STEM-EDX elements mapping images of **HAT-PTO-cCNT**.

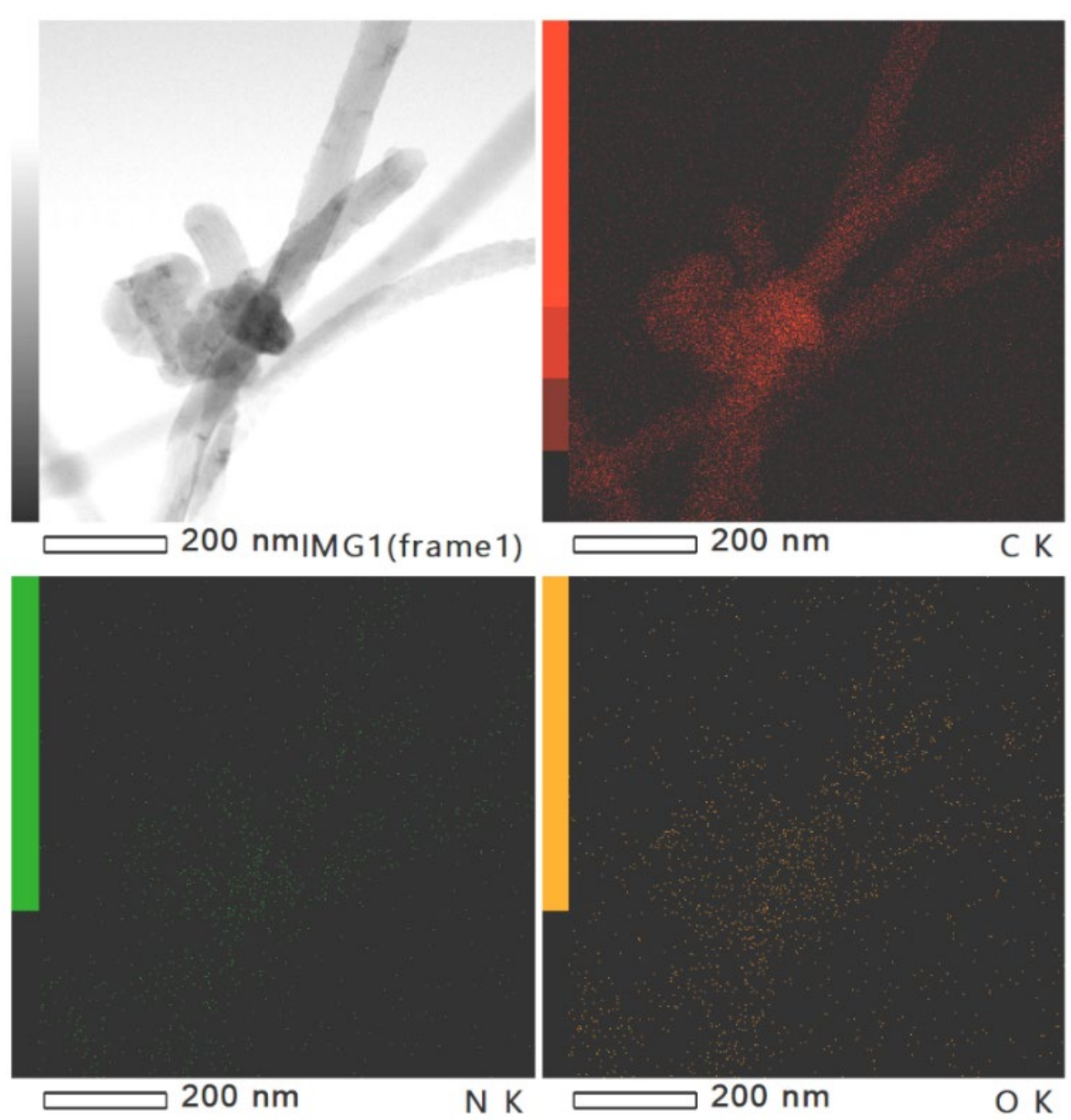

Figure S17. STEM-EDX elements mapping images of **HAT-PTO-CNT**.

3. Electrical conductivity measurements

Van der Pauw method for the determination of electrical conductivity of pressed pellets. Disk-shaped pellets were prepared by pressing in a home-made die under a pressure of ~2 GPa using a hydraulic press. The diameter of the pellets was 6 mm. The pellets were placed onto a Corning glass slide and mounted on the sample stage of a microprobe station (Instec HCP421V-MPS). All measurements were performed at room temperature. Four symmetrically placed contacts were applied to the edge of the disks using silver paste (DuPont CP4929N-100), and electrical contacts were made by gently touching the top surfaces of the silver contacts with gold-plated tungsten probes (see Figure S18 for the van der Pauw contact configuration and Table S2 for measurement definitions). The probes were connected to a Keithley 4200A-SCS Parameter Analyzer via triaxial cables. Voltage-current (*V-I*) characteristics were measured by applying a bias current and recording the resulting voltage, the resistance value was extracted from the slope of the linear fit (Figure S19). This procedure was repeated in four different configurations (Table S2) to determine the average resistance and apply the correction factor (*f*) for contact geometry, which was then obtained using the following expression:

$$\frac{Q-1}{Q+1} = \frac{f}{\ln 2}\cosh^{-1}\left[\frac{e^{\left(\frac{\ln 2}{f}\right)}}{2}\right],$$

where $Q = R_A/R_B$ is the resistance ratio (see Table S2 for definitions).

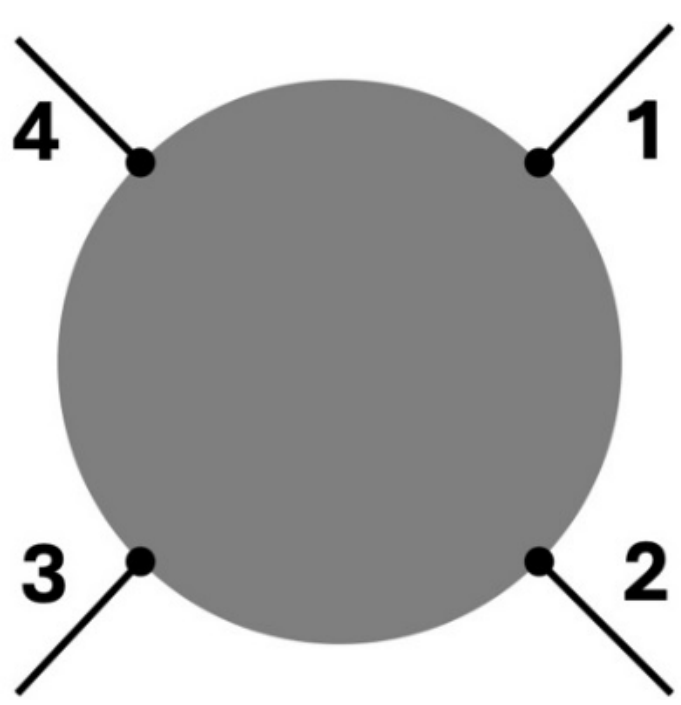

Figure S18. Schematic of the Van der Pauw contact configuration on a disk-shaped sample.

Table S2. Van der Pauw measurement definitions.

Measurement	R_1	R_3	R_2	R_4
Current sourced via probes #	1-4	2-3	1-2	4-3
Voltage measured via probes #	2-3	1-4	4-3	1-2
Resistance, $R_{A,B}$	$R_A = \frac{R_1 + R_3}{2}$		$R_B = \frac{R_2 + R_4}{2}$	

The measured V-I curves for the 3 different samples are shown in Figure S19. Using the procedure described above, we can estimate the electrical conductivity of the sample employing the formula,

$$\sigma = \frac{\ln 2}{\pi t}\frac{1}{R_{ave} * f},$$

where $R_{ave} = \frac{R_A + R_B}{2}$ is the average resistance and t is the sample thickness. Table S3 shows a summary of the results obtained.

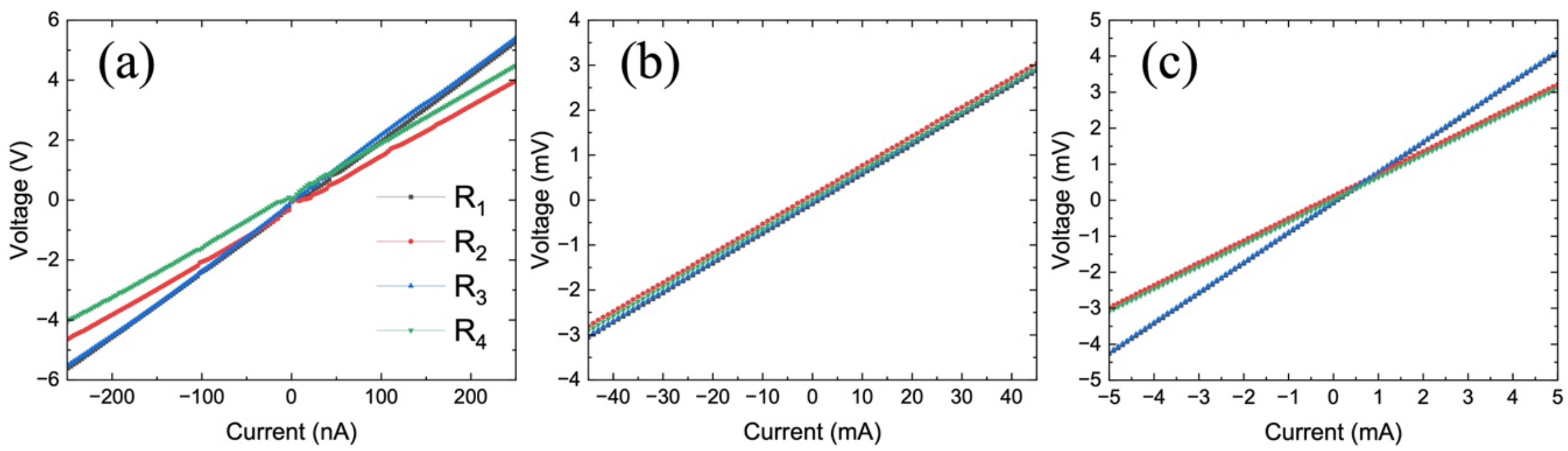

Figure S19. Voltage vs current characteristics measured for pressed pellets of a (a) **HAT-PTO**, (b) **HAT-PTO-CNT** and (c) **HAT-PTO-cCNT**.

Table S3. Average electrical conductivity values for **HAT-PTO**, **HAT-PTO-CNT**, and **HAT-PTO-cCNT** pressed pellets measured using a van der Pauw four-probe setup at room temperature.

Sample	***t* (mm)**	**R_{ave} (Ω)**	***Q***	***f***	**σ (S/cm)**
**HAT-PTO**	0.58	19.6 x 10^6	1.27	0.995	1.95 x 10^{-7}
**HAT-PTO-CNT**	1.09	0.065	1.02	1.000	3.09 x 10^1
**HAT-PTO-cCNT**	0.90	0.729	1.35	0.992	3.39

4. Calculation of theoretical specific capacity of HAT-PTO

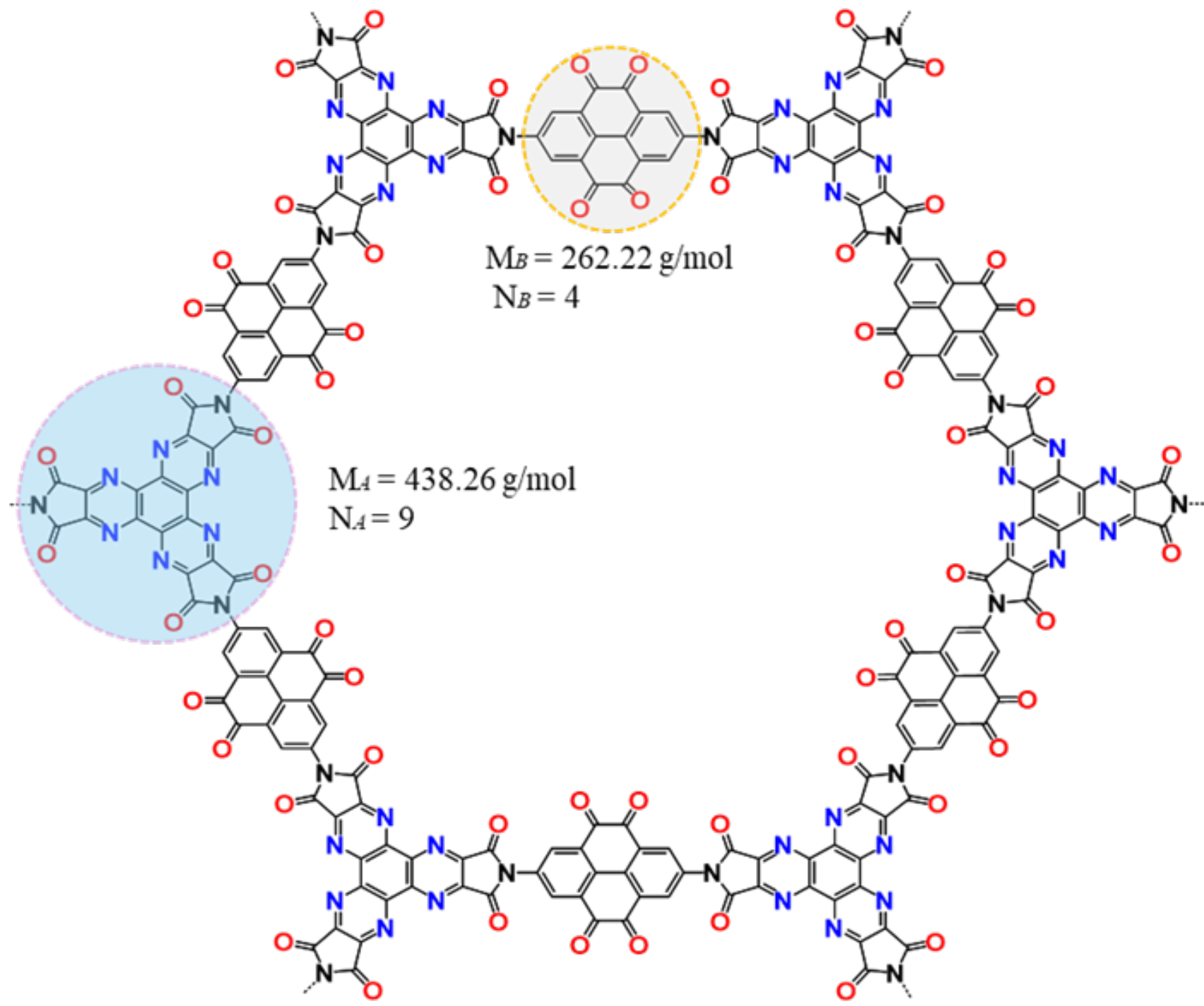

Scheme S2. Chemical structure of **HAT-PTO** based on HAT and PTO redox-active units with their corresponding molecular weight and number of electrons transferred in redox reactions.

The theoretical specific capacity (Q_{theo}) of **HAT-PTO** is calculated using the following equation: $Q_{theo} = 26801\times (n/M_w)$ (mAh g^{-1})

where n is the number of electrons transferred in redox reactions per repeating unit of **HAT-PTO** and M_w is the molecular weight of the repeating unit of **HAT-PTO** (g mol^{-1}).

The repeating unit in **HAT-PTO** consists of 1/3 of an A unit and 1/2 of a B unit. The molecular weights of A and B units in the HAT-PTO are 438.26 (M_A) and 262.22 (M_B) g mo1^{-1}, respectively. Therefore, the molecular weight of the repeating unit in **HAT-PTO** is estimated to be M_w = $[1/3\times M_A+1/2\times M_B]$ = 277 g mol^{-1}. The number of electrons associated with the redox reaction of unit A and B is 9 (N_A) and 4 (N_B) respectively. Thus, the number of electrons (n) involved in each repeating unit of **HAT-PTO** is $[1/3\times N_A+1/2\times N_B]$ = 5.

By applying above mentioned equation,

the theoretical specific capacity of **HAT-PTO** is = 26801 × (5/277) mAh g^{-1} = 484 mAh g^{-1}

5. Electrochemical measurements

All electrochemical performances were measured using CR2032-type coin cells assembled in an argon-filled glove box (MBraun; H_2O and O_2 levels <1 ppm). Lithium metal was used as counter electrode and HAT-PTO, HAT-PTO-CNT or HAT-PTO-cCNT as working electrodes, respectively.

1 M LiTFSI dissolved in a 1:1 (v/v) mixture of DOL and DME was selected as the electrolyte. The working electrode was prepared by mixing HAT-PTO with conductive carbon black Super C65 and polyvinylidene fluoride (PVDF) binder in a mass ratio of 60:30:10. *N*-Methyl-2-pyrrolidone was added during the mixing process to form a homogeneous slurry. The slurry was then coated onto an aluminum foil substrate and dried at 60 °C in a vacuum for 12h.

For the hybrid materials incorporating carbon nanotubes and carboxyl functionalized-carbon nanotubes a different composition was used: HAT-PTO-CNT or HAT-PTO-cCNT, conductive carbon black Super C65, and PVDF binder mixed in a mass ratio of 80:10:10. The as-obtained films were punched into disks with a diameter of 12 mm. A Li disk was used as the anode with Celgard 2325 membrane as the separator. 1 M LiTFSI in DOL/DME (v:v = 1:1) was used as the electrolyte. The mass loading of active material in the electrodes was in the range of 0.5-0.8 mg cm^{-2}. Galvanostatic charge/discharge test was conducted in the voltage range of 1.2-4 V (vs. Li+/Li) on a LAND CT2001A instrument (Wuhan, China), Cyclic voltammetry (CV) at different scan rates (0.5, 1.0, 1.5 and 2.0 mV/s with a window voltage of 1.4 – 4.0 V vs. Li+/Li) and Electrochemical Impedance Spectroscopy (EIS) were performed on VIONIC potentiostat and Intello software from Metrohm. Electrochemical impedance spectroscopy (EIS) was performed to better investigate the interphase evolution during cycling. A three-electrode Swagelok-type cell was assembled, using lithium metal as both counter and reference electrodes, and HAT-PTO or HAT-PTO-cCNTh as the working electrode, respectively. The measurements were carried out in 1 M LiTFSI dissolved in a 1:1 (v/v) mixture of DOL and DME. Electrochemical impedance spectroscopy was performed using a single-sine sinusoidal excitation of ±5 mV amplitude, applied over a frequency range from 0.1 Hz to 100 kHz with logarithmic spacing and 10 frequency points per decade. Before each measurement, the cell was held at a fixed potential for 1 hour to ensure equilibrium conditions were reached.

Impedance spectra were recorded at open circuit voltage (OCV) and after the 10th, 20th, 30th, 40th, 100th, and 300th cycles to monitor variations in resistance and assess the long-term stability of the electrode–electrolyte interphase. The resulting Nyquist plots were modelled using equivalent circuit method and fitted through a nonlinear least squares (NLLS) approach via the RelaxIS 3 software (RHD Instruments, Darmstadt, Germany). The equivalent circuit employed, denoted in Boukamp notation as Re(RiCi)W, was modified by replacing the ideal capacitor (C) with a constant phase element (Q) to more accurately capture the non-ideal electrochemical behaviour attributed to surface roughness and morphological heterogeneity at the interface.[S2,S3] All the electrochemical tests were conducted at 25 °C.

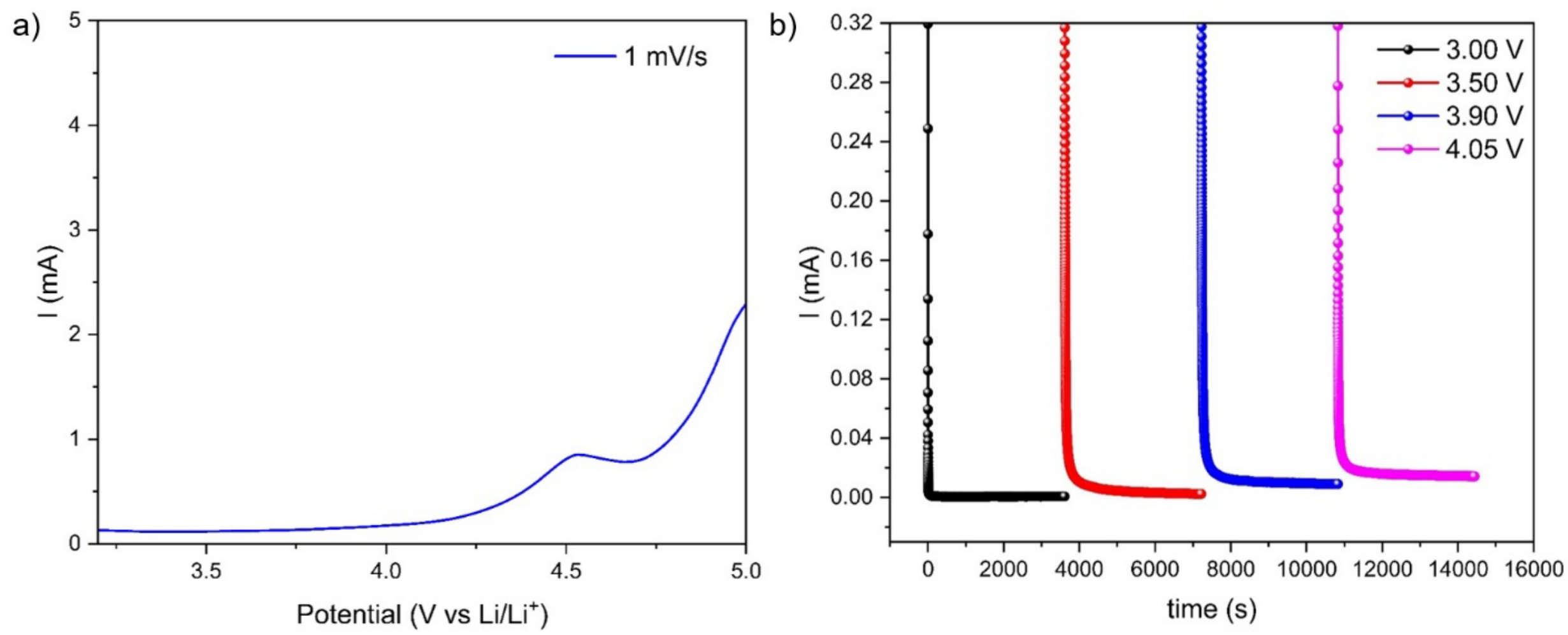

Figure S20. a) Linear sweep voltammetry (LSV) of **HAT-PTO-cCNT**. b) Chronoamperometry of the **HAT-PTO-cCNT** electrode at 3.0 V (black), 3.5 V (red), 3.9 V (blue), and 4.05 V (purple), each for 3600 s, showing stabilization of the current response even at potentials above the cycling window.

To assess any potential dissolution issues of active material, electrodes were soaked in 1 mL of each electrolyte for 48 h. As the photos below show, the active material is stable in the electrolyte, with no apparent dissolution occurring after two days of soaking.

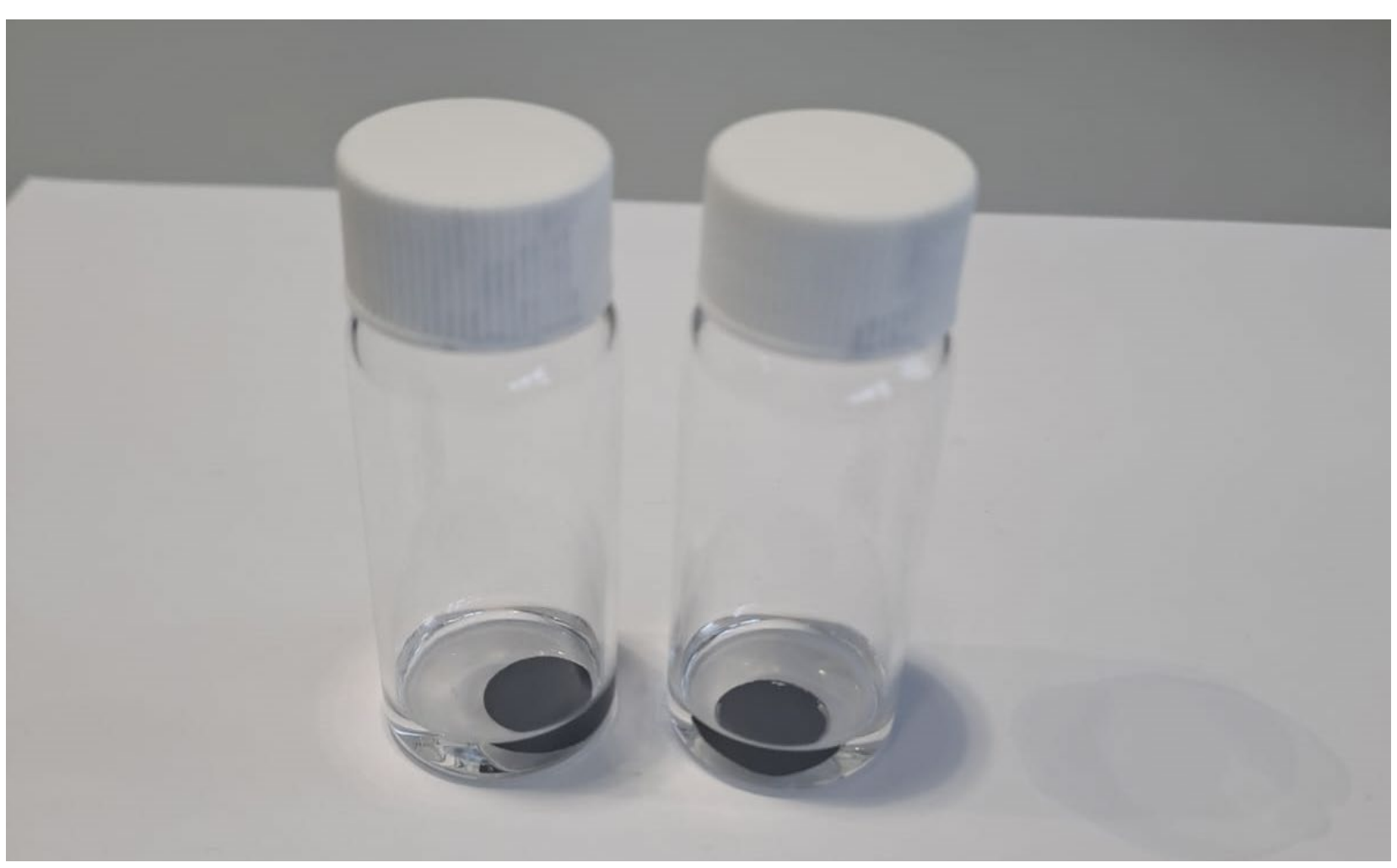

Figure S21. HAT-PTO (left) and **HAT-PTO-cCNT** (right) electrodes soaked for 48 h in the electrolyte (1 M LiTFSI in DOL/DME). No apparent dissolution of the active material was observed.

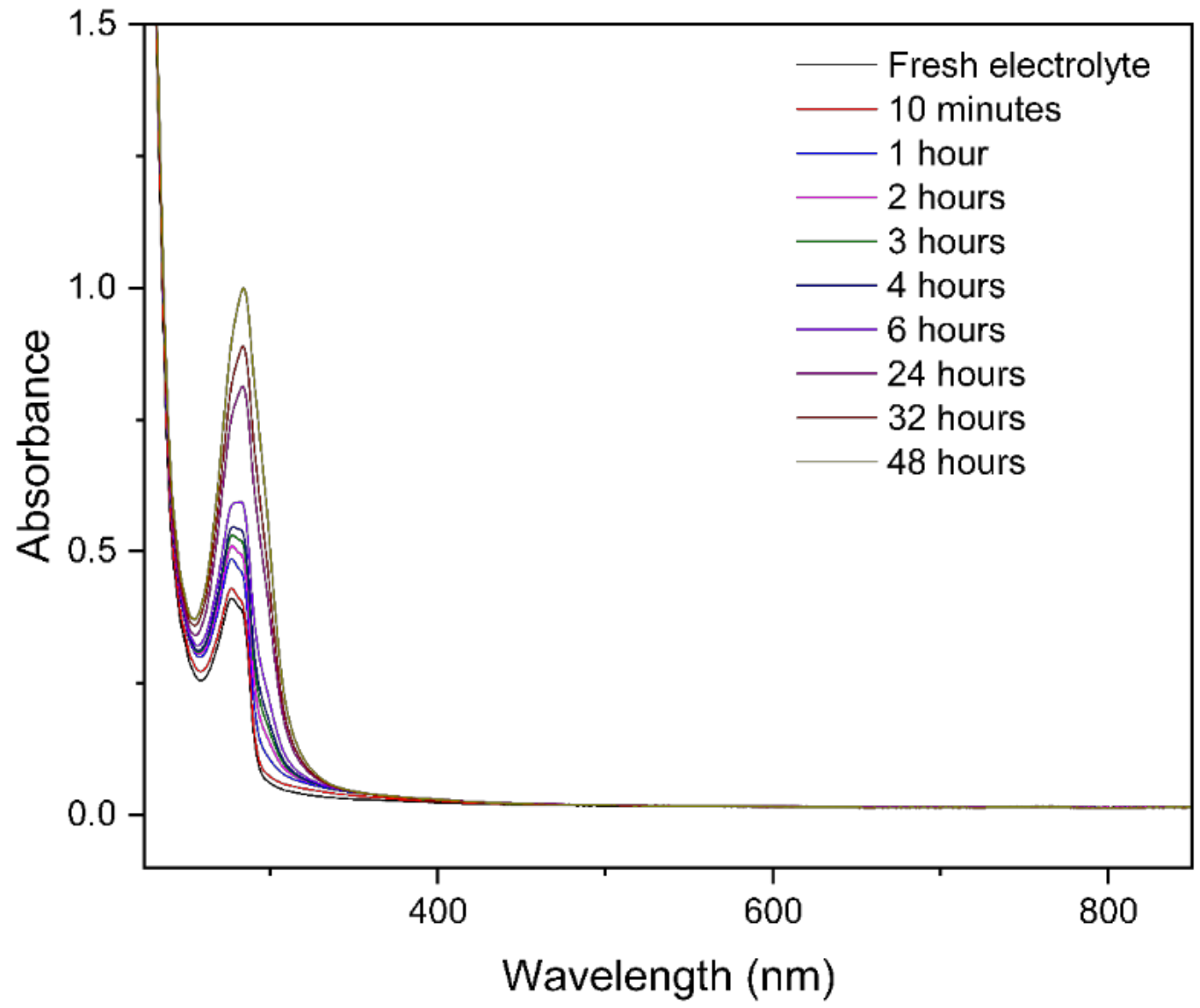

Figure S22. UV-vis spectra of fresh LiTFSI in DOL/DME electrolyte and after immersing the **HAT-PTO-cCNT** electrode for different periods of time (up to 48 h).

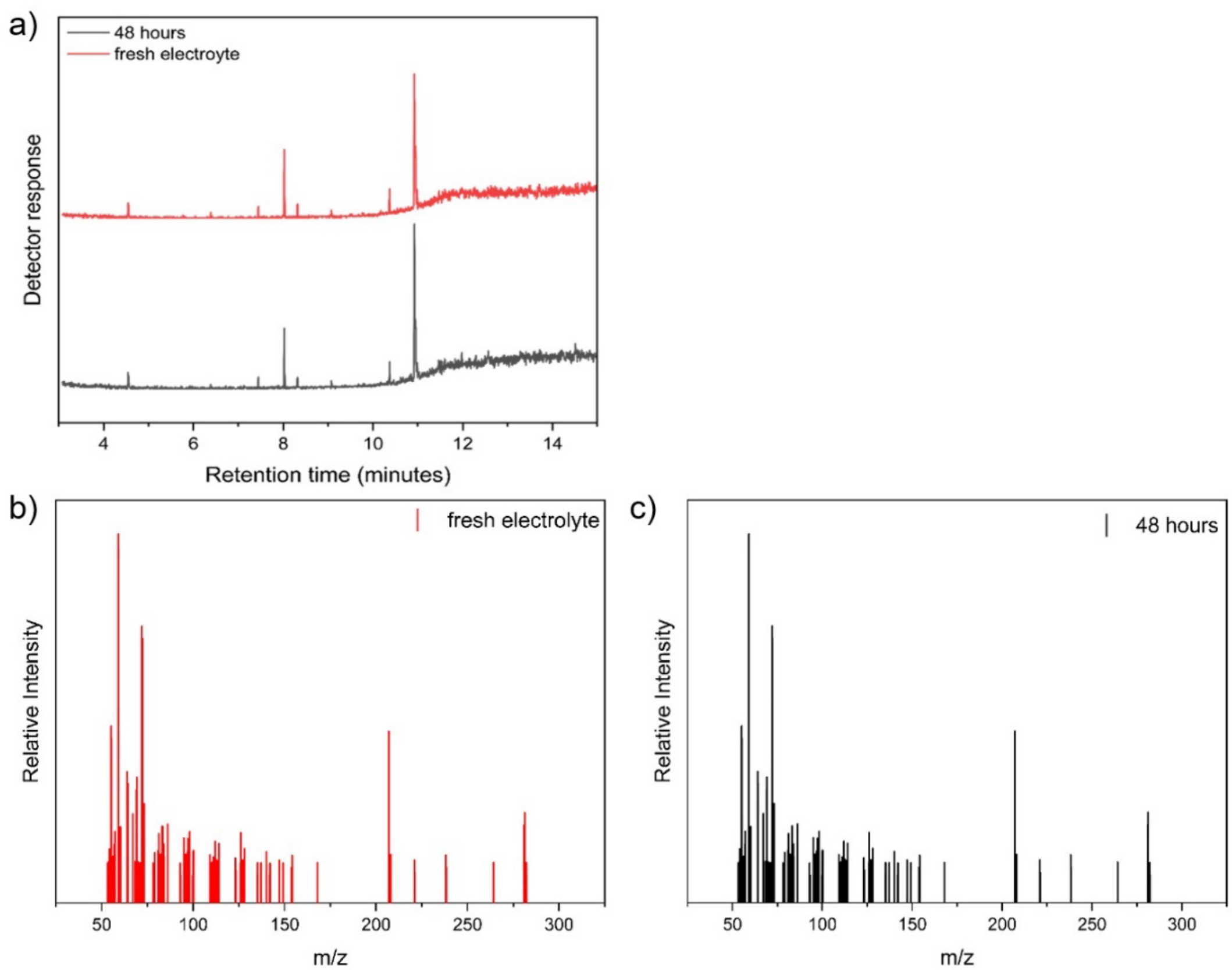

Figure S23. a) Gas chromatogram of fresh electrolyte and after immersing the **HAT-PTO-cCNT** electrode for 48 hours. Mass spectra of b) fresh electrolyte and b) after immersing the **HAT-PTO-cCNT** electrode for 48 hours.

Calculation of utilization efficiency for HAT-PTO

Carbon black electrodes were prepared by mixing C65 and PVdF (weight ratio 75:25) in NMP. The mixture was ball milled for 30 minutes at 300 rpm (Retsch PM100), then deposited on carbon-coated Al foil using a Dr. Blade. The slurry was dried at 120 °C under vacuum for 24 hours. Subsequently, 12 mm electrodes were cut out and dried again at 120 °C overnight before cell assembly. The carbon black electrode was cycled against metallic Li, with one Celgard 2325 separator soaked in 1 M LiTFSI in DOL/DME (1/1, vol%). Cycling was performed at the same current density as HAT-PTO cycling to enable capacitance subtraction. The subtraction was carried out using the following formula:[S4]

$$C_{AM} = C_{measured} - \frac{m_{CB}}{m_{AM}} C_{CB}$$

where m_{CB} and m_{AM} are masses of carbon black and active material in the cathode, respectively (in this case 3/6=0.5).

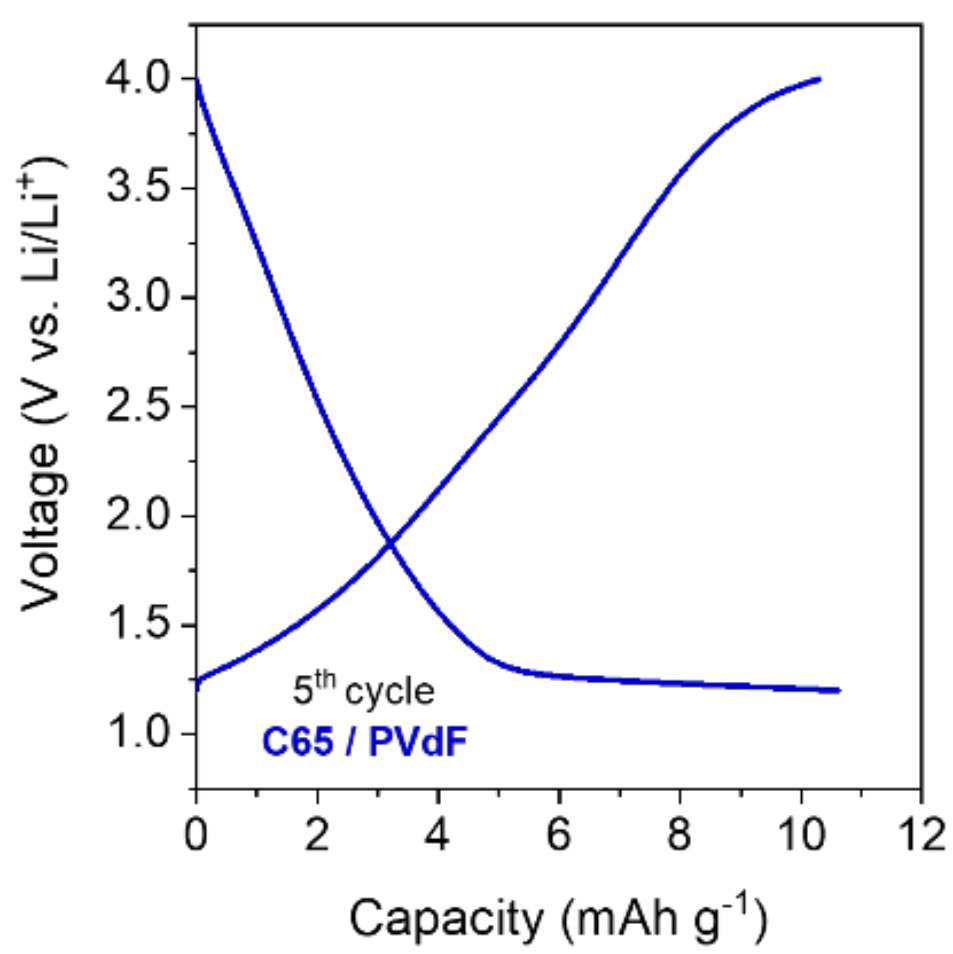

Figure S24. Voltage profile of C65/PVdF electrode obtained in the 5th cycle.

The measured capacity at C/10 of **HAT-PTO** was 162 mAh g^{-1}. The measured carbon black capacitance under the same conditions was 10.6 mAh g^{-1}, resulting in an active material capacity of 155 mAh g^{-1}. The theoretical capacity of **HAT-PTO** is 484 mAh g^{-1}, which results in a capacity utilization of 32%.

Calculation of utilization efficiency for HAT-PTO-cCNT

The utilization efficiency of HAT-PTO in the **HAT-PTO-cCNT** composite was calculated as follows:

$$\text{Utilization Efficiency} = \frac{Q_{\text{HAT PTO real}}}{Q_{\text{HAT PTO theoretical}}} X\ 100 \qquad \text{(Eq. S1)}$$

Where Q_{real} is the experimental discharge capacity after subtracting the capacity contribution from cCNT:

$$Q_{HAT\ PTO,real} = Q_{exp} - \left(\frac{W_{cCNT}}{W_{HAT\ PTO}}\ Q_{cCNT}\right) - \left(\frac{W_{cCB}}{W_{HAT\ PTO}} Q_{cCB}\right) \qquad \text{(Eq. S2)}$$

Where Q_{exp} is the experimental capacity of HAT-PTOcCNT obtained from the rate capability at C/10 of current.

W_{cCNT}, $W_{HAT\ PTO}$ and W_{CB} are the weight % of c-CNT, HAT-PTO, and of the carbon black in the electrode composition respectively.

Q_{cCNT} and Q_{CB} are the capacities of bare cCNT and bare carbon black, respectively.

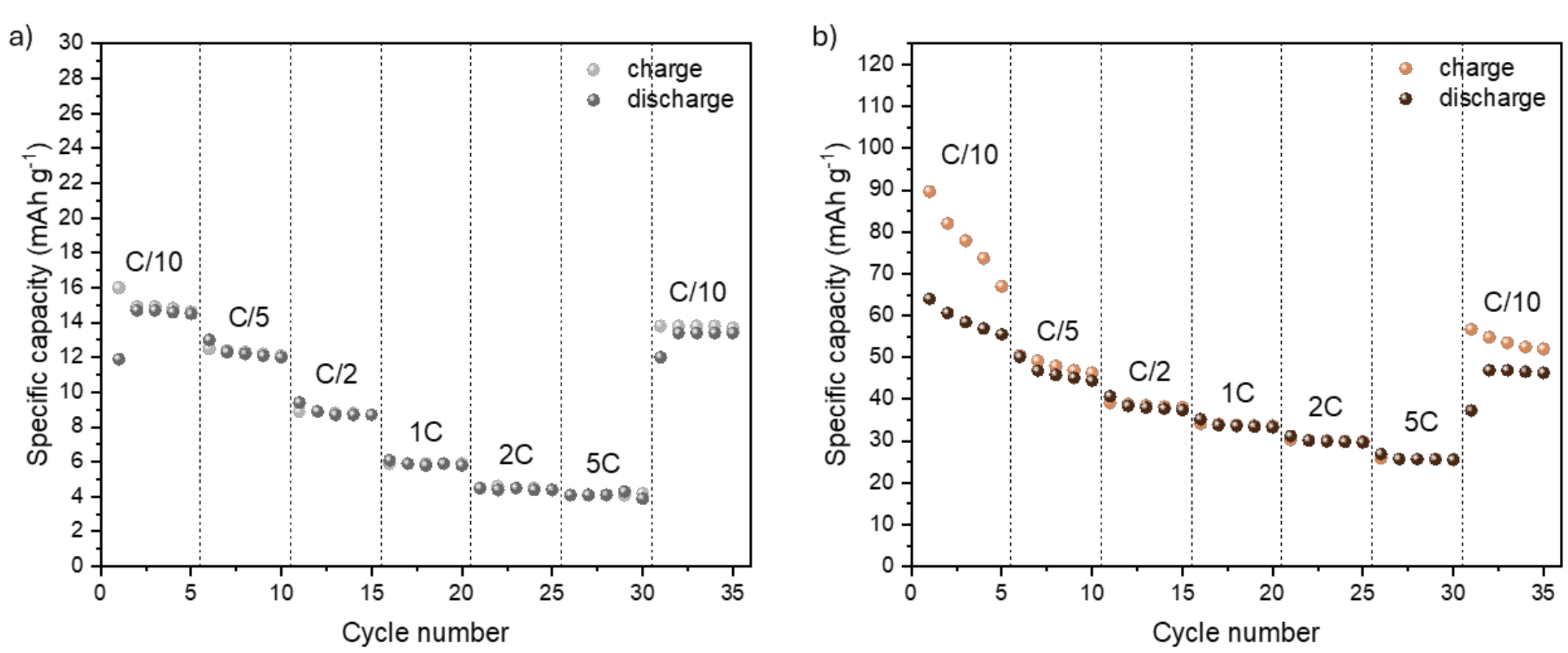

Figure S25. a) Rate capability of pure carbon black, and b) of bare cCNT.

The measured capacity at C/10 of **HAT-PTO-cCNT** was 397 mAh g^{-1}. The measured carbon black capacitance under the same conditions was 15 mAh g^{-1}, and for cCNT was 57, resulting in an active material capacity of 352 mAh g^{-1}. The theoretical capacity of **HAT-PTO** is 484 mAh g^{-1}, which results in a capacity utilization of 73%.

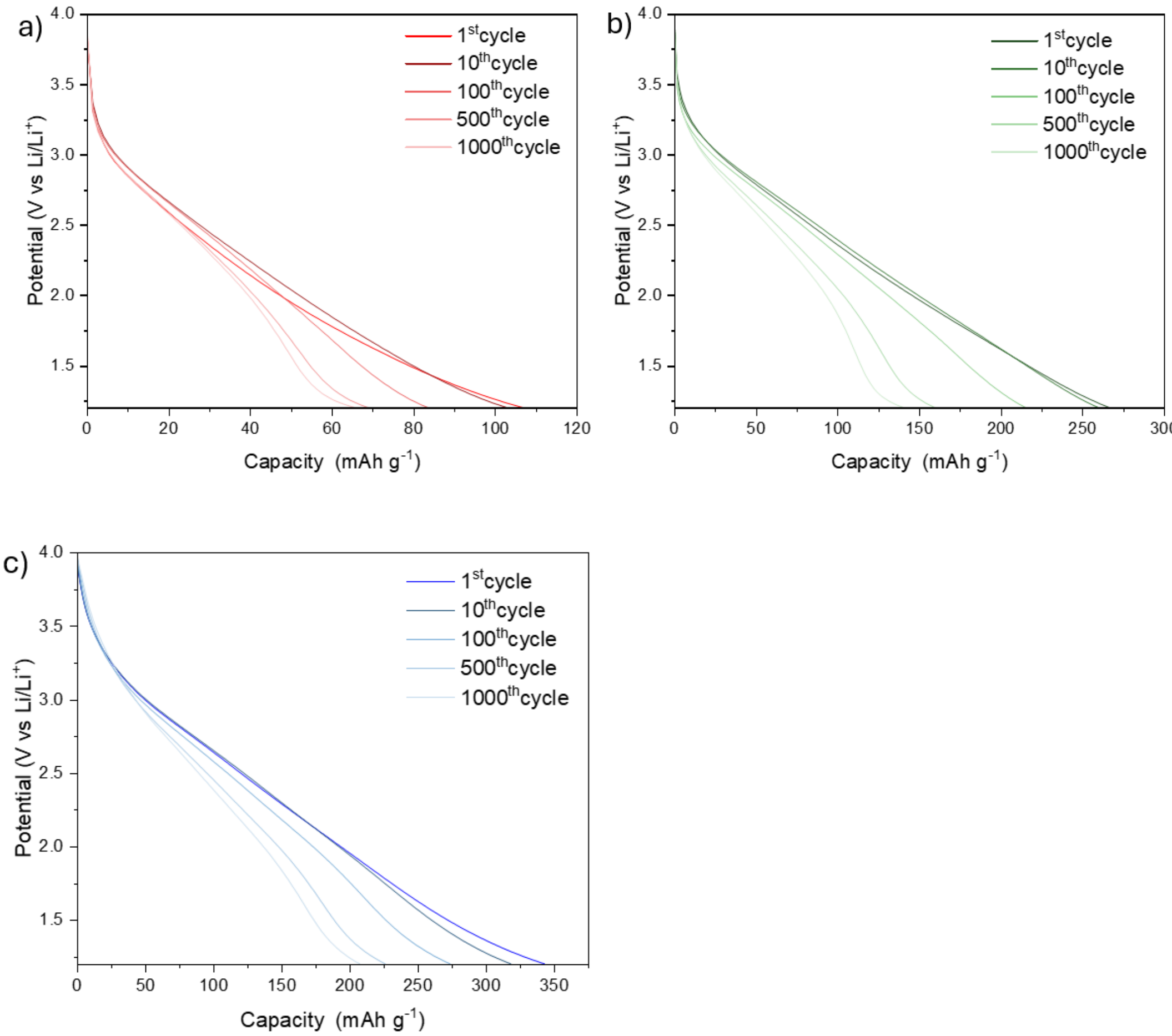

Figure S26. Galvanostatic charge–discharge profiles at 2C for: a) **HAT-PTO**, b) **HAT-PTO-CNT**, and c) **HAT-PTO-cCNT** at selected cycle numbers.

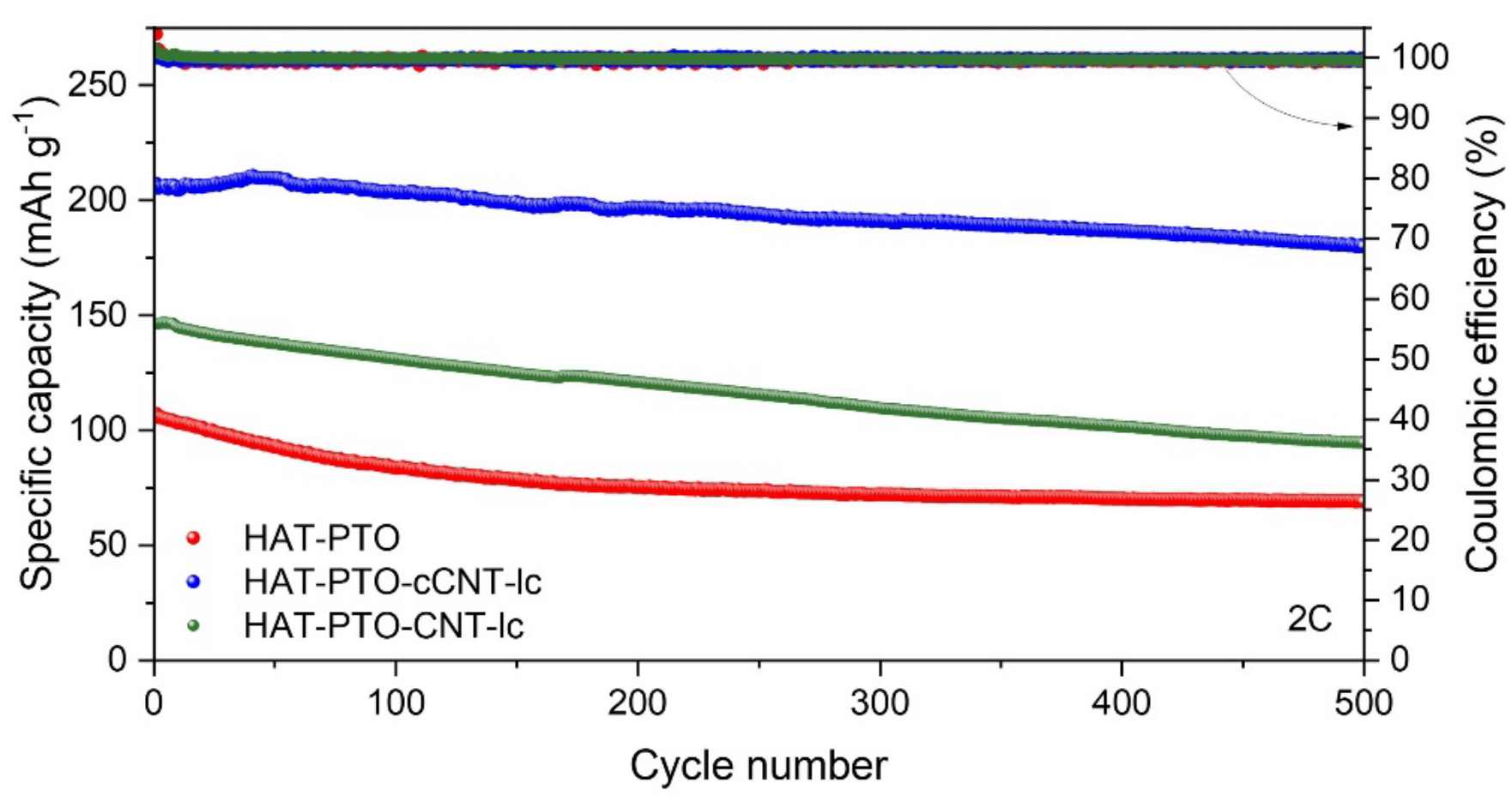

Figure S27. Cycling performances of **HAT-PTO**, **HAT-PTO-CNT-lc** and **HAT-PTO-cCNT-lc** with the same amount (60%) of active material in the electrodes at 2C.

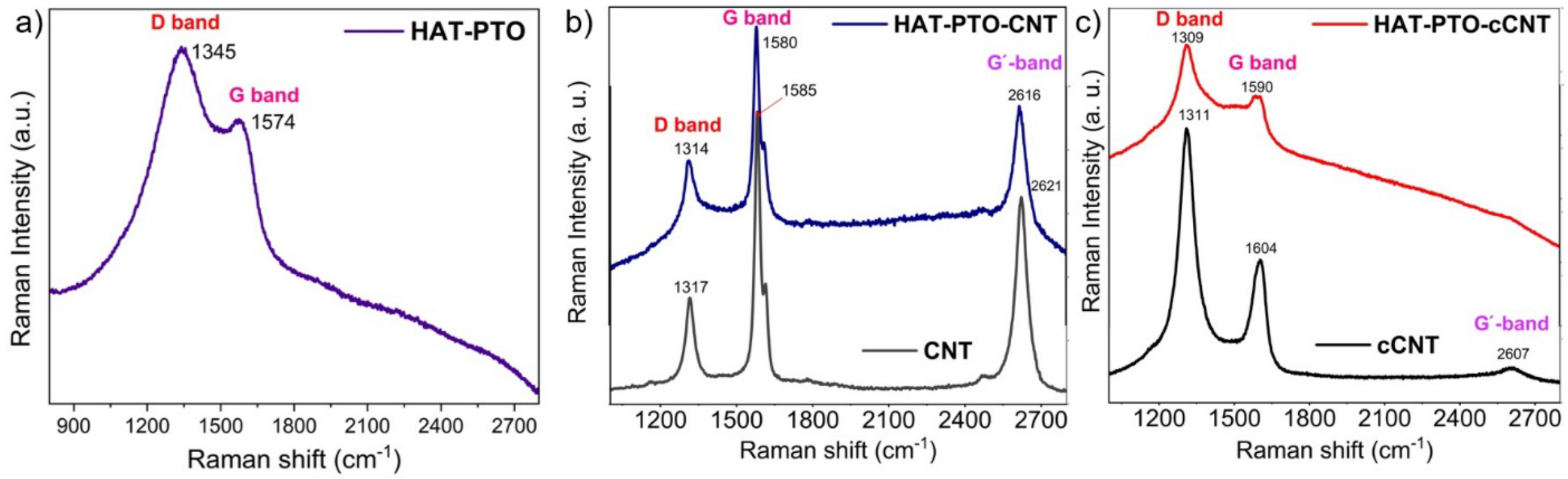

Figure S28. Raman spectra of a) **HAT-PTO**, b) **HAT-PTO-CNT** and CNT, c) **HAT-PTO-cCNT** and cCNT (λ = 785 nm). The Raman spectrum of HAT-PTO-cCNT shows a shift in the G band from 1604 cm^{-1} in pristine cCNT to 1590 cm^{-1} in the hybrid, confirming the electronic coupling between the polymer and cCNTs. In the case of HAT-PTO-CNT, a smaller Raman shift (from 1585 cm^{-1} in CNT to 1580 cm^{-1} in the hybrid) was observed.

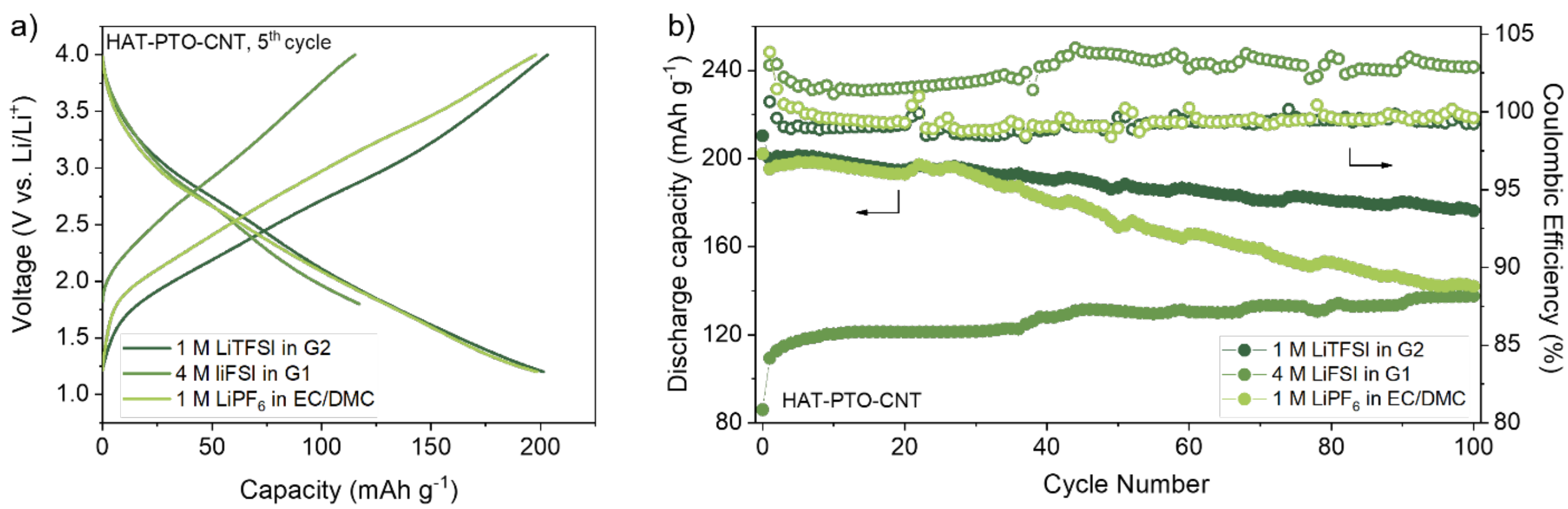

Figure S29. a) Galvanostatic profiles and b) discharge capacity of **HAT-PTO-cCNT** using different electrolytes at 2C (G1: LiTFSI in DME; G2: LiTFSI in DEGDME).

Table S4. A comparative analysis on electrochemical performances: current work vs representative POP/COF-based organic cathode materials for LIBs.

Active electrode material	Theoretical capacity (mAh g^{-1})	Voltage range (V vs. Li/Li^{+})	Low-rate capacity (mAh g^{-1})	High-rate capacity (mAh g^{-1})	Long cycling performance	Ref.
**HAT-PTO-cCNT**	**484**	**1.2–4.0**	**397 mAh g^{-1} at C/10**	**225 mAh g^{-1} at 20 C (10 A g^{-1})**	**171 mAh g^{-1} after 6000 cycles at 2 C**	**This work**
**TFPPy-ICTO-COF**	361	0.05–3.0	338 at 0.1 A g^{-1}	214 at 1 A g^{-1}	99.8 % after 1000 cycles at 1.0 A g^{-1}	S5
**TFPPer-ICTO-COF**	340	0.05–3	303 at 0.1 A g^{-1}	182.3 and 53.6% at 1 A g^{-1}	96.4 % after 1000 cycles at 1.0 A g^{-1}	S5
**HATN-AQ-COF**	358	1.2–3.9	319 at 0.5 C (179 mA g^{-1})	226 at 10 C (3.58 A g^{-1})	76.9 % after 1000 cycles at 3.6 A g^{-1}	S6
**PT-COF50**	286	1.5–3.5	280 at 0.2 A g^{-1}	229 at 5 A g^{-1}	82.0 % after 3000 cycles at 2.0 A g^{-1}	S7
**COF-TRO**	275	0.5–4.5	268 at 0.1 C	124 at 1 C	99.9 % after 100 cycles at 0.1 C	S8
**PIBN-G**	280	1.5–3.5	271 at 0.1 C	193 at 10 C	86.0 % after 300 cycles at 1 C	S9
**DAAQ-TFP-COF**	151	1.5–4.0	110 at 0.02 A g^{-1}	25 at 3.0 A g^{-1}	100.0 % after 1000 cycles at 0.5 A g^{-1}	S10
**BQ1-COF**	773	1.2–3.5	300 at 0.5 C (386.5 mA g^{-1})	171 at 10 C (7.73 A g^{-1})	81% after 1000 cycles at 1.54 A g	S11
**USTB-6@G**	272	1.2–3.9	253 at 0.2 C	188 at 10 C	170 mAh g−1 after 6000 cycles at 5 C	S12
**BTT-ICTO@CNT-50**	404	1.0–3.6	396 at 0.1 A g^{-1}	227 at 5.0 A g^{-1}	184 mAh g-1 8000 cycles at 2.0 A g-1	S13

**PFG-1@rGO**	574	1.0–3.6	480 at 0.5 A g^{-1}	189 at 5 A g^{-1}	449 $mAhg^{-1}$ 1400 cycles at 0.5 Ag^{-1}	S14
**PPTODB @CNT**	341	1.5–3.5	185 at 0.02 A g^{-1}	98 at 1.5 A g^{-1}	135 mAh g^{-1} after 150 cycles at 0.02 A g^{-1}	S15
**HATN-PTO**	394	1.2–3.9	330 at 0.2 A g^{-1}	209 at 20 A g^{-1}	189 mAh g^{-1} after 500 cycles at 1 A g^{-1}	S16
**HCBHAT-PH@CNT**	436	1.2–4.0	303 at 0.25 C	171 at 8 C	93.8% at 500 mA g^{-1} after 200 cycles	S17
**HTPT-COF @CNT**	631	1.2–3.5	508 at 0.01 A g^{-1}	248 at 20 A g^{-1}	241 mAh g^{-1}, 73.6% at 2 A g^{-1} after 1000 cycles	S18
**TAT-AZO-COF**	486	1.2–4.4	465 at 0.1 A g^{-1}	215 at 20 A g^{-1}	160 mAh g^{-1} at 10 A g^{-1} after 2000 cycles	S19

6. Ex situ Infrared Spectroscopy

For reference, LiTFSI salt and a blank electrode containing only C65 and PVdF (75/25 wt.%) were measured to exclude any peaks potentially originating from these components. Additionally, a pellet with KBr and HAT-PTO powder was measured to observe any spectral changes when electrodes are prepared.

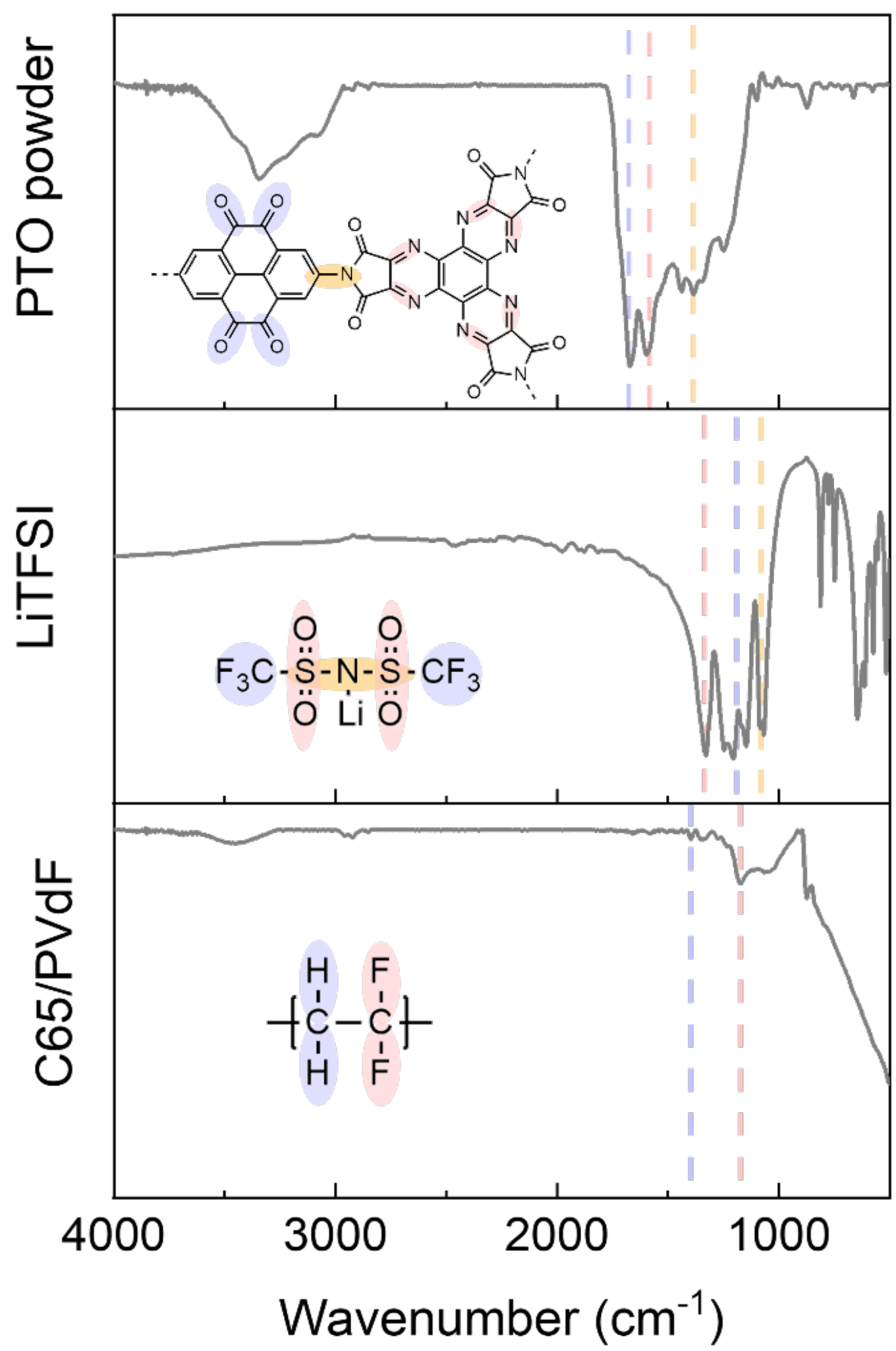

Figure S30. FT-IR spectra used as references. a) pristine **HAT-PTO** powder, b) LiTFSI salt, and c) blank electrode containing PVdF,

7. Mechanistic studies

The lithiation mechanism was investigated by cyclic voltammetry (CV) at various scan rates for both **HAT-PTO** and **HAT-PTO–cCNT**. The analysis employed the power-law relationship (**Equation S3**):

$$I = av^b$$

Where I represents the current peak, v the scan rate, and a and b the two unknown values to find.

Then the relationship between current (I) and the scan rate (v) was linearized in logarithmic form using **Equation S4**:

$$Log\ (I) = b \cdot Log\ (v) + Log\ (a)$$

The exponent b provides mechanistic insight: values close to 0.5 indicate diffusion-controlled processes, whereas values approaching 1.0 suggest a surface-controlled, pseudocapacitive behavior. The value of b was calculated through a linear fit of the two plots.[S20]

As shown in **Figures S31c** and **S31d**, **HAT-PTO** exhibits lower b values, pointing to a more diffusion-limited charge storage process. Conversely, the **HAT-PTO–cCNT** hybrid exhibits values near unity, highlighting a predominant capacitive contribution.

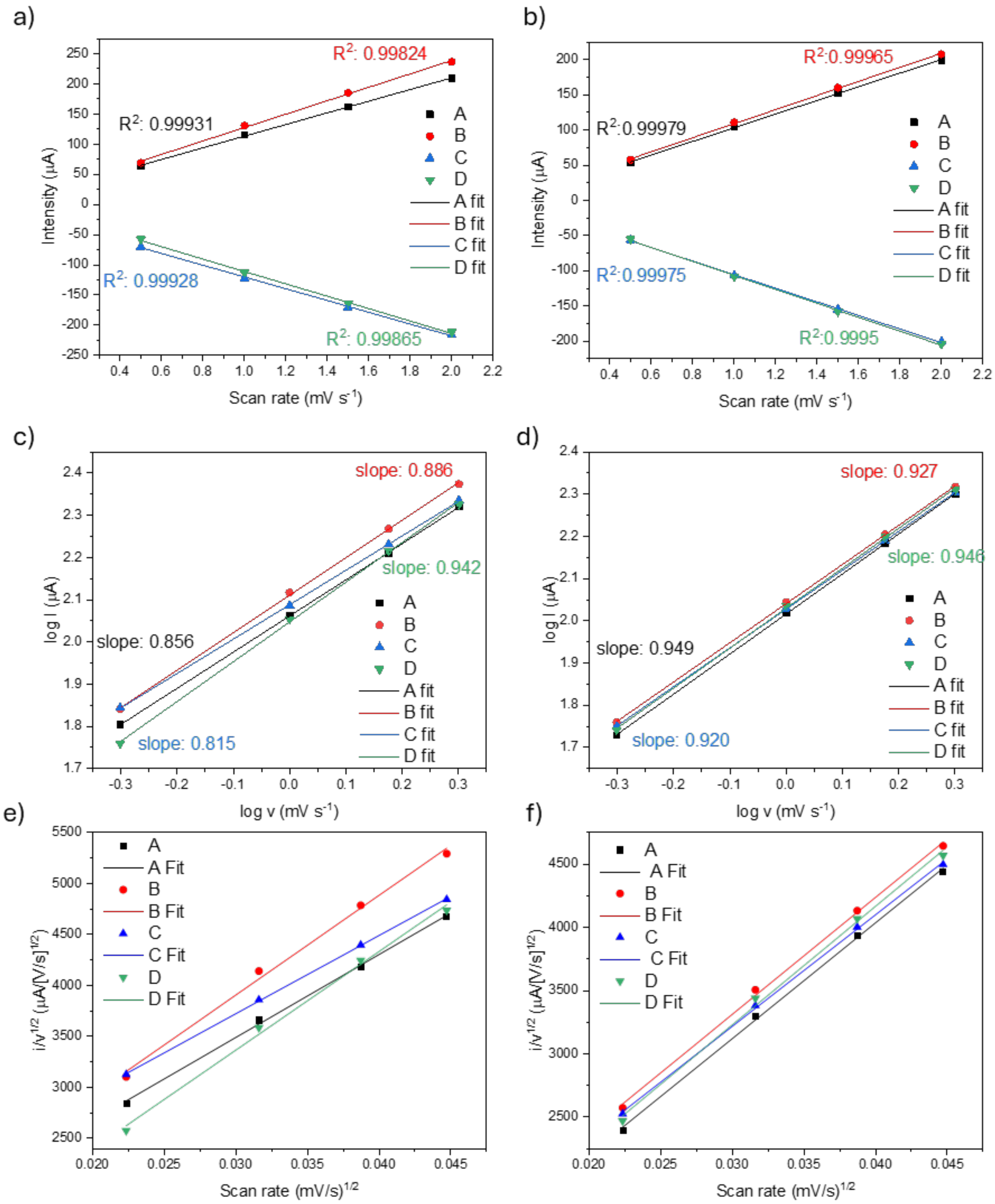

Figure S31. a) Intensity *vs* scan rates values and their linear fit for the four peaks of **HAT-PTO**. b) Intensity *vs* scan rates values and their linear fit for the four peaks of **HAT-PTO-cCNT**. c) Logarithmic scale of I *vs* logarithmic scale of the scan rate and their linear fit for the four peaks of **HAT-PTO**. d) Logarithmic scale of I *vs* logarithmic scale of the scan rate and their linear fit for the four peaks of **HAT-PTO-cCNT**. e) Intensity/square root of the scan rate vs the square root of the scan rate and their linear fit for the four peaks of **HAT-PTO**. f) Intensity/square root of the scan rate vs the square root of the scan rate and their linear fit for the four peaks of **HAT-PTO-cCNT**.

To complement this qualitative assessment, a quantitative analysis based on Dunn's method was conducted to separate capacitive and diffusion-controlled contributions in the two materials.[S21] in this method, the current response is analyzed at fixed potentials, where the capacitive current scales linearly with the scan rate (ν), while the diffusion-controlled current scales with the square root of the scan rate ($ν^{1/2}$). This relationship is described by **Equation S5:**

$$i_{total} = i_{capacitive} + i_{diffusive}$$

This expression can be rearranged as shown in **Equation S6**:

$$\frac{i(\nu)}{\nu^{1/2}} = k_1\,\nu^{1/2} + k_2$$

Here, k_1 (surface-controlled coefficient) and k_2 (diffusion-controlled coefficient) were obtained from the slope and intercept of the linear fits shown in **Figures S31e** and **S31f**, with the corresponding values summarized in **Table S5**.

Table S5. Values of k_1 and k_2 obtained applying Dunn's method through the linear fit shown in plots of Figures S22e and S22s for **HAT-PTO** and **HAT-PTO-cCNT**.

**Sample**	**Peak**	$\boldsymbol{k_2}$	$\boldsymbol{k_1}$
HAT-PTO	A	1042	81424
HAT-PTO	B	962	97938
HAT-PTO	C	1415	76775
HAT-PTO	D	454	96870
HAT-PTO–cCNT	A	365	91676
HAT-PTO–cCNT	B	530	92588
HAT-PTO–cCNT	C	561	88361
HAT-PTO–cCNT	D	405	93949

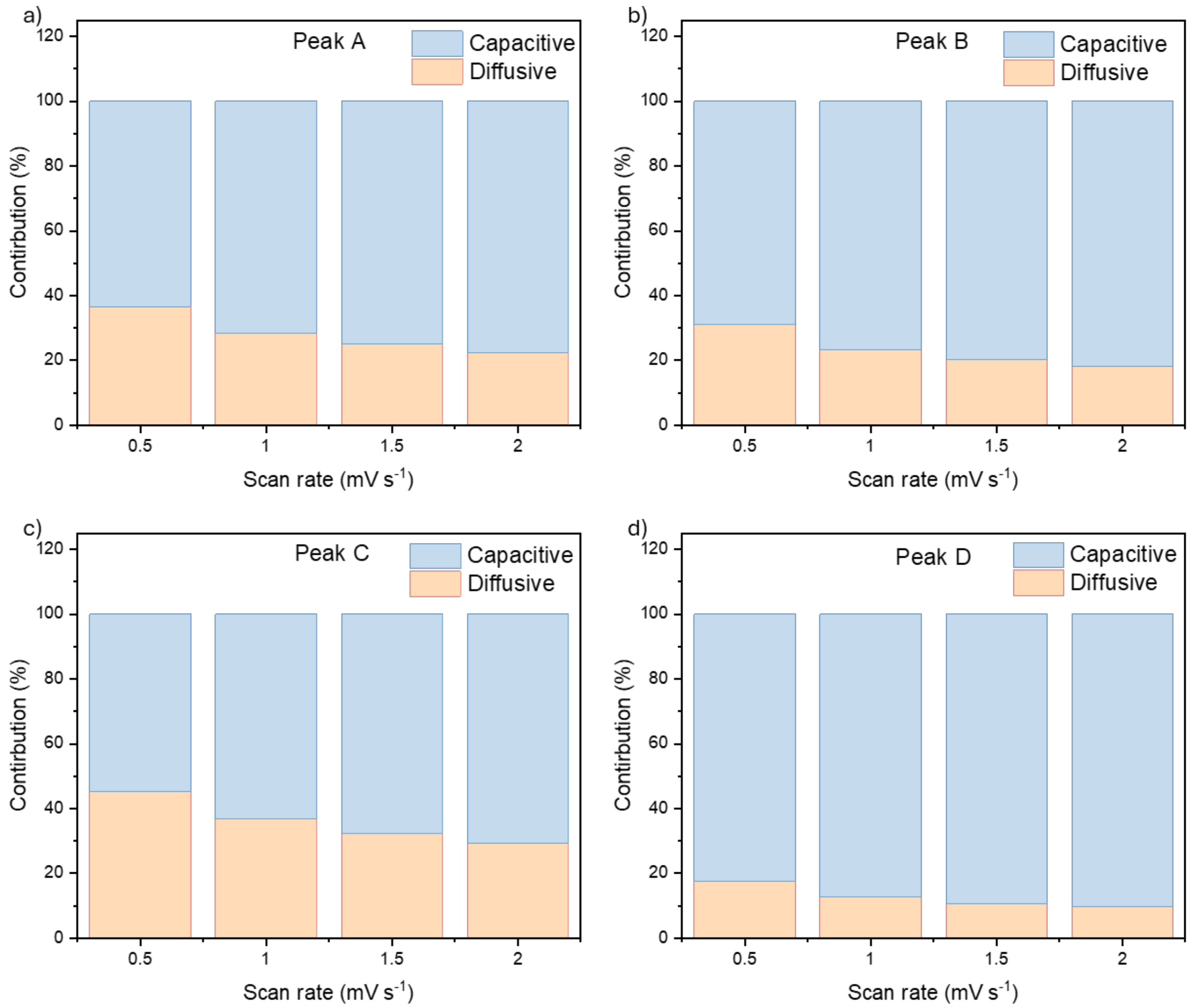

Figure S32. Capacitive and diffusive contributions in percentage (%) obtained for each redox peak (A, B, C, and D) of **HAT-PTO**.

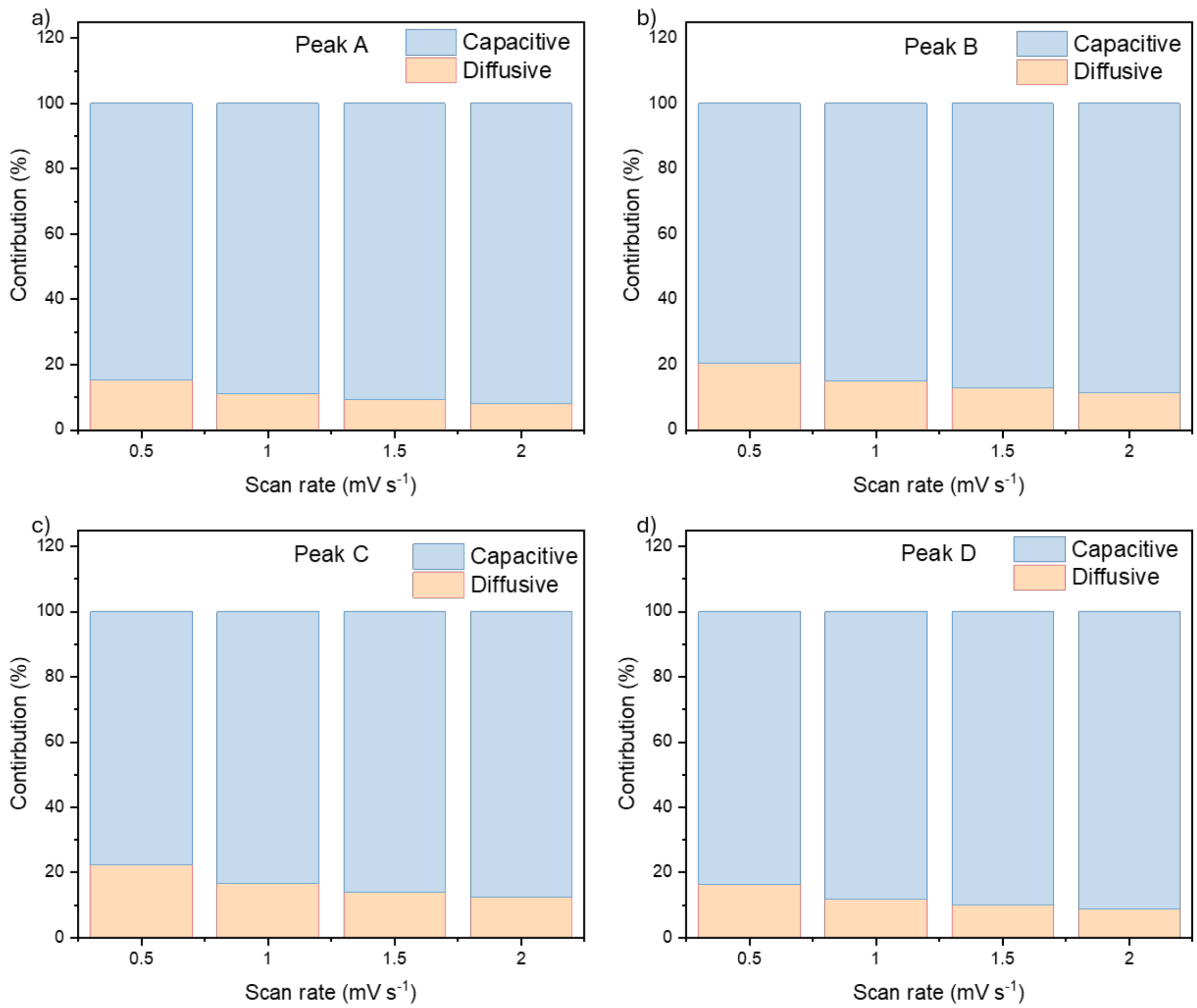

Figure S33. Capacitive and diffusive contributions in percentage (%) obtained for each redox peak (A, B, C, and D) of **HAT-PTOcCNT**.

Randles-Ševčík equation was used to calculate the diffusion coefficients of lithium ions in **HAT-PTO** according to **Equation S7:**[S22,S23]

$$I_p = 0.4463 \cdot nFAC\left(\frac{nFD\nu^{1/2}}{RT}\right)$$

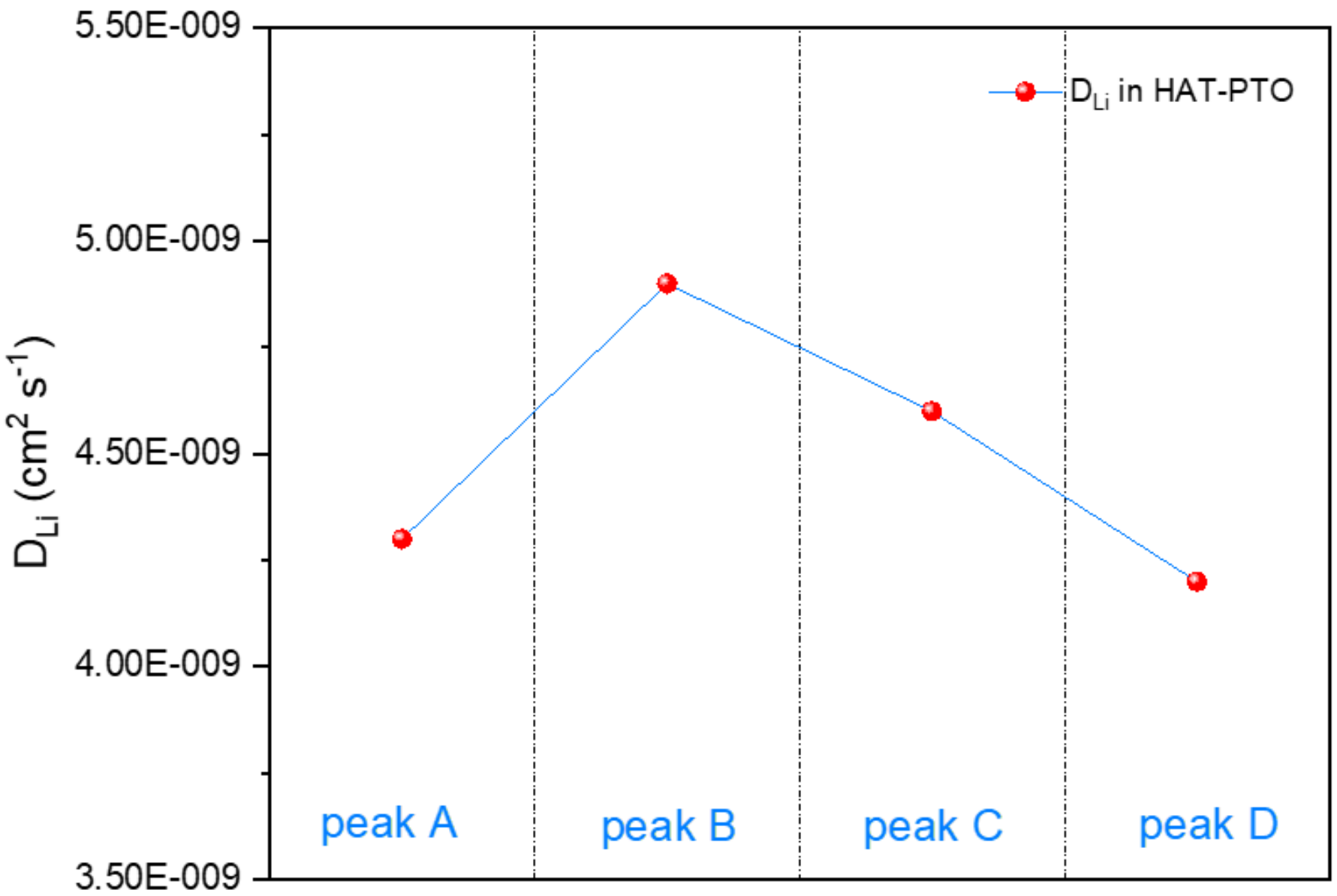

Figure S34. Diffusion coefficient of **HAT-PTO** calculated for each redox peak.

Table S6. Fitting parameters obtained from electrochemical impedance spectroscopy (EIS) measurements of **HAT-PTO** and **HAT-PTO-cCNT** samples.

Sample	Cycle	Equivalent circuit	R_e (Ω)	R_i (Ω)	χ^2
**HAT-PTO**	OCV	$R_e(R_iC_i)W$	0.8	148.1	$9x10^{-4}$
	10th	$R_e(R_iC_i)W$	0.4	18.4	$5x10^{-4}$
	20th	$R_e(R_iC_i)W$	0.4	20.02	$6x10^{-4}$
	30th	$R_e(R_iC_i)W$	0.4	14.6	$8x10^{-4}$
	40th	$R_e(R_iC_i)W$	0.4	16.6	$4x10^{-4}$
	100th	$R_e(R_iC_i)W$	0.4	23.3	$5x10^{-4}$
	300th	$R_e(R_iC_i)W$	0.4	65.8	$2x10^{-4}$
**HAT-PTO-cCNT**	OCV	$R_e(R_iC_i)W$	1.1	22.3	$1x10^{-4}$
	10th	$R_e(R_iC_i)W$	1.1	14.4	$6x10^{-4}$
	20th	$R_e(R_iC_i)W$	1.1	9.5	$4x10^{-4}$
	30th	$R_e(R_iC_i)W$	1.1	9.0	$1x10^{-4}$
	40th	$R_e(R_iC_i)W$	1.1	8.2	$2x10^{-4}$
	100th	$R_e(R_iC_i)W$	1.1	6.1	$1x10^{-4}$
	300th	$R_e(R_iC_i)W$	1.1	5.7	$2x10^{-4}$

8. Computational details

3D periodic DFT calculations were performed with the QuantumATK simulation package[S24,S25] with a unit cell containing one (eclipsed AA stacking), two (PD AA and AB stackings) and three (ABC stacking) molecular layers, to accommodate the different interlayer registries. Periodic calculations were initially performed at the LDA level and subsequently refined at the PBE level augmented with Grimme's D2 dispersion empirical correction.[S26] The medium numerical basis set was used in all cases. Molecular calculations were performed with the B3LYP and M06-2X Hamiltonians using the 6-31G(d,p) basis set with Gaussian16 C.[S27] For this, a molecular fragment, was cut out from an extended DFT flat model, and saturated with hydrogens yielding a $C_{188}H_{28}N_{54}O_5$ molecule with 326 atoms. This molecule was optimized enforcing planar symmetry with the hybrid B3LYP Hamiltonian. Then, several representative lithium adsorption sites adding up to more than 70 different separated computations were explored. Initially, the Li atom positions were optimized with an eXtended Tight Binding (xTB) Hamiltonian and later refined with DFT (M06-2X) on a frozen substrate. The so-obtained lithium positions were then combined and optimized together with the central region of the COF model with the same Hamiltonian while the four outer regions, representing the embedding on the extended 2D HAT-PTO monolayer, were constrained, Figure S35. The adsorption energies were then computed and energy-ranked to quantify the thermodynamical probability of different Li insertions for the HAT-PTO.

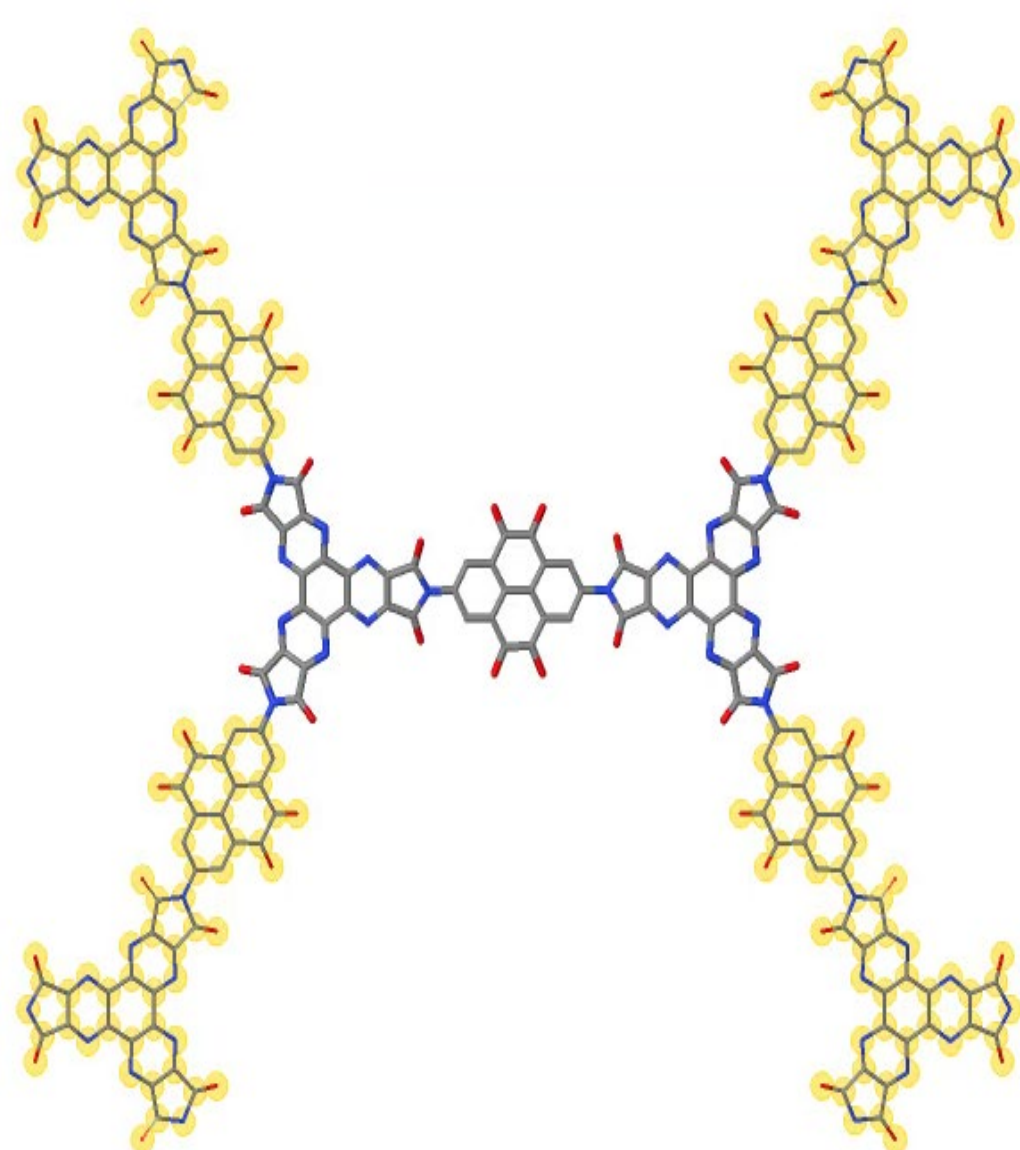

Figure S35. HAT-PTO monolayer $C_{188}H_{28}N_{54}O_5$ molecular analogue model, the constrained, frozen, region is highlighted in yellow.

9. References

[S1] K. Kanakarajan, A. W. Czarnik. Synthesis and some reactions of hexaazatriphenylenehexanitrile, a hydrogen-free polyfunctional heterocycle with D3h symmetry. *J. Org.Chem.* **1986**, *51*, 5241-5243.

[S2] B. Boukamp. A package for impedance/admittance data analysis. *Solid State Ion,* **1986**, *18–19*, 136.

[S3] B. Boukamp. A Nonlinear Least Squares Fit procedure for analysis of immittance data of electrochemical systems. *Solid State Ion,* **1986**, *20*, 31.

[S4] R. Narayan, A. Blagojević, G. Mali, J. Fredy, V. Santa, J. Bitenc, A. Randon-Vitanova, R. Dominko. Nanostructured Poly(hydroquinonyl-benzoquinonyl sulfide)/Multiwalled Carbon Nanotube Composite Cathodes: Improved Synthesis and Performance for Rechargeable Li and Mg Organic Batteries. *Chem. Mater,* **2022**, *34*, 6378,

[S5]. X. Xu, S. Zhang, K. Xu, H. Chen, X. Fan, N. Huang, Janus Dione-Based Conjugated Covalent Organic Frameworks with High Conductivity as Superior Cathode Materials. *J. Am. Chem. Soc.*, **2022**, *145*, 1022-1030.

[S6] X. Yang, L. Gong, X. Liu, P. Zhang, B. Li, D. Qi, K. Wang, F. He, J. Jiang, Mesoporous Polyimide-Linked Covalent Organic Framework with Multiple Redox-Active Sites for High-Performance Cathodic Li Storage. *Angew. Chem. Int. Ed.*, **2022**, *61*, e202207043.

[S7] H. Gao, A. R. Neale, Q. Zhu, M. Bahri, X. Wang, H. F. Yang, Y. J. Xu, R. Clowes, N. D. Browning, M. A. Little, L. J. Hardwick, A. I. Cooper, A Pyrene-4,5,9,10-Tetraone-Based Covalent Organic Framework Delivers High Specific Capacity as a Li-Ion Positive Electrode. *J. Am. Chem. Soc.*, **2022**, *144*, 9434-9442.

[S8] X. Yang, Y. Hu, N. Dunlap, X. Wang, S. Huang, Z. Su, S. Sharma, Y. Jin, F. Huang, X. Wang, S.-h. Lee, W. Zhang, Truxenone-based Covalent Organic Framework as an All-Solid-State Lithium-Ion Battery Cathode with High Capacity. *Angew. Chem. Int. Ed.* **2020**, *59,* 20385–20389.

[S9] Z. Luo, L. Liu, J. Ning, K. Lei, Y. Lu, F. Li, J. Chen, A Microporous Covalent-Organic Framework with Abundant Accessible Carbonyl Groups for Lithium-Ion Batteries. *Angew. Chem. Int. Ed.* **2018**, *57,* 9443–9446.

[S10] S. Wang, Q. Wang, P. Shao, Y. Han, X. Gao, L. Ma, S. Yuan, X. Ma, J. Zhou, X. Feng, B. Wang, Exfoliation of Covalent Organic Frameworks into Few-Layer Redox-Active Nanosheets as Cathode Materials for Lithium-Ion Batteries. *J. Am. Chem. Soc.* **2017**, *139,* 4258–4261.

[S11] M. Wu, Y. Zhao, B. Sun, Z. Sun, C. Li, Y. Han, L. Xu, Z. Ge, Y. Ren, M. Zhang, Q. Zhang, Y. Lu, W. Wang, Y. Ma, Y. Chen, A 2D covalent organic framework as a high-performance cathode material for lithium-ion batteries. *Nano Energy* **2020**, *70*, 104498.

[S12] X. Liu, Y. Jin, H. Wang, X. Yang, P. Zhang, K. Wang, J. Jiang, In Situ Growth of Covalent Organic Framework Nanosheets on Graphene as the Cathode for Long-Life High-Capacity Lithium-Ion Batteries. *Adv. Mater.,* **2022,** *34*, 2203605.

[S13] N. Fu, Y. Liu, K. Kang, X. Tang, S. Zhang, Z. Yang, Y. Wang, P. Jin, Y. Niu, B. Yang, Fully sp^2 Carbon-Conjugated Covalent Organic Frameworks with Multiple Active Sites for Advanced Lithium-Ion Battery Cathodes. *Angew. Chem. Int. Ed.* **2024**, *63*, e202412334.

[S14] X. Li, H. Wang, H. Chen, Q. Zheng, Q. Zhang, H. Mao, Y. Liu, S. Cai, B. Sun, C. Dun, M. P. Gordon, H. Zheng, J. A. Reimer, J. J. Urban, J. Ciston, T. Tan, E. M. Chan, J. Zhang, Y. Liu, Dynamic Covalent Synthesis of Crystalline Porous Graphitic Frameworks. *Chem* **2020**, *6*, 933-944.

[S15] C.-J. Yao, Z. Wu, J. Xie, F. Yu, W. Guo, Z. J. Xu, D.-S. Li, S. Zhang, Q. Zhang, **Two-**Dimensional (2D) Covalent Organic Framework as Efficient Cathode for Binder-free Lithium-Ion Battery. *ChemSusChem* **2020**, *13*, 2457-2463.

[S16] Q. Bai, J. Huang, K. Tang, Y. Zhu, D. Wu, Arylamine-Linked Porous Organic Polymers with Abundant Redox-Active Sites as High-Capacity and High-Rate Organic Cathodes for Lithium-Ion Batteries. *Adv. Mater.* **2025**, *37*, 2416661.

[S17] P. Xu, F. Gao, D. Liu, Hexaazatriphenylene Based Polyimide with Dense Dual Redox Sites as a High-Performance Organic Cathode for Lithium-Ion Batteries. *Adv. Mater. Interfaces* **2023**, *10*, 2300464.

[S18] Y. Wang, J. Wang, J. Peng, Y. Jiang, Y. Zhu, Y. Yang, Crafting Core–Shell Heterostructures with Enriched Active Centers for High-Energy-Density Symmetric Lithium-Ion Batteries. *ACS Nano* **2024**, *18*, 23958–23967.

[S19] W. Feng, Z. Yang, C. Guo, H. Zhuang, R. Xu, H. Zhang, M. Shi, Z. Chen, Y. Chen, Y.-Q. Lan, Three-Motif Molecular Junction Covalent Organic Frameworks for Symmetric All-Organic Lithium-Ion Battery. *Angew. Chem. Int. Ed.* 2025, e202508937.

[S20] B. E. Conway, *Electrochemical Supercapacitors,* **1999**, *Scientific Fundamentals and Technological Applications.* http://dx.doi.org/10.1007/978-1-4757-3058-6.

[S21] J. Wang, J. Polleux, J. Lim, B. Dunn. Pseudocapacitive Contributions to Electrochemical Energy Storage in TiO2 (Anatase) Nanoparticles. *Phys. Chem. C,* **2007**, 111, 14925–14931.

[S22] J. E. B. Randles. Kinetics of rapid electrode reactions. *Discuss Faraday Soc* **1947**, *1*, 11.

[S23] A. Ševčík. Oscillographic polarography with periodical triangular voltage. *Collect Czechoslov Chem Commun* **1948**, *13*, 349.

[S24] S. Smidstrup, T. Markussen, P. Vancraeyveld, J. Wellendorff, J. Schneider, T. Gunst, B. Verstichel, D. Stradi, P. A. Khomyakov, U. G Vej-Hansen. QuantumATK: an integrated

platform of electronic and atomic-scale modelling tools *J. Phys.: Condens. Matter* **2020**, *32*, 015901.
[S25] QuantumATK, version P-2022.12, https://www.synopsys.com/silicon/quantumatk.html
[S26] S.Grimme, J. Antony, S. Ehrlich, H. Krieg. A consistent and accurate ab initio parametrization of density functional dispersion correction (DFT-D) for the 94 elements H-Pu *J. Chem. Phys.* **2010**, *132*, 154104.
[S27] Gaussian 16, Revision C.01, M. J. Frisch, G. W. Trucks, H. B. Schlegel, G. E. Scuseria, M. A. Robb, J. R. Cheeseman, G. Scalmani, V. Barone, G. A. Petersson, H. Nakatsuji, X. Li, M. Caricato, A. V. Marenich, J. Bloino, B. G. Janesko, R. Gomperts, B. Mennucci, H. P. Hratchian, J. V. Ortiz, A. F. Izmaylov, J. L. Sonnenberg, D. Williams-Young, F. Ding, F. Lipparini, F. Egidi, J. Goings, B. Peng, A. Petrone, T. Henderson, D. Ranasinghe, V. G. Zakrzewski, J. Gao, N. Rega, G. Zheng, W. Liang, M. Hada, M. Ehara, K. Toyota, R. Fukuda, J. Hasegawa, M. Ishida, T. Nakajima, Y. Honda, O. Kitao, H. Nakai, T. Vreven, K. Throssell, J. A. Montgomery, Jr., J. E. Peralta, F. Ogliaro, M. J. Bearpark, J. J. Heyd, E. N. Brothers, K. N. Kudin, V. N. Staroverov, T. A. Keith, R. Kobayashi, J. Normand, K. Raghavachari, A. P. Rendell, J. C. Burant, S. S. Iyengar, J. Tomasi, M. Cossi, J. M. Millam, M. Klene, C. Adamo, R. Cammi, J. W. Ochterski, R. L. Martin, K. Morokuma, O. Farkas, J. B. Foresman, and D. J. Fox, Gaussian, Inc., Wallingford CT, **2019**.
